# Energetic evolution of cellular Transportomes

**DOI:** 10.1186/s12864-018-4816-5

**Published:** 2018-05-30

**Authors:** Behrooz Darbani, Douglas B. Kell, Irina Borodina

**Affiliations:** 1 The Novo Nordisk Foundation Center for Biosustainability, Technical University of Denmark, 2800 Lyngby, Denmark; 2 School of Chemistry & Manchester Institute of Biotechnology, The University of Manchester, 131 Princess St, Manchester, M1 7DN UK

**Keywords:** Energetic efficiency, Evolution, Cellular membrane, Mitochondria, Transporters

## Abstract

**Background:**

Transporter proteins mediate the translocation of substances across the membranes of living cells. Many transport processes are energetically expensive and the cells use 20 to 60% of their energy to power the transportomes. We hypothesized that there may be an evolutionary selection pressure for lower energy transporters.

**Results:**

We performed a genome-wide analysis of the compositional reshaping of the transportomes across the kingdoms of bacteria, archaea, and eukarya. We found that the share of ABC transporters is much higher in bacteria and archaea (ca. 27% of the transportome) than in primitive eukaryotes (13%), algae and plants (10%) and in fungi and animals (5–6%). This decrease is compensated by an increased occurrence of secondary transporters and ion channels. The share of ion channels is particularly high in animals (ca. 30% of the transportome) and algae and plants with (ca. 13%), when compared to bacteria and archaea with only 6–7%. Therefore, our results show a move to a preference for the low-energy-demanding transporters (ion channels and carriers) over the more energy-costly transporter classes (ATP-dependent families, and ABCs in particular) as part of the transition from prokaryotes to eukaryotes. The transportome analysis also indicated seven bacterial species, including *Neorickettsia risticii* and *Neorickettsia sennetsu*, as likely origins of the mitochondrion in eukaryotes, based on the phylogenetically restricted presence therein of clear homologues of modern mitochondrial solute carriers.

**Conclusions:**

The results indicate that the transportomes of eukaryotes evolved strongly towards a higher energetic efficiency, as ATP-dependent transporters diminished and secondary transporters and ion channels proliferated. These changes have likely been important in the development of tissues performing energetically costly cellular functions.

**Electronic supplementary material:**

The online version of this article (10.1186/s12864-018-4816-5) contains supplementary material, which is available to authorized users.

## Background

The expansion of life on Earth has involved competition and also cooperation among organisms for the utilisation of resources which have been accessible to them [1, 2]. Arguments have been made in favour of growth rate over growth efficiency in organisms competing within a specific niche [3], which implies a natural selection towards an improved ability to capture and utilize the available free energy sources for survival and reproduction [4, 5]. Darwinian evolutionary theory originally covered only phenotypic improvements at the organismal level, but we nowadays also recognize the importance of molecular and cellular events such as the acquisition of mitochondria by eukaryotes. This enabled an increase of eukaryotic cell size and complexity due to a more “efficient” generation of the cellular fuel ATP [6, 7]. Cells need to allocate considerable resources to energize their transportomes. For example, brain neurons account for approximately 20% of the basal metabolic rate in humans, mostly for the movement of ions across neuronal membranes [8]. In general, a metabolic cost of up to 60% of the total ATP requirement in organisms is estimated for the activity of their transportomes [9, 10]. Thus, it would be reasonable to imagine that an improved energetic performance of the transportome has contributed to a higher fitness over the course of evolution.

Despite the importance of cellular transportomes (also reported as the second largest component of the human membrane proteome [11]), transporters are surprisingly understudied [12]. Additionally, the presence of substrate-binding proteins as the partners of a subset of membrane transporters [13] makes the cellular transport machinery more complicated than if we consider only the transporters themselves. The tightening of porous and leaky primordial envelopes such that they did not let in (and could learn to efflux) all kinds of substances [14, 15] has been proposed as a turning point for membrane transporters to co-evolve together with lipid bilayer membranes [16]. Different classes of transporters, each including several transporter superfamilies, share a common ancestral family of peptides which carry 1–4 transmembrane domains and form homo- and hetero-oligomer channels [17–20]. Intragenic duplication and triplication have been the major events promoting the diversification of transporter proteins [18, 20]. Classical evolutionary theory on the basis of natural selection proposed by Charles Darwin [4, 5] explains how the random variability of the genome as the diversification force has given the chance for specialisation, improved performance, and adaptation to the continuously changing biosphere. Here, we annotated the cellular transportomes of bacteria, archaea and eukarya and analysed their composition with a focus on the energetic efficiency. The analyses include all the three classes of transporters, i.e.*,* ion channels, secondary transporters, and ATP-dependent transporters. To translocate substrates, ATP-dependent transporters bind and hydrolyse ATP [21], ion channels form pores for selective diffusion of ions and small molecules, and the secondary transporters shuttle substrate molecules across biological membranes either through energy independent facilitated diffusion or via exploiting membrane electrochemical potential gradients through uniport, symport and antiport [22].

## Results

**Fig. 1:**
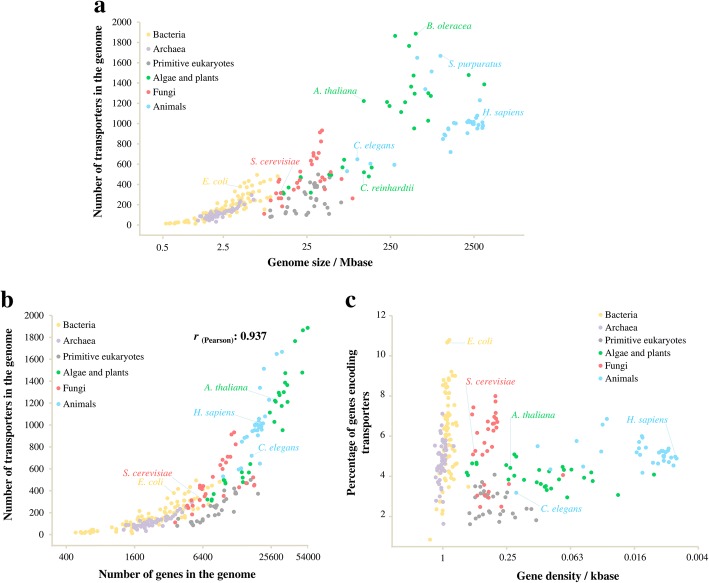
Transportomes differ in size among species and evolutionarily distant domains of life. **(a)** The number of membrane transporters per organism in relation to the genome size. **(b)** The number of membrane transporters per genome in relation to the number of total genes. **(c)** Percentage of transporter-coding genes in relation to gene density

To study the compositional changes of transportomes, we analysed the transportomes of 249 evolutionarily distant species (of which 222 were annotated in this study) from archaea, bacteria and eukarya. These included 126 prokaryotic species (from 16 taxonomic phyla and 60 taxonomic orders), 30 primitive eukaryotes (different species from Alveolata, Kinetoplastida and Amoebozoa), 30 algal and plant species, 30 fungal species, and 33 animal species (See Additional file 2: Data S1). The transportomes were annotated using the Transporter Automatic Annotation Pipeline at TransportDB [23]. Notably, the species had large differences in the size of both their genomes and their transportomes (Fig. 1a, b and Additional file 2: Data S1).

We found that the sizes of an organism’s transportome tends to increase with its genome size, though there are considerable intra-kingdom variations (Fig. 1a) (see also [24]). In particular, primitive eukaryotes have small transportomes with 100–500 members, relative to their genome sizes of tens of Mb when compared to the bacteria with modestly sized genomes (less than 10 Mb). Ion channels, secondary transporters, and primary active transporters were found in each of the analysed domains of life (See Additional file 2: Data S1). This indicates a very early appearance for these three classes of transporters, possibly in a common ancestor.

**Fig. 2:**
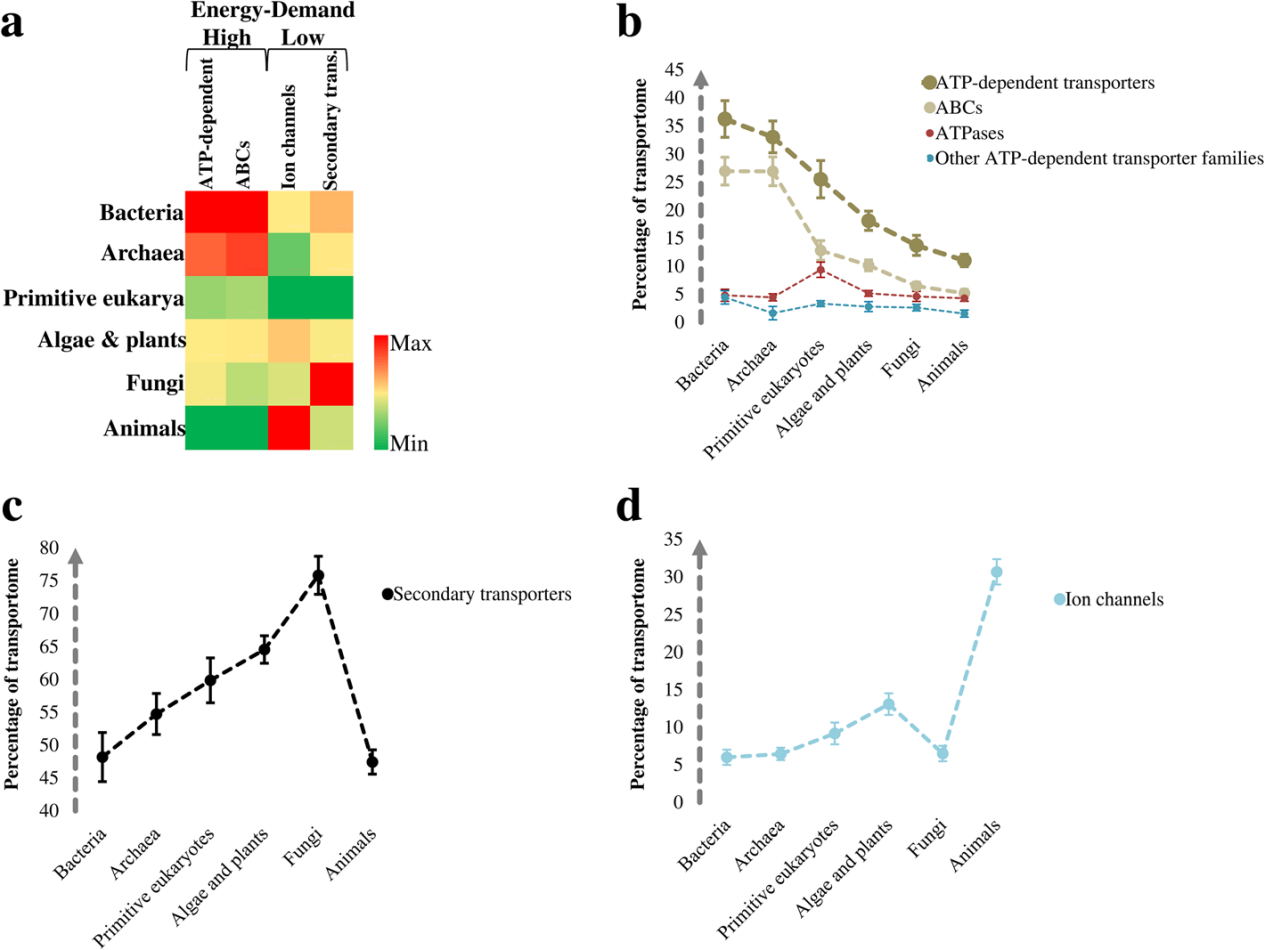
The evolutionary dynamics of transportomes composition. **(a)** Heat-map representation of the changes in the number of members of the transporter classes. To calculate the intra-genomic frequencies, the numbers of transporter members are normalized to the total number of genes per genome. The heat-map is drawn for each class of ion channels, secondary transporters, and ATP-dependent transporters and therefore colors are not comparable between the classes. **(b)** The fraction of ATP-dependent transporters in the transportomes. All of the variations of ATP-dependent transporters and ABC superfamily except the difference between bacteria and archaea are significant with a *p*-value less than 0.001. **(c)** The fraction of secondary transporters in the transportomes. Only the difference between bacteria and animals is not statistically significant (*p* = 0.635). **(d)** The fraction of ion channels in the transportomes. All differences, except among fungi, bacteria and archaea, are significant with a *p*-value less than 0.001. The values on panels b-d are shown as mean +/− t-test based 99% confidence intervals. The variations were also confirmed on the arc sin √*x* transformed data (See Additional file 2: Data S1). The family names of the transporters can also be found in the Additional file 2: Data S1

In agreement with previous reports [25, 26], the genome size had a higher rate of enlargement than did the gene number, resulting in a decreased gene density over the course of evolution (Fig. 1c). The transportome enlargement was found to be well correlated with the increase in the gene number (*r*_(Pearson)_ = 0.937) with the exception of primitive eukarya, where the transportome-encoding proportion of genes was the lowest (Fig. 1b, c). Of most interest, the composition of the transportomes changed from prokaryotes to eukaryotes and also among the eukaryotic kingdoms along with the transportome enlargement (Fig. 2a). Specifically, we found higher intra-genomic frequencies (frequency relative to the total number of genes in the genome) of ATP-dependent transport classes in prokaryotes than eukaryotes. An opposing trend was found for low-energy-demanding transport classes. This indicates different rates of gene proliferation among the evolved transporter classes; low-energy-demanding transporter families have expanded at a higher rate. Notably, the transportomes of primitive eukaryotes also represent a transition state between prokaryotic and higher eukaryotic kingdoms (Fig. 2a).

We further compared the prevalence of secondary transporters, ion channels, and ATP-dependent transporters within the transportomes of each species, and averaged these over the larger taxonomic groups (Fig. 2b-d). In general, we observed compositional changes that indicate a positive adaptive selection of the no- to low-cost-flux equilibrative ion channels and carriers, and negative selection of the energetically more expensive ABC transporters. More specifically, we found significant variations in each of the transporter classes (Fig. 2b-d). While 27% of all the bacterial and archaeal transporters are ABC transporters, this fraction decreases to 13% in primitive eukaryotes, 10% in algae and plants and a mere 5–6% in fungi and animals (Fig. 2b). On the other hand, an increased contribution to the cellular transportome was found for secondary transporters in eukaryotes, particularly in fungi (Fig. 2c). Ion channels accounted for only 6–7% of bacterial and archaeal transportomes, but were more abundant in algae and plants (≈ 13%) and particularly in animals (≈ 30%) (Fig. 2d). During evolution, and in parallel with the genome enlargement and gene pool expansion, each of the three classes of transporters had a chance to contribute proportionally to the expansion of transportomes. By contrast, our results show a preference for the low-energy demanding transporters (ion channels and carriers) over the energy-costly transporter classes (ATP-dependent families, and ABCs in particular) in the transition from prokaryotes to eukaryotes.

We defined the energy usage efficiency of a transportome (EUE) as the average required energy per single substrate translocation. We calculated the EUE values for the transportomes studied (more details in the Methods section). The EUE describes the overall energetic performance of transportomes at organismal level and most importantly it does not indicate the total energy consumption by the cellular transportome, because the latter depends also on the flux through individual transporters that is largely unknown. In contrast to the total energy requirements, the EUE is therefore not subjected to spatiotemporal variations. By comparing the average EUEs of the transportomes across the different domains of life, we found that the EUE has improved in eukaryotes by reductions of up to 0.50 ATP in the average ATP consumption per single transport event mediated by transporters (Table 1).

**Table 1:** Improvement in the energy-usage efficiency (ΔEUE) calculated as changes in the average ATP-usage per single transport cycle

Domains of life	Bacteria	Archaea	Primitive eukaryotes	Algae and plants	Fungi	Animals
Bacteria	0					
Archaea	−0.03	0				
Primitive eukaryotes	−0.16	− 0.14	0			
Algae and plants	−0.27	−0.25	− 0.11	0		
Fungi	−0.30	−0.27	− 0.13	−0.02	0	
Animals	−0.49	−0.46	− 0.32	−0.21	− 0.19	0

The changes (ΔEUE) are calculated as ATP-usage_domain of life in the matrix-row_ – ATP-usage_domain of life in the matrix-column_ (see methods)

Negative changes represent the reduction in ATP-usage and improved EUE of transportomes

**Fig. 3:**
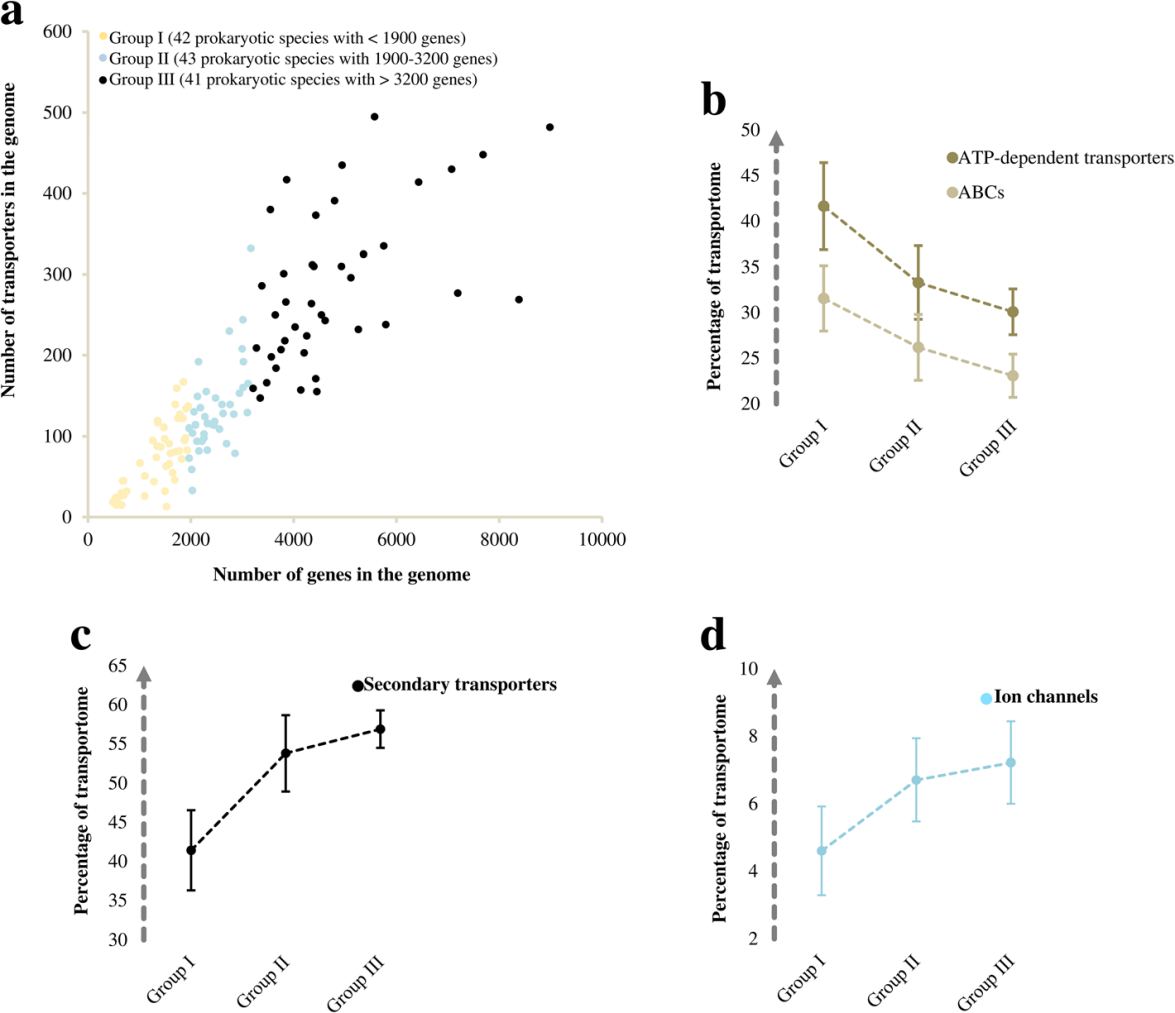
The compositional changes in the transportomes of prokaryotes with different genome sizes. **(a)** Comparison between the transportome size and total gene number among prokaryotes including bacteria and archaea. All of the 126 studied species are clustered into three groups based on the total number of the genes. **(b)** The fraction of ATP-dependent transporters in the transportomes. **(c)** The fraction of secondary transporters in the transportomes. **(d)** The fraction of ion channels in the transportomes. The values on panels b-d are shown as mean +/− t-test based 99% confidence interval. The variations were also confirmed on the arc sin √*x* transformed data (See Additional file 2: Data S1). Group I and III differ significantly for all of the transporter classes with a *p*-value of < 0.001

Furthermore, the data suggest that animals have mainly relied on the diversification of ion channels, fungi on secondary transporters, and finally, algae and plants on both transporter classes for the evolution of their transportomes (Fig. 2b-d). For energetically efficient transportomes, organisms therefore adopted different strategies, likely due to their specialisations and different requirements. In fact, this recalls cooperative evolution at tissue and molecular levels (see also [2, 27]). The trend of expansion of ion channels and secondary transporters at the expense of ATP-dependent transporters also holds true for the prokaryotic transportomes (Fig. 3), even though they did not undergo the kind of intensive developmental specialisation as was required in multicellular eukaryotes.

**Fig. 4:**
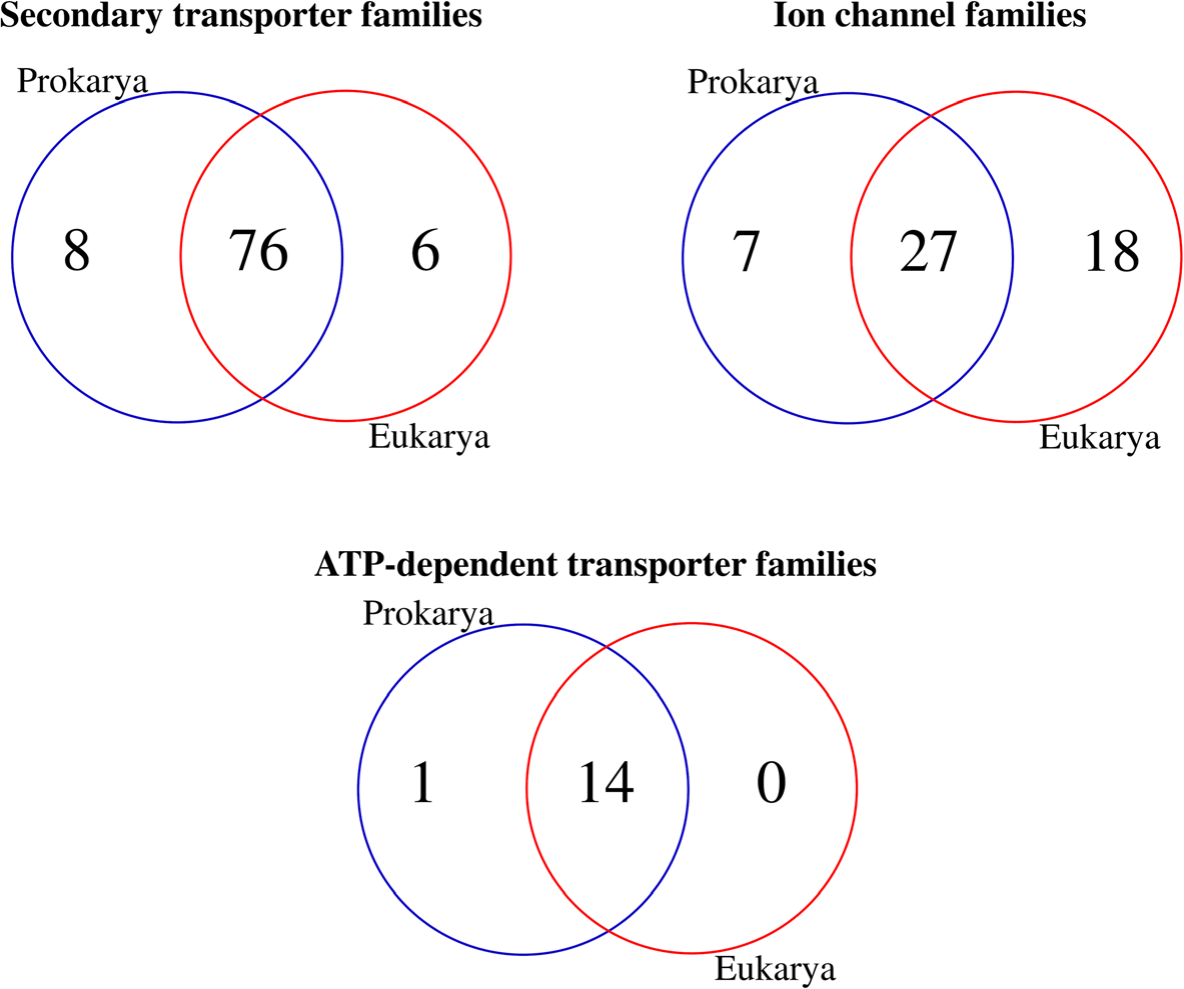
Number of transporter families that are shared between or are specific for prokaryotes and eukaryotes

The analyses also indicate family member expansions for secondary transporters (from 60 to 100 in prokaryotes to more than 400 in animals and 600 in plants on average) and ion channels (from 10 members in prokaryotes to more than 300 members in animals). To find prokaryotic- and eukaryotic-specific families, we further enriched our analysis by including the publicly-available data for the secondary transporters and ion channels of prokaryotes found at TransportDB 2.0 (http://www.membranetransport.org/transportDB2/index.html). This extended our prokaryotes to 2637 transportomes (Additional file 1: Table S1). We also included two eukaryotic transportomes from diatoms found at TransportDB 2.0 (Additional file 1: Table S1). When comparing the organisms for the presence of different transporter families, we found that eight secondary transporter families were completely lost in the passage to eukaryotes (Fig. 4). This includes the 2-hydroxycarboxylate transporter family, the p-aminobenzoyl-glutamate transporter family, the short chain fatty acid uptake (AtoE) family, the monovalent cation (K^+^ or Na^+^):proton antiporter-3 family, the branched chain amino acid:cation symporter family, the NhaB Na^+^:H^+^ antiporter family, the riboflavin transporter family, and the Na^+^-dependent bicarbonate transporter family (See Additional file 2: Data S1). Additionally, we found that six new families of secondary transporters had emerged in eukaryotes (Fig. 4, See also Additional file 2: Data S1). Specifically, we did not find any prokaryotic hits in GenBank for these eukaryotic families, that must have diverged massively [28] from some ancestral genes. Our results are mostly in agreement with the intra-family speciation of transporters reported by Ren and Paulsen [29]. In contrast, we also found that ion channels had evolved through both intra-family expansions and the substantial appearance of new families. While there were only seven ion channel families specific to the prokaryotes, there are 18 eukarya-specific families (Fig. 4, See also Additional file 2: Data S1). Interestingly, the mitochondria-specific solute carriers, i.e., solute carrier family SLC25 [30, 31], are absent from the transportomes of all 143 archaeal species (See Additional file 1: Table S1 for the list of organisms). Among the bacteria, including 259 alpha-proteobacteria, of which 69 belonged to the order *Rickettsiales*, we found only seven bacterial genomes that encoded members of the mitochondrial transporter family. These are *Neorickettsia risticii*, *Neorickettsia sennetsu*, *Legionella pneumophila*, *Legionella longbeachae*, *Acidaminococcus intestini*, *Cardinium endosymbiont* and *Butyrivibrio proteoclasticus*. The presence of the mitochondrial transporter family members indicates that these seven bacterial species are possible origins of the mitochondrion in eukaryotes. The first two species are Gram-negative obligatory intracellular bacteria from the order *Rickettsiales*, an order that in previous studies was proposed (based on different evidence) as the most likely origin of the mitochondrion in eukaryotes [28, 30–35]. The present findings add significant evidence to this proposal.

## Discussion

Our genome-wide analysis on the compositional reshaping of the transportomes across the kingdoms of bacteria, archaea, and eukarya suggests an evolutionary preference for energetically efficient transportomes. Our analyses also mapped some ancestral mitochodrial carrier members of SLC25 into the bacterial genomes and excluded the archaeal species. These ancesters include two bacteria from the order *Rickettsiales. Rickettsiales* are considered to be the mitochodrial origin [32–35]. It seems the eukaryotic members of the SLC25 family, all with 6 transmembrane domains, have diverged massively from their bacterial ancesters with 4–5 transmembrane domains. In agreement with Calvo et al. [36], the absence of SLC25 homologues in the genome of *Rickettsia prowazekii*, a species from the *Rickettsiales*, was also confirmed in this study; we did not find any ancestral SLC25 coding genes among the 10 different strains of *Rickettsia prowazekii* that were included in the analysis.

A higher ratio of secondary transporters to primary ATP-driven transporters was previously reported for yeast when compared to prokaryotes [37]. This is additionally complemented by a relatively large genomic survey on 141 species, of which 9 species were eukaryotic [29]. Our results on the general compositional changes of transportomes are in agreement with the findings of Ren and Poulsen [29]. The publication by Ren and Poulsen, however, did not report the energetics of transportomes or the detailed analysis of transporter classes. Resource depletion and inadequate nutrition affect survival but can also increase energetic efficiency, that is fitness, in the course of evolution [38]. For example, the adaptation to chronic energy stress has been hypothesized to be partly responsible for the general capacity of archaea to out-compete bacteria [39]. In addition, energetic evolution of gene expression has also been discussed by addressing the preference for major codons in highly expressed genes, which alleviates the costly processes of proofreading and removal of dysfunctional proteins [40]. Another environmental stress reported to affect codon usage is the low availability of nitrogen. Marine microorganisms faced with low levels of nitrogen show reduced levels of nitrogen in amino acid sequences, especially in highly expressed proteins, which reduces the total cellular nitrogen budget by up to 10% [41]. There are also observations on the energetic evolution at a phenotypic level. For example, an energetic trade-off between maximum population density and body size has been reported in [42]. Energetic constraints have also been proposed for the number of neurons, which determines the brain size [43]. At a molecular level, although both the solute carrier SLC2 family of facilitated glucose transporters and the SLC5 family of energy-dependent sodium/glucose cotransporters participate in glucose uptake, the human brain has largely been dependent on SLC2 transporters for the energetically free supply of glucose [44]. Positive selection and gene expression adjustments, such that they allocate higher energy fluxes to the brain, have been reported for the SLC2 members in the human branch that has a larger brain compared to the chimpanzees and macaques [45]. This indicates the importance of the energetic evolution of membrane transporters, even at the single family level, and highlights the possibly huge impacts of the energetic evolution of the entire transportomes on organismal adaptation and speciation. On the other hand, the higher dependency of bacterial and archaeal species on the ATP-driven transporters is also interesting. It could be related to the higher affinities of ATP transporters that allowed the cells to capture rare substrates more efficiently. It is also possible that ATP transporters have diversified from the early ATP synthases and this partly explains why they are therefore so abundant in bacteria and archaea.

All of the eight prokarya-specific secondary transporter families (Additional file 2: Data S1) were energy-dependent, i.e., dependent on a proton or sodium gradient, or utilizing a combination of electro- and chemical potential of the membrane [46–51]. A surprising insight here was related to the six evolutionarily younger secondary transporter families found only in eukaryotes (See Additional file 2: Data S1): four of these, about which we have experimental information, were energy-independent and low-energy-demanding transporter families for bile acid, choline, silicate, and vitamin A. The last two belong to the 4 TMS multidrug endosomal transporter and chloroplast maltose exporter families. The choline transporter-like family is involved in choline influx [52]. The ‘birth’ of a cheap and sodium-independent transporter for choline is important because of the broad cellular usage of choline. Choline is an essential precursor for membranes and for the neuromodulator acetylcholine [53, 54]. Another recently evolved transport family was the organic solute transporter family, which is involed in the facilitated diffusion of bile acids from enterocytes into the blood [55]. The silicon transporter family is also energetically cheap and has a silicate:sodium symport stoichiometry of 1:1 [56]. Finally, the animal-specific vitamin A receptor/transporter (STRA6) mediates costless influx and efflux of vitamin A derivatives by a mechanism not seen in any other transporter class [57, 58]. Of particular interest, the animal visual system is dependent on vitamin A and has a substantial energetic cost, e.g., up to 15% of resting metabolism in the Mexican fish *Astyanax mexicanus* [59, 60]. Such photo-detection involves the single-photon-triggered isomerization of 11-*cis*-retinal to all-*trans*-retinal, which must be recycled back through efflux and influx steps of these isomers between the retinal pigmented epithelial and photoreceptor cells [57, 61–63]. Thus, the evolved energy-independent membrane translocation of the vitamin A isomers seems to be an adaptive trait for higher energetic performance. This is in line with the positive selection reported for STRA6 in different mammalian phyla [64]. Additionally, we found a higher representation of ion channels in the animal kingdom when compared to the other domains of life (Fig. 2d). The membrane transport of ions is important for highly energy-demanding sensory tissues [8, 65] and it can therefore be hypothesized that the extensive diversification of ion channels and the costless transport of vitamin A in animals are trade-offs between the benefits of the evolved nervous and visual systems and their high energy requirements. Overall, our findings demonstrate a clear and unequivocal change in the energetic efficiency of transportomes during the course of evolution, a very significant finding.

## Conclusions

The present inter-kingdom comparison of transportomes provides genome-scale molecular evidence for their evolution towards an improved energetic efficiency. This has likely been very influential due to the high energy demand of the cellular transport machinery and also for the development of tissues performing energetically costly functions. The present data also strengthen members of the previously reported bacterial order Rickettsiales as the origin of the mitochondrion by recognising *Neorickettsia risticii* and *Neorickettsia sennetsu* as the sole species in this order whose genomes harbor putative mitochondria-specific solute carrier (SLC25) coding genes. Since other evidence had also suggested Rickettsiales as candidates for this, our transoportome findings strengthen considerably the case for such a lineage.

## Methods

The publicly-available membrane transporter data on ion channels and secondary transporters were extracted from TransportDB (http://www.membranetransport.org/transportDB2/) [23] . The transportomes of 126 prokaryotic species (78 bacteria and 48 archaea) and 96 eukaryotic species (22 primitive eukaryotes, 24 algae and plants, 23 fungi, and 27 animals) (See Additional file 2: Data S1) were annotated using the Transporter Automatic Annotation Pipeline at TransportDB [23]. We also included 27 eukaryotic species including the transportomes of 8 primitive eukaryotes, 6 algae and plants, 7 fungi, and 6 animals publicly available at TransportDB (See Additional file 2: Data S1). To study the compositional changes of transportomes, we did not include any transportomes from prokaryotes publicly available at the TransportDB. This is due to the incomplete information on the ABC transporters. The majority of ABC transporters in prokaryotes are coded by different genes of an operon, where each gene codes for different subunits [66], and these should be excluded from the data and considered as single transporters in our analyses on the transportome composition. However, this information on the ABC coding genes and operons is not provided for the publicly-available transportomes of prokaryotes.

To predict the transportomes, the proteomes of organisms were downloaded from the Genbank and Ensembl databases (http://www.ensembl.org/index.html, http://fungi.ensembl.org/index.html, http://protists.ensembl.org/index.html, http://plants.ensembl.org/index.html) [67]. All proteins with fewer than 100 amino acids were excluded. Taken together, we analysed 78 bacterial, 48 archaeal, 30 primitive eukaryotes, 30 algal and plant, 30 fungal, and 33 animal transportomes, each representing one independent biological replicate (per species). The list of organisms with their genome size and total number of transporters is shown in Additional file 2: Data S1 and Additional file 1: Table S1. The annotated transportomes were manually filtered for multi-prediction hits as well as the alternative isoforms of transport proteins before the analysis. Taken together, our analyses on the transporter families included all of the 15 ATP-dependent transporter families, 51 ion channel families, and 90 secondary transporter families which were present in the studied organisms. We used Student’s t-test to examine the possible differences among the samples, i.e., domains of life. The data were in the format of counts and percentages. Therefore, to exclude a possible divergence from normality, we also performed the analyses on the arc sin √x transformed data. All the analyses can be found in Additional file 2: Data S1.

To make an approximation of the energetic performance of the cellular transportomes, we calculated the inter-transportome changes in energy-usage efficiency (EUE), which we defined as the average free energy demand for a single transport event. The following assumptions were made when calculating EUE values. Equilibrative ion channels require no free energy for the transport action. While some of the secondary transporters also act in an energy-independent manner known as equilibrative transport or facilitated diffusion, others exploit the electrochemical potential established by ATPases across membranes [14, 22, 68–70]. For active transport, secondary transporters are considered to use membrane electrochemical potential gradients, coupled to a varying stoichiometry (0.5 to 3) of ions per turnover [69, 71–92]. Considering the stoichiometry of ≈ 2–3 ions pumped per ATP hydrolysed by ATPases [93–102], even concentrative secondary transporters would not use more than 0.5 ATP equivalent per substrate translocation across a membrane on average. The average of two substrates per ATP tends, therefore, to be conservative for secondary transporters and a higher rate of substrate translocation can also be expected. In contrast, the ATP-dependent members belonging to the ABC superfamily, and also the mitochondrial protein translocase, the type III secretory pathway, the chloroplast envelope protein translocase, and the arsenite-antimonite efflux families, generally show a 1:2 stoichiometry of substrate:ATP hydrolysis [103–109]. To calculate the inter-transportome variations in the energy-usage efficiency (ΔEUE), we therefore applied the equation:

$$ \Delta \mathrm{EUE}\ \left(\Delta \mathrm{ATP}-\mathrm{usage}\ \mathrm{per}\ \mathrm{single}\ \mathrm{transport}\ \mathrm{cycle}\right)=\left[{2}_{\mathrm{ATP}}\times \Delta \%\mathrm{ADT}+{0.5}_{\mathrm{ATP}}\times \Delta \%\mathrm{ST}+{0.0}_{\mathrm{ATP}}\times \Delta \%\mathrm{IC}\right]/100, $$

where ADT, ST, and IC are ATP-dependent transporters, secondary transporters, and ion-channels, respectively. The F/V/A-type ATPases and ATP synthases were excluded from the ATP-dependent transporters. This is because they provide the energy as ATP or membrane electrochemical potential for the rest of the transporters. It is worthy of note that the efficiency of energy usage indicates the average energy demand per unit of action. So the expression and activity levels of transporters are not taken into the account when calculating the EUE. A more general example would compare two organisms with exactly the same transportome but with differences in the expression levels of transportome members among them. Here, the EUE of transportome machineries of the two organisms are equal since they use exactly same transportome machinery. This is thus irrespective of the differences in activity levels of transporters, which define the total energy demand for the given transportome.

## Additional files

Additional file 1: **Table S1.** The list of organisms with publicly-available data on the ion channels and secondary transporters. (DOCX 168 kb)

Additional file 2: **Data S1.** Genomic and transportome data on the organisms included in the study. (XLSX 255 kb)

## Abbreviations

ADT: ATP-dependent transporters
EUE: energy-usage efficiency
IC: ion-channels
ST: secondary transporters
