# Supplementary material for: Energetic evolution of cellular Transportomes
Source: BMC Genomics. 2018 May 30;19:418. doi: 10.1186/s12864-018-4816-5 (PMC5977736; doi:10.1186/s12864-018-4816-5)
Supplement: Supplementary file 1 — Table S1. The list of organisms with publicly-available data on the ion channels and secondary transporters. (DOCX 168 kb) [file 12864_2018_4816_MOESM1_ESM.docx]

Supplementary Table 1. The list of species with publicly-available data on the ion channels and secondary transporters.

| **Domain of life** | **Genus** | **Species** | **Genome size (Mb)** | **Total number of transporter proteins** | **Transportome source** |
| --- | --- | --- | --- | --- | --- |
| Stramenopile, diatom | *Phaeodactylum* | *tricornutum* CCAP1055/1 | 27.6 | 518 | TransportDB |
| Stramenopile, diatom | *Thalassiosira* | *pseudonana* CCMP1335 | 32.4 | 476 | TransportDB |
| Archaea | *Aciduliprofundum* | *boonei* T469 | 1.49 | 151 | TransportDB |
| Archaea | *Aeropyrum* | *camini* SY1JCM12091 | 1.6 | 165 | TransportDB |
| Archaea | *Archaeoglobus* | *fulgidus* DSM4304 | 2.18 | 203 | TransportDB |
| Archaea | *Archaea* | *halophilic* DL31 | 3.64 | 316 | TransportDB |
| Archaea | *Acidianus* | *hospitalis* W1 | 2.14 | 176 | TransportDB |
| Archaea | *Aeropyrum* | *pernix* K1 | 1.67 | 169 | TransportDB |
| Archaea | *Archaeoglobus* | *profundus* DSM5631 | 1.56 | 127 | TransportDB |
| Archaea | *Acidilobus* | *saccharovorans* 34515 | 1.50 | 142 | TransportDB |
| Archaea | *Aciduliprofundum* | *sMAR08339* | 1.44 | 144 | TransportDB |
| Archaea | *Archaeoglobus* | *sulfaticallidus* PM701 | 2.08 | 168 | TransportDB |
| Archaea | *Archaeoglobus* | *veneficus* SNP6 | 1.9 | 161 | TransportDB |
| Archaea | *Candidatus* | *caldiarchaeum* subterraneum | 1.68 | 177 | TransportDB |
| Archaea | *Candidatus* | *korarchaeum cryptofilum* OPF8 | 1.59 | 153 | TransportDB |
| Archaea | *Caldisphaera* | *lagunensis* DSM15908 | 1.55 | 133 | TransportDB |
| Archaea | *Caldivirga* | *maquilingensis* IC167 | 2.08 | 176 | TransportDB |
| Archaea | *Candidatus* | *methanoregula boonei* 6A8 | 2.54 | 200 | TransportDB |
| Archaea | *Candidatus* | *nitrosopumilus koreensis* AR1 | 1.64 | 75 | TransportDB |
| Archaea | *Candidatus* | *nitrosopumilus* spAR2 | 1.69 | 84 | TransportDB |
| Archaea | *Candidatus* | *nitrososphaera gargensis* Ga92 | 2.83 | 140 | TransportDB |
| Archaea | *Cenarchaeum* | *symbiosum* A | 2.05 | 65 | TransportDB |
| Archaea | *Desulfurococcus* | *fermentans* DSM16532 | 1.38 | 157 | TransportDB |
| Archaea | *Desulfurococcus* | *kamchatkensis* 1221n | 1.37 | 156 | TransportDB |
| Archaea | *Desulfurococcus* | *mucosus* DSM2162 | 1.31 | 159 | TransportDB |
| Archaea | *Ferroplasma* | *acidarmanus* fer1 | 1.94 | 183 | TransportDB |
| Archaea | *Fervidicoccus* | *fontis* Kam940 | 1.32 | 111 | TransportDB |
| Archaea | *Ferroglobus* | *placidus* DSM10642 | 2.2 | 180 | TransportDB |
| Archaea | *Halogeometricum* | *borinquense* DSM11551 | 3.94 | 356 | TransportDB |
| Archaea | *Hyperthermus* | *butylicus* DSM5456 | 1.67 | 137 | TransportDB |
| Archaea | *Haloarcula* | *hispanica* ATCC33960 | 3.89 | 376 | TransportDB |
| Archaea | *Haloarcula* | *hispanica* N601 | 3.9 | 375 | TransportDB |
| Archaea | *Halalkalicoccus* | *jeotgali* B3 | 3.7 | 349 | TransportDB |
| Archaea | *Halorubrum* | *lacusprofundi* ATCC49239 | 3.69 | 301 | TransportDB |
| Archaea | *Haloarcula* | *marismortui* ATCC43049 | 4.27 | 378 | TransportDB |
| Archaea | *Haloferax* | *mediterranei* ATCC33500 | 3.9 | 370 | TransportDB |
| Archaea | *Halomicrobium* | *mukohataei* DSM12286 | 3.33 | 268 | TransportDB |
| Archaea | *Halovivax* | *ruber* XH70 | 3.22 | 263 | TransportDB |
| Archaea | *Halobacterium* | *salinarum* R1 | 2.67 | 214 | TransportDB |
| Archaea | *Halobacterium* | *sNRC1* | 2.57 | 201 | TransportDB |
| Archaea | *Halorhabdus* | *tiamatea* SARL4B | 3.15 | 216 | TransportDB |
| Archaea | *Haloterrigena* | *turkmenica* DSM5511 | 5.44 | 465 | TransportDB |
| Archaea | *Halorhabdus* | *utahensis* DSM12940 | 3.12 | 217 | TransportDB |
| Archaea | *Haloferax* | *volcanii* DS2 | 4.01 | 423 | TransportDB |
| Archaea | *Haloquadratum* | *walsbyi* C23 | 3.26 | 259 | TransportDB |
| Archaea | *Haloquadratum* | *walsbyi* DSM16790 | 3.18 | 268 | TransportDB |
| Archaea | *Halopiger* | *xanaduensis* SH6 | 4.36 | 399 | TransportDB |
| Archaea | *Ignisphaera* | *aggregans* DSM17230 | 1.88 | 175 | TransportDB |
| Archaea | *Ignicoccus* | *hospitalis* KIN4I | 1.3 | 78 | TransportDB |
| Archaea | *Methanosarcina* | *acetivorans* C2A | 5.75 | 449 | TransportDB |
| Archaea | *Methanococcus* | *aeolicus* Nankai3 | 1.57 | 109 | TransportDB |
| Archaea | *Methanocella* | *arvoryzae* MRE50 | 3.18 | 239 | TransportDB |
| Archaea | *Methanosarcina* | *barkeri* str Fusaro | 4.87 | 356 | TransportDB |
| Archaea | *Methanoregula* | *boonei* 6A8 | 2.54 | 186 | TransportDB |
| Archaea | *Methanoculleus* | *bourgensis* MS2 | 2.79 | 224 | TransportDB |
| Archaea | *Methanococcoides* | *burtonii* DSM6242 | 2.58 | 187 | TransportDB |
| Archaea | *Methanosaeta* | *concilii* GP6 | 3.03 | 234 | TransportDB |
| Archaea | *Methanocella* | *conradii* HZ254 | 2.38 | 241 | TransportDB |
| Archaea | *Metallosphaera* | *cuprina* Ar4 | 1.84 | 150 | TransportDB |
| Archaea | *Methanohalobium* | *evestigatum* Z7303 | 2.41 | 161 | TransportDB |
| Archaea | *Methanocaldococcus* | *fervens* AG86 | 1.51 | 107 | TransportDB |
| Archaea | *Methanothermus* | *fervidus* DSM2088 | 1.24 | 77 | TransportDB |
| Archaea | *Methanoregula* | *formicicum* SMSP | 2.82 | 216 | TransportDB |
| Archaea | *Methanosaeta* | *harundinacea* 6Ac | 2.57 | 190 | TransportDB |
| Archaea | *Methanomethylovorans* | *hollandica* DSM15978 | 2.71 | 199 | TransportDB |
| Archaea | *Methanospirillum* | *hungatei* JF1 | 3.54 | 286 | TransportDB |
| Archaea | *Methanotorris* | *igneus* Kol5 | 1.85 | 135 | TransportDB |
| Archaea | *Methanocaldococcus* | *infernus* ME | 1.33 | 116 | TransportDB |
| Archaea | *Methanopyrus* | *kandleri* AV19 | 1.69 | 67 | TransportDB |
| Archaea | *Methanocorpusculum* | *labreanum* Z | 1.8 | 181 | TransportDB |
| Archaea | *Methanohalophilus* | *mahii* DSM5219 | 2.01 | 171 | TransportDB |
| Archaea | *Methanothermobacter* | *marburgensis* str Marburg | 1.64 | 113 | TransportDB |
| Archaea | *Methanococcus* | *maripaludis* C5 | 1.79 | 156 | TransportDB |
| Archaea | *Methanococcus* | *maripaludis* C6 | 1.74 | 157 | TransportDB |
| Archaea | *Methanococcus* | *maripaludis* C7 | 1.77 | 166 | TransportDB |
| Archaea | *Methanococcus* | *maripaludis* S2 | 1.66 | 167 | TransportDB |
| Archaea | *Methanococcus* | *maripaludis* X1 | 1.75 | 169 | TransportDB |
| Archaea | *Methanoculleus* | *marisnigri* JR1 | 2.48 | 208 | TransportDB |
| Archaea | *Methanosarcina* | *mazei* Go1 | 4.1 | 286 | TransportDB |
| Archaea | *Methanosarcina* | *mazei* Tuc01 | 3.43 | 248 | TransportDB |
| Archaea | *Methanothermococcus* | *okinawensis* IH1 | 1.68 | 105 | TransportDB |
| Archaea | *Methanocella* | *paludicola* SANAE | 2.96 | 267 | TransportDB |
| Archaea | *Methanosphaerula* | *palustris* E19c | 2.92 | 223 | TransportDB |
| Archaea | *Methanoplanus* | *petrolearius* DSM11571 | 2.84 | 282 | TransportDB |
| Archaea | *Methanolobus* | *psychrophilus* R15 | 3.07 | 216 | TransportDB |
| Archaea | *Methanobrevibacter* | *ruminantium* M1 | 2.94 | 151 | TransportDB |
| Archaea | *Metallosphaera* | *sedula* DSM5348 | 2.19 | 176 | TransportDB |
| Archaea | *Methanobrevibacter* | *smithii* ATCC35061 | 1.85 | 118 | TransportDB |
| Archaea | *Methanobrevibacter* | *sAbM4* | 2.0 | 124 | TransportDB |
| Archaea | *Methanobacterium* | *sAL21* | 2.58 | 197 | TransportDB |
| Archaea | *Methanocaldococcus* | *sFS40622* | 1.77 | 126 | TransportDB |
| Archaea | *Methanobacterium* | *sMB1* | 2.03 | 145 | TransportDB |
| Archaea | *Methanomassiliicoccus* | *sMx1Issoire* | 1.93 | 173 | TransportDB |
| Archaea | *Methanocella* | *sRC-I* | 3.18 | 185 | TransportDB |
| Archaea | *Methanobacterium* | *sSWAN1* | 2.55 | 184 | TransportDB |
| Archaea | *Methanothermobacter* | *thermautotrophicus* str DeltaH | 1.75 | 117 | TransportDB |
| Archaea | *Methanosaeta* | *thermophila* PT | 1.88 | 146 | TransportDB |
| Archaea | *Methanococcus* | *vannielii* SB | 1.72 | 138 | TransportDB |
| Archaea | *Methanococcus* | *voltae* A3 | 1.94 | 134 | TransportDB |
| Archaea | *Methanocaldococcus* | *vulcanius* M7 | 1.76 | 105 | TransportDB |
| Archaea | *Methanosalsum* | *zhilinae* DSM4017 | 2.14 | 162 | TransportDB |
| Archaea | *Nanoarchaeum* | *equitans* Kin4M | 0.49 | 22 | TransportDB |
| Archaea | *Natronobacterium* | *gregoryi* SP2 | 3.79 | 309 | TransportDB |
| Archaea | *Natrialba* | *magadii* ATCC43099 | 4.44 | 351 | TransportDB |
| Archaea | *Nitrosopumilus* | *maritimus* SCM1 | 1.65 | 94 | TransportDB |
| Archaea | *Natronomonas* | *moolapensis* 8811 | 2.91 | 214 | TransportDB |
| Archaea | *Natronococcus* | *occultus* SP4 | 4.31 | 376 | TransportDB |
| Archaea | *Natrinema* | *pellirubrum* DSM15624 | 4.35 | 335 | TransportDB |
| Archaea | *Natronomonas* | *pharaonis* DSM2160 | 2.75 | 240 | TransportDB |
| Archaea | *Natrinema* | *sJ72* | 3.79 | 297 | TransportDB |
| Archaea | *Pyrococcus* | *abyssi* GE5 | 1.77 | 199 | TransportDB |
| Archaea | *Pyrobaculum* | *aerophilum* str IM2 | 2.22 | 191 | TransportDB |
| Archaea | *Pyrobaculum* | *arsenaticum* DSM13514 | 2.12 | 171 | TransportDB |
| Archaea | *Pyrobaculum* | *calidifontis* JCM11548 | 2.01 | 169 | TransportDB |
| Archaea | *Pyrolobus* | *fumarii* 1A | 1.84 | 117 | TransportDB |
| Archaea | *Pyrococcus* | *furiosus* COM1 | 1.91 | 222 | TransportDB |
| Archaea | *Pyrococcus* | *furiosus* DSM3638 | 1.91 | 224 | TransportDB |
| Archaea | *Pyrococcus* | *horikoshii* OT3 | 1.74 | 191 | TransportDB |
| Archaea | *Pyrococcus* | *horikoshii* (shinkaj) OT3 | 1.74 | 201 | TransportDB |
| Archaea | *Pyrobaculum* | *islandicum* DSM4184 | 1.83 | 168 | TransportDB |
| Archaea | *Pyrobaculum* | *neutrophilum* V24Sta | 1.77 | 159 | TransportDB |
| Archaea | *Pyrobaculum* | *oguniense* TE7 | 2.45 | 173 | TransportDB |
| Archaea | *Pyrobaculum* | *s1860* | 2.47 | 186 | TransportDB |
| Archaea | *Pyrococcus* | *sNA2* | 1.86 | 204 | TransportDB |
| Archaea | *Pyrococcus* | *sST04* | 1.74 | 193 | TransportDB |
| Archaea | *Picrophilus* | *torridus* DSM9790 | 1.55 | 188 | TransportDB |
| Archaea | *Pyrococcus* | *yayanosii* CH1 | 1.72 | 187 | TransportDB |
| Archaea | *Sulfolobus* | *acidocaldarius* DSM639 | 2.23 | 161 | TransportDB |
| Archaea | *Sulfolobus* | *acidocaldarius* N8 | 2.18 | 155 | TransportDB |
| Archaea | *Sulfolobus* | *acidocaldarius* Ron12I | 2.22 | 160 | TransportDB |
| Archaea | *Sulfolobus* | *acidocaldarius* SUSAZ | 2.06 | 158 | TransportDB |
| Archaea | *Staphylothermus* | *hellenicus* DSM12710 | 1.58 | 140 | TransportDB |
| Archaea | *Sulfolobus* | *islandicus* HVE104 | 2.66 | 226 | TransportDB |
| Archaea | *Sulfolobus* | *islandicus* LAL141 | 2.47 | 233 | TransportDB |
| Archaea | *Sulfolobus* | *islandicus* LD85 | 2.75 | 223 | TransportDB |
| Archaea | *Sulfolobus* | *islandicus* LS215 | 2.74 | 215 | TransportDB |
| Archaea | *Sulfolobus* | *islandicus* M1425 | 2.61 | 222 | TransportDB |
| Archaea | *Sulfolobus* | *islandicus* M1627 | 2.69 | 225 | TransportDB |
| Archaea | *Sulfolobus* | *islandicus* M164 | 2.59 | 220 | TransportDB |
| Archaea | *Sulfolobus* | *islandicus* REY15A | 2.52 | 215 | TransportDB |
| Archaea | *Sulfolobus* | *islandicus* YG5714 | 2.70 | 218 | TransportDB |
| Archaea | *Sulfolobus* | *islandicus* YN1551 | 2.85 | 208 | TransportDB |
| Archaea | *Staphylothermus* | *marinus* F1 | 1.57 | 166 | TransportDB |
| Archaea | *Sulfolobus* | *solfataricus* 982 | 2.67 | 206 | TransportDB |
| Archaea | *Sulfolobus* | *solfataricus* P2 | 2.99 | 244 | TransportDB |
| Archaea | *Salinarchaeum* | *sHarcht* Bsk1 | 3.26 | 241 | TransportDB |
| Archaea | *Sulfolobus* | *tokodaii* str 7 | 2.69 | 188 | TransportDB |
| Archaea | *Thermoplasma* | *acidophilum* DSM1728 | 1.56 | 169 | TransportDB |
| Archaea | *Thermosphaera* | *aggregans* DSM11486 | 1.32 | 148 | TransportDB |
| Archaea | *Thermoplasmatales* | *archaeon* BRNA1 | 1.46 | 116 | TransportDB |
| Archaea | *Thermococcus* | *barophilus* MP | 2.06 | 235 | TransportDB |
| Archaea | *Thermogladius* | *cellulolyticus* 1633 | 1.36 | 166 | TransportDB |
| Archaea | *Thermococcus* | *gammatolerans* EJ3 | 2.05 | 217 | TransportDB |
| Archaea | *Thermococcus* | *kodakarensis* KOD1 | 2.09 | 222 | TransportDB |
| Archaea | *Thermococcus* | *litoralis* DSM5473 | 2.22 | 279 | TransportDB |
| Archaea | *Thermoproteus* | *neutrophilus* V24Sta | 1.77 | 163 | TransportDB |
| Archaea | *Thermococcus* | *onnurineus* NA1 | 1.85 | 217 | TransportDB |
| Archaea | *Thermofilum* | *pendens* Hrk5 | 1.81 | 208 | TransportDB |
| Archaea | *Thermococcus* | *sibiricus* MM739 | 1.85 | 219 | TransportDB |
| Archaea | *Thermofilum* | *s1910b* | 1.75 | 208 | TransportDB |
| Archaea | *Thermococcus* | *s4557* | 2.01 | 233 | TransportDB |
| Archaea | *Thermococcus* | *sAM4* | 2.09 | 212 | TransportDB |
| Archaea | *Thermococcus* | *sCL1* | 1.95 | 192 | TransportDB |
| Archaea | *Thermoproteus* | *tenax* Kra1 | 1.84 | 163 | TransportDB |
| Archaea | *Thermoproteus* | *uzoniensis* 76820 | 1.94 | 169 | TransportDB |
| Archaea | *Thermoplasma* | *volcanium* GSS1 | 1.58 | 151 | TransportDB |
| Archaea | *Vulcanisaeta* | *distributa* DSM14429 | 2.37 | 169 | TransportDB |
| Archaea | *Vulcanisaeta* | *moutnovskia* 76828 | 2.3 | 173 | TransportDB |
| Bacteria | *Alicyclobacillus* | *acidocaldarius* subsp acidocaldarius DSM446 | 3.21 | 309 | TransportDB |
| Bacteria | *Alicyclobacillus* | *acidocaldarius* subsp acidocaldarius Tc41 | 3.12 | 306 | TransportDB |
| Bacteria | *Aggregatibacter* | *actinomycetemcomitans* ANH9381 | 2.14 | 283 | TransportDB |
| Bacteria | *Aggregatibacter* | *actinomycetemcomitans* D11S1 | 2.20 | 290 | TransportDB |
| Bacteria | *Aggregatibacter* | *actinomycetemcomitans* D7S1 | 2.31 | 287 | TransportDB |
| Bacteria | *Aquifex* | *aeolicus* VF5 | 1.59 | 115 | TransportDB |
| Bacteria | *Aggregatibacter* | *aphrophilus* NJ8700 | 2.31 | 276 | TransportDB |
| Bacteria | *Acetohalobium* | *arabaticum* DSM5501 | 2.47 | 231 | TransportDB |
| Bacteria | *Arthrobacter* | *arilaitensis* Re117 | 3.92 | 399 | TransportDB |
| Bacteria | *Aromatoleum* | *aromaticum* EbN1 | 4.73 | 330 | TransportDB |
| Bacteria | *Arthrobacter* | *aurescens* TC1 | 5.23 | 590 | TransportDB |
| Bacteria | *Acidovorax* | *avenae citrulli* AAC00-1 | 5.35 | 534 | TransportDB |
| Bacteria | *Acidovorax* | *avenae* subsp avenae ATCC19860 | 5.48 | 636 | TransportDB |
| Bacteria | *Acinetobacter* | *baumannii* 16562 | 4.02 | 400 | TransportDB |
| Bacteria | *Acinetobacter* | *baumannii* AB0057 | 4.06 | 390 | TransportDB |
| Bacteria | *Acinetobacter* | *baumannii* AB3070294 | 3.76 | 385 | TransportDB |
| Bacteria | *Acinetobacter* | *baumannii* ACICU | 4.0 | 404 | TransportDB |
| Bacteria | *Acinetobacter* | *baumannii* ATCC17978 | 4.0 | 409 | TransportDB |
| Bacteria | *Acinetobacter* | *baumannii* AYE | 4.05 | 398 | TransportDB |
| Bacteria | *Acinetobacter* | *baumannii* BJAB07104 | 4.04 | 407 | TransportDB |
| Bacteria | *Acinetobacter* | *baumannii* BJAB0715 | 4.05 | 398 | TransportDB |
| Bacteria | *Acinetobacter* | *baumannii* BJAB0868 | 4.01 | 406 | TransportDB |
| Bacteria | *Acinetobacter* | *baumannii* D1279779 | 3.71 | 372 | TransportDB |
| Bacteria | *Acinetobacter* | *baumannii* MDRTJ | 4.15 | 406 | TransportDB |
| Bacteria | *Acinetobacter* | *baumannii* MDRZJ06 | 4.01 | 401 | TransportDB |
| Bacteria | *Acinetobacter* | *baumannii* SDF | 3.48 | 242 | TransportDB |
| Bacteria | *Acinetobacter* | *baumannii* TCDCAB0715 | 4.22 | 406 | TransportDB |
| Bacteria | *Acinetobacter* | *baumannii* TYTH1 | 3.96 | 396 | TransportDB |
| Bacteria | *Acinetobacter* | *baumannii* ZW851 | 3.88 | 402 | TransportDB |
| Bacteria | *Alcanivorax* | *borkumensis* SK2 | 3.12 | 235 | TransportDB |
| Bacteria | *Azospirillum* | *brasilense* Sp245 | 7.53 | 877 | TransportDB |
| Bacteria | *Acholeplasma* | *brassicae* | 1.88 | 236 | TransportDB |
| Bacteria | *Arcobacter* | *butzleri* 7h1h | 2.25 | 217 | TransportDB |
| Bacteria | *Arcobacter* | *butzleri* ED1 | 2.26 | 206 | TransportDB |
| Bacteria | *Arcobacter* | *butzleri* RM4018 | 2.34 | 218 | TransportDB |
| Bacteria | *Acinetobacter* | *calcoaceticus* ADP1 | 3.6 | 355 | TransportDB |
| Bacteria | *Acinetobacter* | *calcoaceticus* PHEA2 | 3.86 | 427 | TransportDB |
| Bacteria | *Acidithiobacillus* | *caldus* SM1 | 3.24 | 231 | TransportDB |
| Bacteria | *Acidobacterium* | *capsulatum* ATCC51196 | 4.13 | 255 | TransportDB |
| Bacteria | *Azorhizobium* | *caulinodans* ORS571 | 5.37 | 801 | TransportDB |
| Bacteria | *Acidothermus* | *cellulolyticus* 11B | 2.44 | 209 | TransportDB |
| Bacteria | *Anaplasma* | *centrale* str Israel | 1.21 | 78 | TransportDB |
| Bacteria | *Arthrobacter* | *chlorophenolicus* A6 | 4.98 | 527 | TransportDB |
| Bacteria | *Acidovorax* | *citrulli* AAC001 | 5.35 | 526 | TransportDB |
| Bacteria | *Aminobacterium* | *colombiense* DSM12261 | 1.98 | 292 | TransportDB |
| Bacteria | *Acidiphilium* | *cryptum* JF5 | 3.96 | 407 | TransportDB |
| Bacteria | *Anabaena* | *cylindrica* PCC7122 | 7.06 | 386 | TransportDB |
| Bacteria | *Ammonifex* | *degensii* KC4 | 2.16 | 145 | TransportDB |
| Bacteria | *Anaeromyxobacter* | *dehalogenans* 2CP1 | 5.03 | 335 | TransportDB |
| Bacteria | *Anaeromyxobacter* | *dehalogenans* 2CPC | 5.01 | 332 | TransportDB |
| Bacteria | *Alicycliphilus* | *denitrificans* BC | 4.84 | 588 | TransportDB |
| Bacteria | *Alicycliphilus* | *denitrificans* K601 | 5.07 | 627 | TransportDB |
| Bacteria | *Alcanivorax* | *dieselolei* B5 | 4.93 | 476 | TransportDB |
| Bacteria | *Acidovorax* | *ebreus* TPSY | 3.80 | 454 | TransportDB |
| Bacteria | *Alkalilimnicola* | *ehrlichii* MLHE1 | 3.28 | 279 | TransportDB |
| Bacteria | *Adlercreutzia* | *equolifaciens* DSM19450 | 2.86 | 234 | TransportDB |
| Bacteria | *Asticcacaulis* | *excentricus* CB48 | 4.31 | 268 | TransportDB |
| Bacteria | *Acidaminococcus* | *fermentans* DSM20731 | 2.33 | 268 | TransportDB |
| Bacteria | *Acidithiobacillus* | *ferrivorans* SS3 | 3.21 | 218 | TransportDB |
| Bacteria | *Acidithiobacillus* | *ferrooxidans* ATCC23270 | 2.98 | 226 | TransportDB |
| Bacteria | *Acidithiobacillus* | *ferrooxidans* ATCC53993 | 2.89 | 228 | TransportDB |
| Bacteria | *Acidimicrobium* | *ferrooxidans* DSM10331 | 2.16 | 172 | TransportDB |
| Bacteria | *Alistipes* | *finegoldii* DSM17242 | 3.73 | 161 | TransportDB |
| Bacteria | *Anoxybacillus* | *flavithermus* WK1 | 2.85 | 299 | TransportDB |
| Bacteria | *Actinoplanes* | *friuliensis* DSM7358 | 9.38 | 817 | TransportDB |
| Bacteria | *Arcanobacterium* | *haemolyticum* DSM20595 | 1.99 | 246 | TransportDB |
| Bacteria | *Aeromonas* | *hydrophila* ML09119 | 5.02 | 565 | TransportDB |
| Bacteria | *Aeromonas* | *hydrophila* subsp hydrophila ATCC7966 | 4.74 | 554 | TransportDB |
| Bacteria | *Acidaminococcus* | *intestini* RyCMR95 | 2.49 | 273 | TransportDB |
| Bacteria | *Advenella* | *kashmirensis* WT001 | 4.42 | 625 | TransportDB |
| Bacteria | *Acholeplasma* | *laidlawii* PG8A | 1.5 | 172 | TransportDB |
| Bacteria | *Azospirillum* | *lipoferum* 4B | 6.85 | 751 | TransportDB |
| Bacteria | *Alteromonas* | *macleodii* AltDE1 | 4.95 | 313 | TransportDB |
| Bacteria | *Alteromonas* | *macleodii* ATCC27126 | 4.65 | 304 | TransportDB |
| Bacteria | *Alteromonas* | *macleodii* str Aegean Sea MED64 | 4.4 | 300 | TransportDB |
| Bacteria | *Alteromonas* | *macleodii* str Balearic Sea AD45 | 4.67 | 298 | TransportDB |
| Bacteria | *Alteromonas* | *macleodii* str Black Sea11 | 4.48 | 301 | TransportDB |
| Bacteria | *Alteromonas* | *macleodii* str Deepecotype | 4.45 | 298 | TransportDB |
| Bacteria | *Alteromonas* | *macleodii* str English Channel 615 | 4.58 | 245 | TransportDB |
| Bacteria | *Alteromonas* | *macleodii* str English Channel673 | 4.6 | 314 | TransportDB |
| Bacteria | *Alteromonas* | *macleodii* str Ionian SeaU4 | 4.62 | 309 | TransportDB |
| Bacteria | *Alteromonas* | *macleodii* str Ionian SeaU7 | 4.44 | 293 | TransportDB |
| Bacteria | *Alteromonas* | *macleodii* str Ionian SeaU8 | 4.4 | 300 | TransportDB |
| Bacteria | *Alteromonas* | *macleodii* str Ionian SeaUM4b | 4.44 | 302 | TransportDB |
| Bacteria | *Alteromonas* | *macleodii* str Ionian SeaUM7 | 4.93 | 309 | TransportDB |
| Bacteria | *Anaplasma* | *marginale* str Dawn | 1.2 | 60 | TransportDB |
| Bacteria | *Anaplasma* | *marginale* str Florida | 1.2 | 77 | TransportDB |
| Bacteria | *Anaplasma* | *marginale* str Gypsy Plains | 1.2 | 65 | TransportDB |
| Bacteria | *Anaplasma* | *marginale* str StMaries | 1.2 | 77 | TransportDB |
| Bacteria | *Acaryochloris* | *marina* MBIC11017 | 8.36 | 416 | TransportDB |
| Bacteria | *Amycolatopsis* | *mediterranei* RB | 10.25 | 799 | TransportDB |
| Bacteria | *Amycolatopsis* | *mediterranei* S699 | 10.25 | 802 | TransportDB |
| Bacteria | *Amycolatopsis* | *mediterranei* U32 | 10.24 | 800 | TransportDB |
| Bacteria | *Alkaliphilus* | *metalliredigens* QYMF | 4.93 | 545 | TransportDB |
| Bacteria | *Actinosynnema* | *mirum* DSM43827 | 8.25 | 633 | TransportDB |
| Bacteria | *Actinoplanes* | *missouriensis* 431 | 8.77 | 741 | TransportDB |
| Bacteria | *Anaerobaculum* | *mobile* DSM13181 | 2.16 | 296 | TransportDB |
| Bacteria | *Akkermansia* | *muciniphila* ATCCBAA835 | 2.66 | 164 | TransportDB |
| Bacteria | *Acidiphilium* | *multivorum* AIU301 | 4.21 | 420 | TransportDB |
| Bacteria | *Arcobacter* | *nitrofigilis* DSM7299 | 3.19 | 385 | TransportDB |
| Bacteria | *Acinetobacter* | *oleivorans* DR1 | 4.15 | 436 | TransportDB |
| Bacteria | *Alkaliphilus* | *oremlandii* OhILAs | 3.12 | 359 | TransportDB |
| Bacteria | *Amycolatopsis* | *orientalis* HCCB10007 | 8.95 | 760 | TransportDB |
| Bacteria | *Acholeplasma* | *palmae* J233 | 1.55 | 180 | TransportDB |
| Bacteria | *Atopobium* | *parvulum* DSM20469 | 1.54 | 200 | TransportDB |
| Bacteria | *Acetobacter* | *pasteurianus* 386B | 3.06 | 266 | TransportDB |
| Bacteria | *Acetobacter* | *pasteurianus* IFO328301 | 3.34 | 284 | TransportDB |
| Bacteria | *Acetobacter* | *pasteurianus* IFO32830142C | 3.25 | 275 | TransportDB |
| Bacteria | *Acetobacter* | *pasteurianus* IFO328303 | 3.34 | 283 | TransportDB |
| Bacteria | *Acetobacter* | *pasteurianus* IFO328307 | 3.34 | 284 | TransportDB |
| Bacteria | *Acetobacter* | *pasteurianus* IFO328312 | 3.34 | 283 | TransportDB |
| Bacteria | *Acetobacter* | *pasteurianus* IFO328322 | 3.34 | 284 | TransportDB |
| Bacteria | *Acetobacter* | *pasteurianus* IFO328326 | 3.34 | 283 | TransportDB |
| Bacteria | *Acetobacter* | *pasteurianus* IFO328332 | 3.34 | 284 | TransportDB |
| Bacteria | *Anaplasma* | *phagocytophilum* HZ | 1.47 | 73 | TransportDB |
| Bacteria | *Anaplasma* | *phagocytophilum* str Dog2 | 1.47 | 78 | TransportDB |
| Bacteria | *Anaplasma* | *phagocytophilum* str HZ2 | 1.48 | 79 | TransportDB |
| Bacteria | *Anaplasma* | *phagocytophilum* str JM | 1.48 | 78 | TransportDB |
| Bacteria | *Arthrobacter* | *phenanthrenivorans* Sphe3 | 4.54 | 509 | TransportDB |
| Bacteria | *Arthrospira* | *platensis* NIES39 | 6.79 | 286 | TransportDB |
| Bacteria | *Actinobacillus* | *pleuropneumoniae* serovar3 str JL03 | 2.24 | 279 | TransportDB |
| Bacteria | *Actinobacillus* | *pleuropneumoniae* serovar5b str L20 | 2.27 | 283 | TransportDB |
| Bacteria | *Actinobacillus* | *pleuropneumoniae* serovar7 str AP76 | 2.35 | 290 | TransportDB |
| Bacteria | *Anaerococcus* | *prevotii* DSM20548 | 2.0 | 299 | TransportDB |
| Bacteria | *Agrobacterium* | *radiobacter* K84 | 7.27 | 1291 | TransportDB |
| Bacteria | *Aliivibrio* | *salmonicida* LFI1238 | 4.66 | 413 | TransportDB |
| Bacteria | *Aeromonas* | *salmonicida* subsp salmonicida A449 | 5.04 | 531 | TransportDB |
| Bacteria | *Alistipes* | *shahii* WAL8301 | 3.76 | 132 | TransportDB |
| Bacteria | *Anabaena* | *s90* | 5.31 | 280 | TransportDB |
| Bacteria | *Acinetobacter* | *sADP1* | 3.6 | 367 | TransportDB |
| Bacteria | *Azospirillum* | *sB510* | 7.6 | 858 | TransportDB |
| Bacteria | *Azoarcus* | *sBH72* | 4.38 | 406 | TransportDB |
| Bacteria | *Azoarcus* | *sEbN1* | 4.3 | 297 | TransportDB |
| Bacteria | *Arthrobacter* | *sFB24* | 5.07 | 577 | TransportDB |
| Bacteria | *Anaeromyxobacter* | *sFw1095* | 5.28 | 334 | TransportDB |
| Bacteria | *Agrobacterium* | *sH133* | 5.57 | 925 | TransportDB |
| Bacteria | *Acidovorax* | *sJS42* | 4.59 | 459 | TransportDB |
| Bacteria | *Anaeromyxobacter* | *sK* | 5.06 | 335 | TransportDB |
| Bacteria | *Azoarcus* | *sKH32C* | 5.82 | 526 | TransportDB |
| Bacteria | *Acidovorax* | *sKKS102* | 5.2 | 620 | TransportDB |
| Bacteria | *Arcobacter* | *sL* | 2.95 | 289 | TransportDB |
| Bacteria | *Actinoplanes* | *sN902109* | 9.23 | 679 | TransportDB |
| Bacteria | *Arthrobacter* | *sRue61a* | 5.08 | 621 | TransportDB |
| Bacteria | *Actinoplanes* | *sSE50110* | 9.24 | 645 | TransportDB |
| Bacteria | *Alteromonas* | *sSN2* | 4.97 | 327 | TransportDB |
| Bacteria | *Amycolicicoccus* | *subflavus* DQS39A1 | 4.86 | 458 | TransportDB |
| Bacteria | *Aequorivita* | *sublithincola* DSM14238 | 3.52 | 188 | TransportDB |
| Bacteria | *Actinobacillus* | *succinogenes* 130Z | 2.32 | 308 | TransportDB |
| Bacteria | *Actinobacillus* | *suis* H910380 | 2.48 | 315 | TransportDB |
| Bacteria | *Anaerolinea* | *thermophila* UNI1 | 3.53 | 350 | TransportDB |
| Bacteria | *Agrobacterium* | *tumefaciens* str C58 | 5.67 | 958 | TransportDB |
| Bacteria | *Aerococcus* | *urinae* ACS120VCol10a | 2.08 | 279 | TransportDB |
| Bacteria | *Anabaena* | *variabilis* ATCC29413 | 7.11 | 467 | TransportDB |
| Bacteria | *Aeromonas* | *veronii* B565 | 4.55 | 516 | TransportDB |
| Bacteria | *Azotobacter* | *vinelandii* CA | 5.37 | 516 | TransportDB |
| Bacteria | *Azotobacter* | *vinelandii* CA6 | 5.32 | 505 | TransportDB |
| Bacteria | *Azotobacter* | *vinelandii* DJ | 5.37 | 516 | TransportDB |
| Bacteria | *Allochromatium* | *vinosum* DSM180 | 3.67 | 266 | TransportDB |
| Bacteria | *Agrobacterium* | *vitis* S4 | 6.32 | 910 | TransportDB |
| Bacteria | *Acetobacterium* | *woodii* DSM1030 | 4.04 | 426 | TransportDB |
| Bacteria | *Amphibacillus* | *xylanus* NBRC15112 | 2.57 | 289 | TransportDB |
| Bacteria | *Achromobacter* | *xylosoxidans* A8 | 7.36 | 1160 | TransportDB |
| Bacteria | *Achromobacter* | *xylosoxidans* NBRC15126ATCC27061 | 6.68 | 914 | TransportDB |
| Bacteria | *Achromobacter* | *xylosoxidans* NH447841996 | 6.92 | 1014 | TransportDB |
| Bacteria | *Aster* | *yellows* witches broom phytoplasmaAYWB | 0.72 | 55 | TransportDB |
| Bacteria | *Brucella* | *abortus* A13334 | 3.29 | 489 | TransportDB |
| Bacteria | *Brucella* | *abortus* bv1str 9941 | 3.29 | 415 | TransportDB |
| Bacteria | *Brucella* | *abortus* S19 | 3.28 | 448 | TransportDB |
| Bacteria | *Bifidobacterium* | *adolescentis* ATCC15703 | 2.09 | 248 | TransportDB |
| Bacteria | *Borrelia* | *afzelii* HLJ01 | 0.91 | 92 | TransportDB |
| Bacteria | *Borrelia* | *afzelii* PKo | 1.23 | 97 | TransportDB |
| Bacteria | *Burkholderia* | *ambifaria* AMMD | 7.53 | 843 | TransportDB |
| Bacteria | *Burkholderia* | *ambifaria* MC406 | 7.64 | 821 | TransportDB |
| Bacteria | *Bacillus* | *amyloliquefaciens* CC178 | 3.92 | 474 | TransportDB |
| Bacteria | *Bacillus* | *amyloliquefaciens* DSM7 | 3.98 | 470 | TransportDB |
| Bacteria | *Bacillus* | *amyloliquefaciens* FZB42 | 3.92 | 485 | TransportDB |
| Bacteria | *Bacillus* | *amyloliquefaciens* IT45 | 3.94 | 480 | TransportDB |
| Bacteria | *Bacillus* | *amyloliquefaciens* LFB112 | 3.94 | 477 | TransportDB |
| Bacteria | *Bacillus* | *amyloliquefaciens* LL3 | 4.0 | 467 | TransportDB |
| Bacteria | *Bacillus* | *amyloliquefaciens* subsp plantarum AS433 | 3.96 | 480 | TransportDB |
| Bacteria | *Bacillus* | *amyloliquefaciens* subsp plantarum CAUB946 | 4.02 | 481 | TransportDB |
| Bacteria | *Bacillus* | *amyloliquefaciens* subsp plantarum NAUB3 | 4.20 | 502 | TransportDB |
| Bacteria | *Bacillus* | *amyloliquefaciens* subsp plantarum UCMB5033 | 4.07 | 475 | TransportDB |
| Bacteria | *Bacillus* | *amyloliquefaciens* subsp plantarum UCMB5036 | 3.91 | 482 | TransportDB |
| Bacteria | *Bacillus* | *amyloliquefaciens* subsp plantarum UCMB5113 | 3.89 | 476 | TransportDB |
| Bacteria | *Bacillus* | *amyloliquefaciens* subsp plantarum YAUB9601Y2 | 4.24 | 500 | TransportDB |
| Bacteria | *Bacillus* | *amyloliquefaciens* TA208 | 3.94 | 473 | TransportDB |
| Bacteria | *Bacillus* | *amyloliquefaciens* XH7 | 3.94 | 470 | TransportDB |
| Bacteria | *Bacillus* | *amyloliquefaciens* Y2 | 4.24 | 490 | TransportDB |
| Bacteria | *Bifidobacterium* | *animalis* subsp animalis ATCC25527 | 1.93 | 205 | TransportDB |
| Bacteria | *Bifidobacterium* | *animalis* subsp lactis AD011 | 1.93 | 208 | TransportDB |
| Bacteria | *Bifidobacterium* | *animalis* subsp lactis ATCC27673 | 1.96 | 211 | TransportDB |
| Bacteria | *Bifidobacterium* | *animalis* subsp lactis B420 | 1.94 | 214 | TransportDB |
| Bacteria | *Bifidobacterium* | *animalis* subsp lactis BB12 | 1.94 | 206 | TransportDB |
| Bacteria | *Bifidobacterium* | *animalis* subsp lactis Bi07 | 1.94 | 216 | TransportDB |
| Bacteria | *Bifidobacterium* | *animalis* subsp lactis Bl04 | 1.94 | 214 | TransportDB |
| Bacteria | *Bifidobacterium* | *animalis* subsp lactis Bl12 | 1.94 | 211 | TransportDB |
| Bacteria | *Bifidobacterium* | *animalis* subsp lactis BLC1 | 1.94 | 212 | TransportDB |
| Bacteria | *Bifidobacterium* | *animalis* subsp lactis CNCMI2494 | 1.94 | 206 | TransportDB |
| Bacteria | *Bifidobacterium* | *animalis* subsp lactis DSM10140 | 1.94 | 213 | TransportDB |
| Bacteria | *Bifidobacterium* | *animalis* subsp lactis V9 | 1.94 | 215 | TransportDB |
| Bacteria | *Bacillus* | *anthracis* str A0248 | 5.5 | 633 | TransportDB |
| Bacteria | *Bacillus* | *anthracis* str Ames | 5.23 | 628 | TransportDB |
| Bacteria | *Bacillus* | *anthracis* str Ames Ancestor | 5.5 | 637 | TransportDB |
| Bacteria | *Bacillus* | *anthracis* str CDC684 | 5.51 | 721 | TransportDB |
| Bacteria | *Bacillus* | *anthracis* str H9401 | 5.5 | 713 | TransportDB |
| Bacteria | *Bacillus* | *anthracis* str Sterne | 5.23 | 704 | TransportDB |
| Bacteria | *Buchnera* | *aphidicola* str 5A *Acyrthosiphon pisum* | 0.64 | 38 | TransportDB |
| Bacteria | *Buchnera* | *aphidicola* str Ak *Acyrthosiphon kondoi* | 0.65 | 40 | TransportDB |
| Bacteria | *Buchnera* | *aphidicola* str APS *Acyrthosiphon pisum* | 0.66 | 39 | TransportDB |
| Bacteria | *Buchnera* | *aphidicola* str Bp *Baizongiapistaciae* | 0.62 | 36 | TransportDB |
| Bacteria | *Buchnera* | *aphidicola* str JF98 *Acyrthosiphon pisum* | 0.64 | 27 | TransportDB |
| Bacteria | *Buchnera* | *aphidicola* str JF99 *Acyrthosiphon pisum* | 0.64 | 39 | TransportDB |
| Bacteria | *Buchnera* | *aphidicola* str LL01 *Acyrthosiphon pisum* | 0.64 | 38 | TransportDB |
| Bacteria | *Buchnera* | *aphidicola* str Sg *Schizaphis graminum* | 0.64 | 44 | TransportDB |
| Bacteria | *Buchnera* | *aphidicola* str TLW03 *Acyrthosiphon pisum* | 0.64 | 36 | TransportDB |
| Bacteria | *Buchnera* | *aphidicola* str Tuc7 *Acyrthosiphon pisum* | 0.64 | 38 | TransportDB |
| Bacteria | *Buchnera* | *aphidicola* str Ua *Uroleucon ambrosiae* | 0.63 | 39 | TransportDB |
| Bacteria | *Bifidobacterium* | *asteroides* PRL2011 | 2.17 | 283 | TransportDB |
| Bacteria | *Bacillus* | *atrophaeus* 1942 | 4.17 | 504 | TransportDB |
| Bacteria | *Bartonella* | *australis* Aust NH1 | 1.6 | 143 | TransportDB |
| Bacteria | *Bordetella* | *avium* 197N | 3.73 | 482 | TransportDB |
| Bacteria | *Bartonella* | *bacilliformis* KC583 | 1.45 | 125 | TransportDB |
| Bacteria | *Bdellovibrio* | *bacteriovorus* HD100 | 3.78 | 284 | TransportDB |
| Bacteria | *Bdellovibrio* | *bacteriovorus* str Tiberius | 3.99 | 284 | TransportDB |
| Bacteria | *Belliella* | *baltica* DSM15883 | 4.20 | 211 | TransportDB |
| Bacteria | *Bifidobacterium* | *bifidum* BGN4 | 2.22 | 197 | TransportDB |
| Bacteria | *Bifidobacterium* | *bifidum* PRL2010 | 2.21 | 194 | TransportDB |
| Bacteria | *Bifidobacterium* | *bifidum* S17 | 2.19 | 193 | TransportDB |
| Bacteria | *Borrelia* | *bissettii* DN127 | 1.4 | 95 | TransportDB |
| Bacteria | *Bifidobacterium* | *breve* ACS071VSch8b | 2.33 | 304 | TransportDB |
| Bacteria | *Bifidobacterium* | *breve* UCC2003 | 2.42 | 303 | TransportDB |
| Bacteria | *Brevibacillus* | *brevis* NBRC100599 | 6.3 | 765 | TransportDB |
| Bacteria | *Bordetella* | *bronchiseptica* 253 | 5.26 | 877 | TransportDB |
| Bacteria | *Bordetella* | *bronchiseptica* MO149 | 5.09 | 872 | TransportDB |
| Bacteria | *Bordetella* | *bronchiseptica* RB50 | 5.34 | 889 | TransportDB |
| Bacteria | *Borrelia* | *burgdorferi* B31 | 1.52 | 86 | TransportDB |
| Bacteria | *Borrelia* | *burgdorferi* CA382 | 0.91 | 86 | TransportDB |
| Bacteria | *Borrelia* | *burgdorferi* JD1 | 1.53 | 95 | TransportDB |
| Bacteria | *Borrelia* | *burgdorferi* N40 | 1.34 | 96 | TransportDB |
| Bacteria | *Borrelia* | *burgdorferi* ZS7 | 1.35 | 90 | TransportDB |
| Bacteria | *Brucella* | *canis* ATCC23365 | 3.31 | 440 | TransportDB |
| Bacteria | *Brucella* | *canis* HSKA52141 | 3.28 | 467 | TransportDB |
| Bacteria | *Beutenbergia* | *cavernae* DSM12333 | 4.67 | 635 | TransportDB |
| Bacteria | *Bacillus* | *cellulosilyticus* DSM2522 | 4.68 | 458 | TransportDB |
| Bacteria | *Burkholderia* | *cenocepacia* AU1054 | 7.28 | 905 | TransportDB |
| Bacteria | *Burkholderia* | *cenocepacia* HI2424 | 7.7 | 945 | TransportDB |
| Bacteria | *Burkholderia* | *cenocepacia* J2315 | 8.06 | 926 | TransportDB |
| Bacteria | *Burkholderia* | *cepacia* 383 (R18194) | 8.68 | 930 | TransportDB |
| Bacteria | *Bacillus* | *cereus* 03BB102 | 5.45 | 673 | TransportDB |
| Bacteria | *Bacillus* | *cereus* AH187 | 5.60 | 696 | TransportDB |
| Bacteria | *Bacillus* | *cereus* AH820 | 5.59 | 699 | TransportDB |
| Bacteria | *Bacillus* | *cereus* ATCC10987 | 5.43 | 695 | TransportDB |
| Bacteria | *Bacillus* | *cereus* ATCC14579 | 5.43 | 642 | TransportDB |
| Bacteria | *Bacillus* | *cereus* B4264 | 5.42 | 652 | TransportDB |
| Bacteria | *Bacillus* | *cereus* biovar anthracis str CI | 5.49 | 703 | TransportDB |
| Bacteria | *Bacillus* | *cereus* E33L | 5.84 | 729 | TransportDB |
| Bacteria | *Bacillus* | *cereus* F83776 | 5.29 | 674 | TransportDB |
| Bacteria | *Bacillus* | *cereus* FRI35 | 5.38 | 702 | TransportDB |
| Bacteria | *Bacillus* | *cereus* G9842 | 5.74 | 672 | TransportDB |
| Bacteria | *Bacillus* | *cereus* NC7401 | 5.55 | 693 | TransportDB |
| Bacteria | *Bacillus* | *cereus* Q1 | 5.51 | 710 | TransportDB |
| Bacteria | *Brucella* | *ceti* TE1075912 | 3.28 | 433 | TransportDB |
| Bacteria | *Brucella* | *ceti* TE2875312 | 3.28 | 375 | TransportDB |
| Bacteria | *Baumannia* | *cicadellinicola* str Hc *Homalodisca coagulata* | 0.69 | 49 | TransportDB |
| Bacteria | *Bartonella* | *clarridgeiae* 73 | 1.52 | 136 | TransportDB |
| Bacteria | *Bacillus* | *clausii* KSMK16 | 4.3 | 656 | TransportDB |
| Bacteria | *Bacillus* | *coagulans* 26 | 3.07 | 350 | TransportDB |
| Bacteria | *Bacillus* | *coagulans* 36D1 | 3.55 | 420 | TransportDB |
| Bacteria | *Borrelia* | *crocidurae* str Achema | 1.53 | 92 | TransportDB |
| Bacteria | *Bacillus* | *cytotoxicus* NVH39198 | 4.09 | 486 | TransportDB |
| Bacteria | *Bifidobacterium* | *dentium* Bd1 | 2.64 | 347 | TransportDB |
| Bacteria | *Borrelia* | *duttonii* Ly | 1.57 | 93 | TransportDB |
| Bacteria | *Bdellovibrio* | *exovorus* JSS | 2.66 | 203 | TransportDB |
| Bacteria | *Brachybacterium* | *faecium* DSM4810 | 3.61 | 464 | TransportDB |
| Bacteria | *Butyrivibrio* | *fibrisolvens* 164 | 3.16 | 283 | TransportDB |
| Bacteria | *Bacteroides* | *fragilis* 638R | 5.37 | 298 | TransportDB |
| Bacteria | *Bacteroides* | *fragilis* NCTC9343 | 5.24 | 290 | TransportDB |
| Bacteria | *Bacteroides* | *fragilis* YCH46 | 5.31 | 297 | TransportDB |
| Bacteria | *Borrelia* | *garinii* BgVir | 0.99 | 95 | TransportDB |
| Bacteria | *Borrelia* | *garinii* NMJW1 | 0.90 | 88 | TransportDB |
| Bacteria | *Borrelia* | *garinii* PBi | 0.99 | 95 | TransportDB |
| Bacteria | *Burkholderia* | *gladioli* BSR3 | 9.05 | 896 | TransportDB |
| Bacteria | *Burkholderia* | *glumae* BGR1 | 7.28 | 605 | TransportDB |
| Bacteria | *Bartonella* | *grahamii* as4aup | 2.37 | 187 | TransportDB |
| Bacteria | *Bacillus* | *halodurans* C125 | 4.2 | 539 | TransportDB |
| Bacteria | *Bacteroides* | *helcogenes* P36108 | 4.0 | 208 | TransportDB |
| Bacteria | *Bartonella* | *henselae* str Houston1 | 1.93 | 155 | TransportDB |
| Bacteria | *Borrelia* | *hermsii* DAH | 0.92 | 85 | TransportDB |
| Bacteria | *Brachyspira* | *hyodysenteriae* WA1 | 3.04 | 285 | TransportDB |
| Bacteria | *Beijerinckia* | *indica* subsp indica ATCC9039 | 4.42 | 369 | TransportDB |
| Bacteria | *Bacillus* | *infantis* NRRLB14911 | 4.88 | 582 | TransportDB |
| Bacteria | *Brachyspira* | *intermedia* PWSA | 3.31 | 300 | TransportDB |
| Bacteria | *Bradyrhizobium* | *japonicum* USDA110 | 9.11 | 1107 | TransportDB |
| Bacteria | *Bradyrhizobium* | *japonicum* USDA6 | 9.21 | 1138 | TransportDB |
| Bacteria | *Bacillus* | *licheniformis* 9945A | 4.38 | 599 | TransportDB |
| Bacteria | *Bacillus* | *licheniformis* DSM13ATCC14580 | 4.22 | 567 | TransportDB |
| Bacteria | *Bifidobacterium* | *longum* DJO10A | 2.39 | 276 | TransportDB |
| Bacteria | *Bifidobacterium* | *longum* NCC2705 | 2.26 | 253 | TransportDB |
| Bacteria | *Bifidobacterium* | *longum* subsp infantis 157F | 2.41 | 273 | TransportDB |
| Bacteria | *Bifidobacterium* | *longum* subsp infantis ATCC15697JCM1222 | 2.83 | 351 | TransportDB |
| Bacteria | *Bifidobacterium* | *longum* subsp longum BBMN68 | 2.27 | 275 | TransportDB |
| Bacteria | *Bifidobacterium* | *longum* subsp longum F8 | 2.38 | 233 | TransportDB |
| Bacteria | *Bifidobacterium* | *longum* subsp longum JCM1217 | 2.39 | 255 | TransportDB |
| Bacteria | *Bifidobacterium* | *longum* subsp longum JDM301 | 2.48 | 314 | TransportDB |
| Bacteria | *Bifidobacterium* | *longum* subsp longum KACC91563 | 2.4 | 272 | TransportDB |
| Bacteria | *Burkholderia* | *mallei* ATCC23344 | 5.84 | 558 | TransportDB |
| Bacteria | *Burkholderia* | *mallei* NCTC10229 | 5.74 | 597 | TransportDB |
| Bacteria | *Burkholderia* | *mallei* NCTC10247 | 5.85 | 577 | TransportDB |
| Bacteria | *Burkholderia* | *mallei* SAVP1 | 5.23 | 541 | TransportDB |
| Bacteria | *Bacteriovorax* | *marinus* SJ | 3.44 | 213 | TransportDB |
| Bacteria | *Bacillus* | *megaterium* DSM319 | 5.1 | 667 | TransportDB |
| Bacteria | *Bacillus* | *megaterium* QMB1551 | 5.52 | 700 | TransportDB |
| Bacteria | *Bacillus* | *megaterium* WSH002 | 5.08 | 652 | TransportDB |
| Bacteria | *Brucella* | *melitensis* ATCC23457 | 3.31 | 425 | TransportDB |
| Bacteria | *Brucella* | *melitensis* biovar Abortus 2308 | 3.28 | 417 | TransportDB |
| Bacteria | *Brucella* | *melitensis* bv ovis ATCC25840 | 3.28 | 392 | TransportDB |
| Bacteria | *Brucella* | *melitensis* bv suis 1330 | 3.31 | 431 | TransportDB |
| Bacteria | *Brucella* | *melitensis* bv1str16M | 3.29 | 476 | TransportDB |
| Bacteria | *Brucella* | *melitensis* M28 | 3.31 | 479 | TransportDB |
| Bacteria | *Brucella* | *melitensis* M590 | 3.31 | 480 | TransportDB |
| Bacteria | *Brucella* | *melitensis* NI | 3.29 | 479 | TransportDB |
| Bacteria | *Brucella* | *microti* CCM4915 | 3.34 | 462 | TransportDB |
| Bacteria | *Burkholderia* | *multivorans* ATCC17616 | 7.01 | 810 | TransportDB |
| Bacteria | *Brachyspira* | *murdochii* DSM12563 | 3.24 | 291 | TransportDB |
| Bacteria | *Bradyrhizobium* | *oligotrophicum* S58 | 8.26 | 953 | TransportDB |
| Bacteria | *Brucella* | *ovis* ATCC25840 | 3.28 | 393 | TransportDB |
| Bacteria | *Bordetella* | *parapertussis* 12822 | 4.77 | 751 | TransportDB |
| Bacteria | *Bordetella* | *parapertussis* Bpp5 | 4.9 | 722 | TransportDB |
| Bacteria | *Bordetella* | *pertussis* 18323 | 4.04 | 501 | TransportDB |
| Bacteria | *Bordetella* | *pertussis* CS | 4.12 | 547 | TransportDB |
| Bacteria | *Bordetella* | *pertussis* TohamaI | 4.09 | 539 | TransportDB |
| Bacteria | *Bordetella* | *petrii* DSM12804 | 5.29 | 772 | TransportDB |
| Bacteria | *Burkholderia* | *phenoliruptrix* BR3459a | 7.65 | 873 | TransportDB |
| Bacteria | *Burkholderia* | *phymatum* STM815 | 8.68 | 938 | TransportDB |
| Bacteria | *Burkholderia* | *phytofirmans* PsJN | 8.21 | 999 | TransportDB |
| Bacteria | *Brachyspira* | *pilosicoli* 951000 | 2.59 | 262 | TransportDB |
| Bacteria | *Brachyspira* | *pilosicoli* B2904 | 2.77 | 291 | TransportDB |
| Bacteria | *Brachyspira* | *pilosicoli* P43678 | 2.56 | 237 | TransportDB |
| Bacteria | *Brachyspira* | *pilosicoli* WesB | 2.89 | 308 | TransportDB |
| Bacteria | *Brucella* | *pinnipedialis* B294 | 3.4 | 455 | TransportDB |
| Bacteria | *Butyrivibrio* | *proteoclasticus* B316 | 4.4 | 357 | TransportDB |
| Bacteria | *Bacillus* | *pseudofirmus* OF4 | 4.25 | 473 | TransportDB |
| Bacteria | *Burkholderia* | *pseudomallei* 1026b | 7.23 | 700 | TransportDB |
| Bacteria | *Burkholderia* | *pseudomallei* 1106a | 7.09 | 695 | TransportDB |
| Bacteria | *Burkholderia* | *pseudomallei* 1710b | 7.31 | 690 | TransportDB |
| Bacteria | *Burkholderia* | *pseudomallei* 668 | 7.04 | 702 | TransportDB |
| Bacteria | *Burkholderia* | *pseudomallei* BPC006 | 7.16 | 689 | TransportDB |
| Bacteria | *Burkholderia* | *pseudomallei* K96243 | 7.25 | 703 | TransportDB |
| Bacteria | *Bacillus* | *pumilus* SAFR032 | 3.7 | 477 | TransportDB |
| Bacteria | *Bartonella* | *quintana* RM11 | 1.59 | 148 | TransportDB |
| Bacteria | *Bartonella* | *quintana* str Toulouse | 1.58 | 140 | TransportDB |
| Bacteria | *Borrelia* | *recurrentis* A1 | 1.24 | 89 | TransportDB |
| Bacteria | *Burkholderia* | *rhizoxinica* HKI454 | 3.75 | 270 | TransportDB |
| Bacteria | *Bacteroides* | *salanitronis* DSM18170 | 4.31 | 194 | TransportDB |
| Bacteria | *Blastococcus* | *saxobsidens* DD2 | 4.88 | 440 | TransportDB |
| Bacteria | *Bacillus* | *selenitireducens* MLS10 | 3.59 | 381 | TransportDB |
| Bacteria | *Bacillus* | *s1NLA3E* | 4.82 | 515 | TransportDB |
| Bacteria | *Burkholderia* | *s383* | 8.68 | 972 | TransportDB |
| Bacteria | *Blattabacterium* | *sblaberus giganteus* | 0.63 | 40 | TransportDB |
| Bacteria | *Blattabacterium* | *sBlatta orientalis* str Tarazona | 0.64 | 38 | TransportDB |
| Bacteria | *Blattabacterium* | *sBlattella germanica* str Bge | 0.64 | 40 | TransportDB |
| Bacteria | *Bradyrhizobium* | *sBTAi1* | 8.49 | 1039 | TransportDB |
| Bacteria | *Burkholderia* | *sCCGE1001* | 6.83 | 791 | TransportDB |
| Bacteria | *Burkholderia* | *sCCGE1002* | 7.88 | 899 | TransportDB |
| Bacteria | *Burkholderia* | *sCCGE1003* | 7.04 | 811 | TransportDB |
| Bacteria | *Bacteroides* | *sCF* | 2.66 | 165 | TransportDB |
| Bacteria | *Blattabacterium* | *scryptocercus punctulatus* str Cpu | 0.61 | 36 | TransportDB |
| Bacteria | *Bacillus* | *sJS* | 4.12 | 543 | TransportDB |
| Bacteria | *Burkholderia* | *sKJ006* | 6.63 | 707 | TransportDB |
| Bacteria | *Blattabacterium* | *smastotermesdarwiniensis* str MADAR | 0.59 | 37 | TransportDB |
| Bacteria | *Blattabacterium* | *snauphoetacinerea* | 0.63 | 41 | TransportDB |
| Bacteria | *Bradyrhizobium* | *sORS278* | 7.46 | 857 | TransportDB |
| Bacteria | *Blattabacterium* | *spanesthia angustipennis spadica* str BPAA | 0.63 | 39 | TransportDB |
| Bacteria | *Blattabacterium* | *speriplaneta americana* str BPLAN | 0.64 | 38 | TransportDB |
| Bacteria | *Burkholderia* | *sRPE64* | 6.96 | 852 | TransportDB |
| Bacteria | *Bradyrhizobium* | *sS23321* | 7.23 | 957 | TransportDB |
| Bacteria | *Burkholderia* | *sYI23* | 8.9 | 1083 | TransportDB |
| Bacteria | *Bacillus* | *subtilis* BSn5 | 4.09 | 553 | TransportDB |
| Bacteria | *Bacillus* | *subtilis* PY79 | 4.03 | 549 | TransportDB |
| Bacteria | *Bacillus* | *subtilis* QB928 | 4.15 | 549 | TransportDB |
| Bacteria | *Bacillus* | *subtilis* subsp natto BEST195 | 4.1 | 550 | TransportDB |
| Bacteria | *Bacillus* | *subtilis* subsp spizizenii str W23 | 4.03 | 527 | TransportDB |
| Bacteria | *Bacillus* | *subtilis* subsp spizizenii TUB10 | 4.21 | 544 | TransportDB |
| Bacteria | *Bacillus* | *subtilis* subsp subtilis 6051HGW | 4.22 | 556 | TransportDB |
| Bacteria | *Bacillus* | *subtilis* subsp subtilis str 168 | 4.22 | 559 | TransportDB |
| Bacteria | *Bacillus* | *subtilis* subsp subtilis str BAB1 | 4.02 | 544 | TransportDB |
| Bacteria | *Bacillus* | *subtilis* subsp subtilis str BSP1 | 4.04 | 546 | TransportDB |
| Bacteria | *Bacillus* | *subtilis* subsp subtilis str RONN1 | 4.01 | 529 | TransportDB |
| Bacteria | *Bacillus* | *subtilis* XF1 | 4.06 | 525 | TransportDB |
| Bacteria | *Brevundimonas* | *subvibrioides* ATCC15264 | 3.45 | 222 | TransportDB |
| Bacteria | *Brucella* | *suis 1330* | 3.32 | 438 | TransportDB |
| Bacteria | *Brucella* | *suis* ATCC23445 | 3.32 | 434 | TransportDB |
| Bacteria | *Brucella* | *suis* VBI22 | 3.32 | 438 | TransportDB |
| Bacteria | *Burkholderia* | *thailandensis* E264 | 6.72 | 669 | TransportDB |
| Bacteria | *Burkholderia* | *thailandensis* MSMB121 | 6.73 | 768 | TransportDB |
| Bacteria | *Bifidobacterium* | *thermophilum* RBL67 | 2.29 | 230 | TransportDB |
| Bacteria | *Bacteroides* | *thetaiotaomicron* VPI5482 | 6.29 | 306 | TransportDB |
| Bacteria | *Bacillus* | *thuringiensis* BMB171 | 5.64 | 660 | TransportDB |
| Bacteria | *Bacillus* | *thuringiensis* Bt407 | 6.13 | 684 | TransportDB |
| Bacteria | *Bacillus* | *thuringiensis* HD771 | 6.44 | 681 | TransportDB |
| Bacteria | *Bacillus* | *thuringiensis* HD789 | 6.33 | 667 | TransportDB |
| Bacteria | *Bacillus* | *thuringiensis* MC28 | 6.69 | 725 | TransportDB |
| Bacteria | *Bacillus* | *thuringiensis* serovar chinensis CT43 | 6.15 | 687 | TransportDB |
| Bacteria | *Bacillus* | *thuringiensis* serovar finitimus YBT020 | 5.68 | 702 | TransportDB |
| Bacteria | *Bacillus* | *thuringiensis* serovar konkukian str 9727 | 5.31 | 693 | TransportDB |
| Bacteria | *Bacillus* | *thuringiensis* serovar kurstaki str HD73 | 5.91 | 673 | TransportDB |
| Bacteria | *Bacillus* | *thuringiensis* serovar thuringiensis str IS5056 | 6.77 | 738 | TransportDB |
| Bacteria | *Bacillus* | *thuringiensis* str AlHakam | 5.31 | 667 | TransportDB |
| Bacteria | *Bacillus* | *thuringiensis* YBT1518 | 6.67 | 707 | TransportDB |
| Bacteria | *Bacillus* | *toyonensis* BCT7112 | 5.03 | 642 | TransportDB |
| Bacteria | *Bibersteinia* | *trehalosi* USDAARSUSMARC192 | 2.41 | 289 | TransportDB |
| Bacteria | *Bartonella* | *tribocorum* CIP105476 | 2.64 | 189 | TransportDB |
| Bacteria | *Borrelia* | *turicatae* 91E135 | 0.92 | 87 | TransportDB |
| Bacteria | *Burkholderia* | *vietnamiensis* G4 | 8.39 | 809 | TransportDB |
| Bacteria | *Bartonella* | *vinsonii* subsp berkhoffiistr Winnie | 1.80 | 155 | TransportDB |
| Bacteria | *Bacteroides* | *vulgatus* ATCC8482 | 5.16 | 268 | TransportDB |
| Bacteria | *Bacillus* | *weihenstephanensis* KBAB4 | 5.87 | 678 | TransportDB |
| Bacteria | *Burkholderia* | *xenovorans* LB400 | 9.73 | 1157 | TransportDB |
| Bacteria | *Bacteroides* | *xylanisolvens* XB1A | 5.98 | 261 | TransportDB |
| Bacteria | *Chlamydophila* | *abortus* S263 | 1.14 | 96 | TransportDB |
| Bacteria | *Candidatus* | *accumulibacter* phosphatisclade IIA str UW1 | 5.31 | 438 | TransportDB |
| Bacteria | *Clostridium* | *acetobutylicum* ATCC824 | 4.13 | 424 | TransportDB |
| Bacteria | *Clostridium* | *acetobutylicum* DSM1731 | 4.15 | 426 | TransportDB |
| Bacteria | *Clostridium* | *acetobutylicum* EA2018 | 4.13 | 428 | TransportDB |
| Bacteria | *Catenulispora* | *acidiphila* DSM44928 | 10.47 | 894 | TransportDB |
| Bacteria | *Clostridium* | *acidurici* 9a | 3.11 | 380 | TransportDB |
| Bacteria | *Caldilinea* | *aerophila* DSM14535NBRC104270 | 5.14 | 588 | TransportDB |
| Bacteria | *Chloroflexus* | *aggregans* DSM9485 | 4.68 | 359 | TransportDB |
| Bacteria | *Coraliomargarita* | *akajimensis* DSM45221 | 3.75 | 199 | TransportDB |
| Bacteria | *Cellulophaga* | *algicola* DSM14237 | 4.89 | 253 | TransportDB |
| Bacteria | *Candidatus* | *amoebophilus asiaticus* 5a2 | 1.88 | 100 | TransportDB |
| Bacteria | *Cyanobacterium* | *aponinum* PCC10605 | 4.18 | 279 | TransportDB |
| Bacteria | *Corynebacterium* | *argentoratense* DSM44202 | 2.03 | 212 | TransportDB |
| Bacteria | *Candidatus* | *Arthromitus* sp SFBmouse Japan | 1.62 | 151 | TransportDB |
| Bacteria | *Candidatus* | *arthromitus* sp SFB mouse Yit | 1.59 | 155 | TransportDB |
| Bacteria | *Candidatus* | *arthromitus* sp SFB rat Yit | 1.52 | 156 | TransportDB |
| Bacteria | *Croceibacter* | *atlanticus* HTCC2559 | 2.95 | 176 | TransportDB |
| Bacteria | *Chloroflexus* | *aurantiacus* J10fl | 5.26 | 398 | TransportDB |
| Bacteria | *Corynebacterium* | *aurimucosum* ATCC700975 | 2.82 | 302 | TransportDB |
| Bacteria | *Clostridium* | *autoethanogenum* DSM10061 | 4.35 | 468 | TransportDB |
| Bacteria | *Candidatus* | *azobacteroides pseudo trichonymphae genomovar* CFP2 | 1.22 | 50 | TransportDB |
| Bacteria | *Clostridium* | *beijerinckii* NCIMB8052 | 6.0 | 680 | TransportDB |
| Bacteria | *Caldicellulosiruptor* | *bescii* DSM6725 | 2.93 | 254 | TransportDB |
| Bacteria | *Candidatus* | *blochmannia chromaiodes* str 640 | 0.79 | 47 | TransportDB |
| Bacteria | *Candidatus* | *blochmannia floridanus* | 0.71 | 46 | TransportDB |
| Bacteria | *Candidatus* | *blochmannia pennsylvanicus* str BPEN | 0.79 | 47 | TransportDB |
| Bacteria | *Candidatus* | *blochmannia vafer* str BVAF | 0.72 | 47 | TransportDB |
| Bacteria | *Clostridium* | *botulinum* A2 str Kyoto | 4.16 | 464 | TransportDB |
| Bacteria | *Clostridium* | *botulinum* A3 str Loch Maree | 4.26 | 485 | TransportDB |
| Bacteria | *Clostridium* | *botulinum* A str ATCC19397 | 3.86 | 450 | TransportDB |
| Bacteria | *Clostridium* | *botulinum* A str ATCC3502 | 3.9 | 459 | TransportDB |
| Bacteria | *Clostridium* | *botulinum* A str Hall | 3.76 | 446 | TransportDB |
| Bacteria | *Clostridium* | *botulinum* B1 str Okra | 4.11 | 464 | TransportDB |
| Bacteria | *Clostridium* | *botulinum* Ba4 str 657 | 4.26 | 455 | TransportDB |
| Bacteria | *Coxiella* | *burnetii* CbuG_Q212 | 2.01 | 152 | TransportDB |
| Bacteria | *Coxiella* | *burnetii* CbuK_Q154 | 2.1 | 149 | TransportDB |
| Bacteria | *Coxiella* | *burnetii* Dugway 5J108111 | 2.21 | 167 | TransportDB |
| Bacteria | *Coxiella* | *burnetii* RSA331 | 2.05 | 145 | TransportDB |
| Bacteria | *Coxiella* | *burnetii* RSA493 | 2.03 | 154 | TransportDB |
| Bacteria | *Chthonomonas* | *calidirosea* T49 | 3.44 | 194 | TransportDB |
| Bacteria | *Corynebacterium* | *callunae* DSM20147 | 2.93 | 325 | TransportDB |
| Bacteria | *Capnocytophaga* | *canimorsus* Cc5 | 2.57 | 139 | TransportDB |
| Bacteria | *Coprococcus* | *catus* GD7 | 3.52 | 350 | TransportDB |
| Bacteria | *Chlamydophila* | *caviae* GPIC | 1.18 | 91 | TransportDB |
| Bacteria | *Clostridium* | *cellulolyticum* H10 | 4.07 | 307 | TransportDB |
| Bacteria | *Clostridium* | *cellulovorans* 743B | 5.26 | 433 | TransportDB |
| Bacteria | *Clostridium* | *csaccharolyticum* K10 | 3.77 | 344 | TransportDB |
| Bacteria | *Candidatus* | *chloracidobacterium thermophilum* B | 3.7 | 187 | TransportDB |
| Bacteria | *Chlorobium* | *chlorochromatii* CaD3 | 2.57 | 133 | TransportDB |
| Bacteria | *Clostridium* | *clariflavum* DSM19732 | 4.9 | 300 | TransportDB |
| Bacteria | *Candidatus* | *cloacamonas acidaminovorans* | 2.25 | 122 | TransportDB |
| Bacteria | *Campylobacter* | *coli* 15537360 | 1.69 | 206 | TransportDB |
| Bacteria | *Campylobacter* | *coli* 76339 | 1.58 | 195 | TransportDB |
| Bacteria | *Campylobacter* | *coli* CVMN29710 | 1.73 | 200 | TransportDB |
| Bacteria | *Campylobacter* | *concisus* 13826 | 2.1 | 176 | TransportDB |
| Bacteria | *Corallococcus* | *coralloides* DSM2259 | 10.08 | 439 | TransportDB |
| Bacteria | *Caulobacter* | *crescentus* CB15 | 4.02 | 262 | TransportDB |
| Bacteria | *Caulobacter* | *crescentus* NA1000 | 4.04 | 264 | TransportDB |
| Bacteria | *Cryptobacterium* | *curtum* DSM15641 | 1.62 | 144 | TransportDB |
| Bacteria | *Campylobacter* | *curvus* 52592 | 1.97 | 207 | TransportDB |
| Bacteria | *Candidatus* | *desulforudis audaxviator* MP104C | 2.35 | 176 | TransportDB |
| Bacteria | *Clostridium* | *difficile* 630 | 4.3 | 517 | TransportDB |
| Bacteria | *Clostridium* | *difficile* BI1 | 4.46 | 532 | TransportDB |
| Bacteria | *Clostridium* | *difficile* CD196 | 4.11 | 518 | TransportDB |
| Bacteria | *Clostridium* | *difficile* R20291 | 4.19 | 526 | TransportDB |
| Bacteria | *Corynebacterium* | *diphtheriae* 241 | 2.43 | 272 | TransportDB |
| Bacteria | *Corynebacterium* | *diphtheriae* 31A | 2.54 | 282 | TransportDB |
| Bacteria | *Corynebacterium* | *diphtheriae* BH8 | 2.49 | 279 | TransportDB |
| Bacteria | *Corynebacterium* | *diphtheriae* C7beta | 2.5 | 286 | TransportDB |
| Bacteria | *Corynebacterium* | *diphtheriae* CDCE8392 | 2.43 | 278 | TransportDB |
| Bacteria | *Corynebacterium* | *diphtheriae* HC01 | 2.43 | 270 | TransportDB |
| Bacteria | *Corynebacterium* | *diphtheriae* HC02 | 2.47 | 268 | TransportDB |
| Bacteria | *Corynebacterium* | *diphtheriae* HC03 | 2.48 | 267 | TransportDB |
| Bacteria | *Corynebacterium* | *diphtheriae* HC04 | 2.48 | 264 | TransportDB |
| Bacteria | *Corynebacterium* | *diphtheriae* INCA402 | 2.45 | 281 | TransportDB |
| Bacteria | *Corynebacterium* | *diphtheriae* NCTC13129 | 2.49 | 263 | TransportDB |
| Bacteria | *Corynebacterium* | *diphtheriae* PW8 | 2.53 | 272 | TransportDB |
| Bacteria | *Corynebacterium* | *diphtheriae* VA01 | 2.4 | 267 | TransportDB |
| Bacteria | *Candidate* | *division* SR1bacterium RAAC1_SR1_1 | 1.18 | 62 | TransportDB |
| Bacteria | *Candidate* | *division* WWE3 bacterium RAAC2_WWE3_1 | 0.88 | 49 | TransportDB |
| Bacteria | *Corynebacterium* | *efficiens* YS314 | 3.22 | 344 | TransportDB |
| Bacteria | *Candidatus* | *endolissoclinum patella* L2 | 1.48 | 72 | TransportDB |
| Bacteria | *Cardinium* | *endosymbiont* cEper1 of *Encarsia pergandiella* | 0.94 | 71 | TransportDB |
| Bacteria | *Crinalium* | *epipsammum* PCC9333 | 5.62 | 280 | TransportDB |
| Bacteria | *Caldisericum* | *exile* AZM16c01 | 1.56 | 192 | TransportDB |
| Bacteria | *Chlamydophila* | *felis* FeC56 | 1.17 | 94 | TransportDB |
| Bacteria | *Campylobacter* | *fetus* subsp fetus8240 | 1.77 | 203 | TransportDB |
| Bacteria | *Campylobacter* | *fetus* subsp testudinum03427 | 1.78 | 210 | TransportDB |
| Bacteria | *Cellulomonas* | *fimi* ATCC484 | 4.27 | 452 | TransportDB |
| Bacteria | *Cellulomonas* | *flavigena* DSM20109 | 4.12 | 351 | TransportDB |
| Bacteria | *Collimonas* | *fungivorans* Ter331 | 5.19 | 561 | TransportDB |
| Bacteria | *Clostridiales* | *genomo* sBVAB3 str UPII95 | 1.81 | 239 | TransportDB |
| Bacteria | *Coriobacterium* | *glomerans* PW2 | 2.12 | 317 | TransportDB |
| Bacteria | *Corynebacterium* | *glutamicum* ATCC13032 | 3.28 | 388 | TransportDB |
| Bacteria | *Corynebacterium* | *glutamicum* K051 | 3.31 | 386 | TransportDB |
| Bacteria | *Corynebacterium* | *glutamicum* MB001 | 3.08 | 380 | TransportDB |
| Bacteria | *Corynebacterium* | *glutamicum* R | 3.36 | 430 | TransportDB |
| Bacteria | *Corynebacterium* | *glutamicum* SCgG1 | 3.35 | 432 | TransportDB |
| Bacteria | *Corynebacterium* | *glutamicum* SCgG2 | 3.35 | 431 | TransportDB |
| Bacteria | *Cyanobium* | *gracile* PCC6307 | 3.34 | 260 | TransportDB |
| Bacteria | *Corynebacterium* | *halotolerans* YIM70093DSM44683 | 3.22 | 343 | TransportDB |
| Bacteria | *Candidatus* | *hamiltonella defensa* 5AT *Acyrthosiphon pisum* | 2.17 | 153 | TransportDB |
| Bacteria | *Campylobacter* | *hominis* ATCCBAA381 | 1.71 | 150 | TransportDB |
| Bacteria | *Cytophaga* | *hutchinsonii* ATCC33406 | 4.43 | 219 | TransportDB |
| Bacteria | *Carboxydothermus* | *hydrogenoformans* Z2901 | 2.4 | 201 | TransportDB |
| Bacteria | *Caldicellulosiruptor* | *hydrothermalis* 108 | 2.77 | 312 | TransportDB |
| Bacteria | *Cellvibrio* | *japonicus* Ueda107 | 4.58 | 310 | TransportDB |
| Bacteria | *Corynebacterium* | *jeikeium* K411 | 2.48 | 239 | TransportDB |
| Bacteria | *Campylobacter* | *jejuni* 4031 | 1.67 | 208 | TransportDB |
| Bacteria | *Campylobacter* | *jejuni* RM1221 | 1.78 | 190 | TransportDB |
| Bacteria | *Campylobacter* | *jejuni* subsp doylei26997 | 1.85 | 180 | TransportDB |
| Bacteria | *Campylobacter* | *jejuni* subsp jejuni002425 | 1.72 | 197 | TransportDB |
| Bacteria | *Campylobacter* | *jejuni* subsp jejuni002426 | 1.62 | 213 | TransportDB |
| Bacteria | *Campylobacter* | *jejuni* subsp jejuni002538 | 1.66 | 211 | TransportDB |
| Bacteria | *Campylobacter* | *jejuni* subsp jejuni002544 | 1.71 | 218 | TransportDB |
| Bacteria | *Campylobacter* | *jejuni* subsp jejuni81116 | 1.63 | 204 | TransportDB |
| Bacteria | *Campylobacter* | *jejuni* subsp jejuni81176 | 1.7 | 215 | TransportDB |
| Bacteria | *Campylobacter* | *jejuni* subsp jejuniIA3902 | 1.67 | 196 | TransportDB |
| Bacteria | *Campylobacter* | *jejuni* subsp jejuni ICDCCJ07001 | 1.71 | 195 | TransportDB |
| Bacteria | *Campylobacter* | *jejuni* subsp jejuni M1 | 1.62 | 213 | TransportDB |
| Bacteria | *Campylobacter* | *jejuni* subsp jejuni NCTC11168ATCC700819 | 1.64 | 190 | TransportDB |
| Bacteria | *Campylobacter* | *jejuni* subsp jejuni NCTC11168BN148 | 1.64 | 190 | TransportDB |
| Bacteria | *Campylobacter* | *jejuni* subsp jejuni PT14 | 1.64 | 194 | TransportDB |
| Bacteria | *Campylobacter* | *jejuni* subsp jejuniS3 | 1.72 | 210 | TransportDB |
| Bacteria | *Candidatus* | *kinetoplastibacterium blastocrithidiiex strigomonasculicis* | 0.82 | 49 | TransportDB |
| Bacteria | *Candidatus* | *kinetoplastibacterium blastocrithidii* TCC012E | 0.82 | 48 | TransportDB |
| Bacteria | *Candidatus* | *kinetoplastibacterium crithidiiex angomonasdeanei* ATCC30255 | 0.82 | 49 | TransportDB |
| Bacteria | *Candidatus* | *kinetoplastibacterium crithidii* TCC036E | 0.82 | 49 | TransportDB |
| Bacteria | *Candidatus* | *kinetoplastibacterium desouzaii* TCC079E | 0.83 | 48 | TransportDB |
| Bacteria | *Candidatus* | *kinetoplastibacterium galatii* TCC219 | 0.82 | 51 | TransportDB |
| Bacteria | *Candidatus* | *kinetoplastibacterium* oncopeltii TCC290E | 0.81 | 47 | TransportDB |
| Bacteria | *Clostridium* | *kluyveri* DSM555 | 4.02 | 395 | TransportDB |
| Bacteria | *Clostridium* | *kluyveri* NBRC12016 | 3.96 | 383 | TransportDB |
| Bacteria | *Candidatus* | *koribacter versatilis* Ellin345 | 5.65 | 347 | TransportDB |
| Bacteria | *Citrobacter* | *koseri* ATCCBAA895 | 4.74 | 725 | TransportDB |
| Bacteria | *Caldicellulosiruptor* | *kristjanssonii* 177R1B | 2.8 | 224 | TransportDB |
| Bacteria | *Caldicellulosiruptor* | *kronotskyensis* 2002 | 2.84 | 276 | TransportDB |
| Bacteria | *Corynebacterium* | *kroppenstedtii* DSM44385 | 2.45 | 259 | TransportDB |
| Bacteria | *Caldicellulosiruptor* | *lactoaceticus* 6A | 2.67 | 208 | TransportDB |
| Bacteria | *Campylobacter* | *lari* RM2100 | 1.57 | 165 | TransportDB |
| Bacteria | *Clostridium* | *lentocellum* DSM5427 | 4.71 | 499 | TransportDB |
| Bacteria | *Candidatus* | *liberibacter americanus* str SaoPaulo | 1.2 | 83 | TransportDB |
| Bacteria | *Candidatus* | *liberibacter asiaticus* str gxpsy | 1.27 | 84 | TransportDB |
| Bacteria | *Candidatus* | *liberibacter asiaticus* str psy62 | 1.23 | 82 | TransportDB |
| Bacteria | *Candidatus* | *liberibacter solanacearum* CLsoZC1 | 1.26 | 90 | TransportDB |
| Bacteria | *Chlorobium* | *limicola* DSM245 | 2.76 | 181 | TransportDB |
| Bacteria | *Clostridium* | *ljungdahlii* DSM13528 | 4.63 | 519 | TransportDB |
| Bacteria | *Chlorobium* | *luteolum* DSM273 | 2.36 | 183 | TransportDB |
| Bacteria | *Cellulophaga* | *lytica* DSM7489 | 3.77 | 207 | TransportDB |
| Bacteria | *Carnobacterium* | *maltaromaticum* LMA28 | 3.65 | 484 | TransportDB |
| Bacteria | *Cyclobacterium* | *marinum* DSM745 | 6.22 | 312 | TransportDB |
| Bacteria | *Corynebacterium* | *maris* DSM45190 | 2.83 | 307 | TransportDB |
| Bacteria | *Cupriavidus* | *metallidurans* CH34 | 6.91 | 728 | TransportDB |
| Bacteria | *Candidatus* | *Methylomirabilis* oxyfera | 2.75 | 193 | TransportDB |
| Bacteria | *Clavibacter* | *michiganensis* subsp michiganensis NCPPB382 | 3.4 | 395 | TransportDB |
| Bacteria | *Clavibacter* | *michiganensis* subsp nebraskensis NCPPB2581 | 3.06 | 340 | TransportDB |
| Bacteria | *Clavibacter* | *michiganensis* subsp sepedonicus | 3.4 | 356 | TransportDB |
| Bacteria | *Clavibacter* | *midichloria mitochondrii* IricVA | 1.18 | 85 | TransportDB |
| Bacteria | *Chamaesiphon* | *minutus* PCC6605 | 6.76 | 372 | TransportDB |
| Bacteria | *Candidatus* | *moranella endobia* PCIT | 0.54 | 22 | TransportDB |
| Bacteria | *Candidatus* | *moranella endobia* PCVAL | 0.54 | 22 | TransportDB |
| Bacteria | *Chlamydia* | *muridarum* Nigg | 1.08 | 88 | TransportDB |
| Bacteria | *Candidatus* | *mycoplasma haemolamae* str Purdue | 0.76 | 37 | TransportDB |
| Bacteria | *Candidatus* | *mycoplasma haemominutum* Birmingham1 | 0.51 | 31 | TransportDB |
| Bacteria | *Cupriavidus* | *necator* N1 | 8.48 | 944 | TransportDB |
| Bacteria | *Calditerrivibrio* | *nitroreducens* DSM19672 | 2.22 | 233 | TransportDB |
| Bacteria | *Candidatus* | *nitrospira defluvii* | 4.32 | 259 | TransportDB |
| Bacteria | *Clostridium* | *novyi* NT | 2.55 | 274 | TransportDB |
| Bacteria | *Caldicellulosiruptor* | *obsidiansis* OB47 | 2.53 | 230 | TransportDB |
| Bacteria | *Capnocytophaga* | *ochracea* DSM7271 | 2.61 | 140 | TransportDB |
| Bacteria | *Caldicellulosiruptor* | *owensensis* OL | 2.43 | 219 | TransportDB |
| Bacteria | *Chlorobaculum* | *parvum* NCIB8327 | 2.29 | 192 | TransportDB |
| Bacteria | *Clostridium* | *pasteurianum* BC1 | 4.99 | 534 | TransportDB |
| Bacteria | *Chlamydophila* | *pecorum* E58 | 1.11 | 88 | TransportDB |
| Bacteria | *Chlamydia* | *pecorum* P787 | 1.11 | 94 | TransportDB |
| Bacteria | *Chlamydia* | *pecorum* PV30563 | 1.1 | 93 | TransportDB |
| Bacteria | *Chlamydia* | *pecorum* W73 | 1.11 | 93 | TransportDB |
| Bacteria | *Candidatus* | *pelagibacter* sp IMCC9063 | 1.28 | 142 | TransportDB |
| Bacteria | *Candidatus* | *pelagibacter ubique* HTCC1062 | 1.31 | 154 | TransportDB |
| Bacteria | *Clostridium* | *perfringens* ATCC13124 | 3.26 | 358 | TransportDB |
| Bacteria | *Clostridium* | *perfringens* SM101 | 2.96 | 323 | TransportDB |
| Bacteria | *Clostridium* | *perfringens* str 13 | 3.09 | 364 | TransportDB |
| Bacteria | *Chlorobium* | *phaeobacteroides* BS1 | 2.74 | 183 | TransportDB |
| Bacteria | *Chlorobium* | *phaeobacteroides* DSM266 | 3.13 | 177 | TransportDB |
| Bacteria | *Chlorobium* | *phaeovibrioides* DSM265 | 1.97 | 154 | TransportDB |
| Bacteria | *Clostridium* | *phytofermentans* ISDg | 4.85 | 573 | TransportDB |
| Bacteria | *Candidatus* | *phytoplasma aster yellows witches*'-*broom* AY-WB | 0.71 | 55 | TransportDB |
| Bacteria | *Candidatus* | *phytoplasma australiense* | 0.88 | 53 | TransportDB |
| Bacteria | *Candidatus* | *phytoplasma mali* | 0.6 | 48 | TransportDB |
| Bacteria | *Candidatus* | *phytoplasma onion yellows* OY-M | 0.86 | 65 | TransportDB |
| Bacteria | *Candidatus* | *phytoplasma solani* | 0.57 | 59 | TransportDB |
| Bacteria | *Chitinophaga* | *pinensis* DSM2588 | 9.13 | 355 | TransportDB |
| Bacteria | *Chlamydophila* | *pneumoniae* AR39 | 1.23 | 90 | TransportDB |
| Bacteria | *Chlamydophila* | *pneumoniae* CWL029 | 1.23 | 91 | TransportDB |
| Bacteria | *Chlamydophila* | *pneumoniae* J138 | 1.23 | 90 | TransportDB |
| Bacteria | *Chlamydophila* | *pneumoniae* LPCoLN | 1.25 | 88 | TransportDB |
| Bacteria | *Chlamydophila* | *pneumoniae* TW183 | 1.23 | 90 | TransportDB |
| Bacteria | *Candidatus* | *portiera aleyrodidarum* BTBHRs | 0.35 | 20 | TransportDB |
| Bacteria | *Candidatus* | *portiera* aleyrodidarum BTQVLC | 0.35 | 22 | TransportDB |
| Bacteria | *Candidatus* | *portiera aleyrodidarum* TV | 0.28 | 21 | TransportDB |
| Bacteria | *Coprothermobacter* | *proteolyticus* DSM5265 | 1.42 | 171 | TransportDB |
| Bacteria | *Candidatus* | *protochlamydia amoebophila* UWE25 | 2.41 | 151 | TransportDB |
| Bacteria | *Corynebacterium* | *pseudotuberculosis* 1002 | 2.34 | 270 | TransportDB |
| Bacteria | *Corynebacterium* | *pseudotuberculosis* 106A | 2.28 | 254 | TransportDB |
| Bacteria | *Corynebacterium* | *pseudotuberculosis* 258 | 2.31 | 272 | TransportDB |
| Bacteria | *Corynebacterium* | *pseudotuberculosis* 267 | 2.34 | 273 | TransportDB |
| Bacteria | *Corynebacterium* | *pseudotuberculosis* 31 | 2.3 | 269 | TransportDB |
| Bacteria | *Corynebacterium* | *pseudotuberculosis* 316 | 2.31 | 268 | TransportDB |
| Bacteria | *Corynebacterium* | *pseudotuberculosis* 3995 | 2.34 | 270 | TransportDB |
| Bacteria | *Corynebacterium* | *pseudotuberculosis* 4202A | 2.34 | 271 | TransportDB |
| Bacteria | *Corynebacterium* | *pseudotuberculosis* C231 | 2.33 | 261 | TransportDB |
| Bacteria | *Corynebacterium* | *pseudotuberculosis* CIP5297 | 2.32 | 265 | TransportDB |
| Bacteria | *Corynebacterium* | *pseudotuberculosis* Cp162 | 2.29 | 259 | TransportDB |
| Bacteria | *Corynebacterium* | *pseudotuberculosis* FRC41 | 2.34 | 289 | TransportDB |
| Bacteria | *Corynebacterium* | *pseudotuberculosis* I19 | 2.34 | 260 | TransportDB |
| Bacteria | *Corynebacterium* | *pseudotuberculosis* P54B96 | 2.34 | 264 | TransportDB |
| Bacteria | *Corynebacterium* | *pseudotuberculosis* PAT10 | 2.34 | 263 | TransportDB |
| Bacteria | *Chlamydophila* | *psittaci* 01DC11 | 1.17 | 96 | TransportDB |
| Bacteria | *Chlamydia* | *psittaci* 01DC12 | 1.18 | 97 | TransportDB |
| Bacteria | *Chlamydophila* | *psittaci* 02DC15 | 1.17 | 96 | TransportDB |
| Bacteria | *Chlamydophila* | *psittaci* 08DC60 | 1.17 | 97 | TransportDB |
| Bacteria | *Chlamydophila* | *psittaci* 6BC | 1.18 | 96 | TransportDB |
| Bacteria | *Chlamydia* | *psittaci* 8455 | 1.18 | 101 | TransportDB |
| Bacteria | *Chlamydophila* | *psittaci* C1998 | 1.17 | 96 | TransportDB |
| Bacteria | *Chlamydia* | *psittaci* CP3 | 1.18 | 99 | TransportDB |
| Bacteria | *Chlamydia* | *psittaci* GR9 | 1.15 | 95 | TransportDB |
| Bacteria | *Chlamydia* | *psittaci* M56 | 1.17 | 92 | TransportDB |
| Bacteria | *Chlamydophila* | *psittaci* Mat116 | 1.16 | 92 | TransportDB |
| Bacteria | *Chlamydia* | *psittaci* MN | 1.18 | 95 | TransportDB |
| Bacteria | *Chlamydia* | *psittaci* NJ1 | 1.17 | 96 | TransportDB |
| Bacteria | *Chlamydophila* | *psittaci* RD1 | 1.17 | 95 | TransportDB |
| Bacteria | *Chlamydia* | *psittaci* VS225 | 1.16 | 95 | TransportDB |
| Bacteria | *Chlamydia* | *psittaci* WC | 1.18 | 96 | TransportDB |
| Bacteria | *Chlamydia* | *psittaci* WSRTE30 | 1.15 | 98 | TransportDB |
| Bacteria | *Colwellia* | *psychrerythraea* 34H | 5.37 | 407 | TransportDB |
| Bacteria | *Candidatus* | *puniceispirillum marinum* IMCC1322 | 2.75 | 347 | TransportDB |
| Bacteria | *Corynebacterium* | *resistens* DSM45100 | 2.6 | 254 | TransportDB |
| Bacteria | *Candidatus* | *rickettsia* amblyommii str GAT30V | 1.48 | 120 | TransportDB |
| Bacteria | *Candidatus* | *riesia* pediculicola USDA | 0.58 | 31 | TransportDB |
| Bacteria | *Citrobacter* | *rodentium* ICC168 | 5.44 | 672 | TransportDB |
| Bacteria | *Candidatus* | *ruthia magnifica* str Cm Calyptogena magnifica | 1.16 | 57 | TransportDB |
| Bacteria | *Candidatus* | *saccharibacteria* bacterium RAAC3_TM7_1 | 0.85 | 62 | TransportDB |
| Bacteria | *Candidatus* | *saccharimonas aalborgensis* | 1.01 | 66 | TransportDB |
| Bacteria | *Clostridium* | *saccharobutylicum* DSM13864 | 5.11 | 494 | TransportDB |
| Bacteria | *Clostridium* | *saccharolyticum* WM1 | 4.66 | 645 | TransportDB |
| Bacteria | *Caldicellulosiruptor* | *saccharolyticus* DSM8903 | 2.97 | 286 | TransportDB |
| Bacteria | *Clostridium* | *saccharoperbutylacetonicum* N14HMT | 6.67 | 694 | TransportDB |
| Bacteria | *Cronobacter* | *sakazakii* ATCCBAA894 | 4.53 | 567 | TransportDB |
| Bacteria | *Cronobacter* | *sakazakii* CMCC45402 | 4.56 | 570 | TransportDB |
| Bacteria | *Cronobacter* | *sakazakii* ES15 | 4.27 | 548 | TransportDB |
| Bacteria | *Cronobacter* | *sakazakii* SP291 | 4.52 | 559 | TransportDB |
| Bacteria | *Chromohalobacter* | *salexigens* DSM3043 | 3.7 | 514 | TransportDB |
| Bacteria | *Caulobacter* | *segnis* ATCC21756 | 4.66 | 298 | TransportDB |
| Bacteria | *Candidatus* | *solibacter usitatus* Ellin6076 | 9.97 | 526 | TransportDB |
| Bacteria | *Carnobacterium* | *s174* | 2.69 | 406 | TransportDB |
| Bacteria | *Coprococcus* | *sART551* | 3.12 | 227 | TransportDB |
| Bacteria | *Cyanothece* | *sATCC51142* | 5.46 | 383 | TransportDB |
| Bacteria | *Chelativorans* | *sBNC1* | 4.94 | 640 | TransportDB |
| Bacteria | *Clostridium* | *sBNL1100* | 4.61 | 410 | TransportDB |
| Bacteria | *Caulobacter* | *sK31* | 5.89 | 375 | TransportDB |
| Bacteria | *Cycloclasticus* | *sP1* | 2.36 | 221 | TransportDB |
| Bacteria | *Calothrix* | *sPCC6303* | 6.96 | 367 | TransportDB |
| Bacteria | *Cyanothece* | *sPCC7424* | 6.55 | 371 | TransportDB |
| Bacteria | *Cyanothece* | *sPCC7425* | 5.79 | 385 | TransportDB |
| Bacteria | *Calothrix* | *sPCC7507* | 7.02 | 436 | TransportDB |
| Bacteria | *Cyanothece* | *sPCC7822* | 7.84 | 408 | TransportDB |
| Bacteria | *Cyanothece* | *sPCC8801* | 4.79 | 296 | TransportDB |
| Bacteria | *Clostridium* | *sSY8519* | 2.84 | 255 | TransportDB |
| Bacteria | *Carnobacterium* | *sWN1359* | 2.5 | 329 | TransportDB |
| Bacteria | *Chloroflexus* | *sY400fl* | 5.27 | 397 | TransportDB |
| Bacteria | *Cylindrospermum* | *stagnale* PCC7417 | 7.61 | 374 | TransportDB |
| Bacteria | *Cyanobacterium* | *stanieri* PCC7202 | 3.16 | 239 | TransportDB |
| Bacteria | *Clostridium* | *stercorarium* subsp stercorarium DSM8532 | 2.97 | 363 | TransportDB |
| Bacteria | *Clostridium* | *sticklandii* DSM519 | 2.72 | 310 | TransportDB |
| Bacteria | *Candidatus* | *symbiobacter mobilis* CR | 2.99 | 157 | TransportDB |
| Bacteria | *Cupriavidus* | *taiwanensis* LMG19424 | 3.42 | 724 | TransportDB |
| Bacteria | *Cupriavidus* | *taiwanensis* LMG19424 | 6.48 | 700 | TransportDB |
| Bacteria | *Chlorobium* | *tepidum* TLS | 2.15 | 168 | TransportDB |
| Bacteria | *Corynebacterium* | *terpenotabidum* Y11 | 2.75 | 251 | TransportDB |
| Bacteria | *Comamonas* | *testosteroni* CNB2 | 5.37 | 645 | TransportDB |
| Bacteria | *Clostridium* | *tetani* 12124569 | 2.87 | 327 | TransportDB |
| Bacteria | *Clostridium* | *tetani* E88 | 2.87 | 313 | TransportDB |
| Bacteria | *Chloroherpeton* | *thalassium* ATCC35110 | 3.29 | 211 | TransportDB |
| Bacteria | *Chroococcidiopsis* | *thermalis* PCC7203 | 6.69 | 511 | TransportDB |
| Bacteria | *Clostridium* | *thermocellum* ATCC27405 | 3.84 | 243 | TransportDB |
| Bacteria | *Clostridium* | *thermocellum* DSM1313 | 3.56 | 237 | TransportDB |
| Bacteria | *Chlamydia* | *trachomatis* | 10.43 | 888 | TransportDB |
| Bacteria | *Chlamydia* | *trachomatis* 434Bu | 1.04 | 89 | TransportDB |
| Bacteria | *Chlamydia* | *trachomatis* A2497 | 1.05 | 90 | TransportDB |
| Bacteria | *Chlamydia* | *trachomatis* A363 | 1.05 | 90 | TransportDB |
| Bacteria | *Chlamydia* | *trachomatis* A5291 | 1.05 | 90 | TransportDB |
| Bacteria | *Chlamydia* | *trachomatis* AHAR13 | 1.05 | 92 | TransportDB |
| Bacteria | *Chlamydia* | *trachomatis* BJali20OT | 1.04 | 87 | TransportDB |
| Bacteria | *Chlamydia* | *trachomatis* BTZ1A828OT | 1.04 | 87 | TransportDB |
| Bacteria | *Chlamydia* | *trachomatis* CTW3 | 1.05 | 88 | TransportDB |
| Bacteria | *Chlamydia* | *trachomatis* DEC | 1.05 | 88 | TransportDB |
| Bacteria | *Chlamydia* | *trachomatis* RCJ971 | 1.04 | 89 | TransportDB |
| Bacteria | *Chlamydia* | *trachomatis* RCJs122 | 1.06 | 90 | TransportDB |
| Bacteria | *Chlamydia* | *trachomatis* RCL255 | 1.04 | 89 | TransportDB |
| Bacteria | *Chlamydia* | *trachomatis* RCL2s3 | 1.05 | 90 | TransportDB |
| Bacteria | *Chlamydia* | *trachomatis* RCL2s46 | 1.04 | 89 | TransportDB |
| Bacteria | *Chlamydia* | *trachomatis* Sweden2 | 1.04 | 88 | TransportDB |
| Bacteria | *Cronobacter* | *turicensis* z3032 | 4.6 | 582 | TransportDB |
| Bacteria | *Corynebacterium* | *ulcerans* 0102 | 2.58 | 318 | TransportDB |
| Bacteria | *Corynebacterium* | *ulcerans* 809 | 2.5 | 319 | TransportDB |
| Bacteria | *Corynebacterium* | *ulcerans* BRAD22 | 2.61 | 317 | TransportDB |
| Bacteria | *Corynebacterium* | *urealyticum* DSM7109 | 2.37 | 231 | TransportDB |
| Bacteria | *Corynebacterium* | *urealyticum* DSM7111 | 2.32 | 229 | TransportDB |
| Bacteria | *Corynebacterium* | *variabile* DSM44702 | 3.43 | 363 | TransportDB |
| Bacteria | *Candidatus* | *vesicomyosocius okutanii* HA | 1.02 | 54 | TransportDB |
| Bacteria | *Chromobacterium* | *violaceum* ATCC12472 | 4.75 | 481 | TransportDB |
| Bacteria | *Conexibacter* | *woesei* DSM14684 | 6.36 | 772 | TransportDB |
| Bacteria | *Cycloclasticus* | *zancles* 7ME | 2.66 | 249 | TransportDB |
| Bacteria | *Denitrovibrio* | *acetiphilus* DSM12809 | 3.22 | 316 | TransportDB |
| Bacteria | *Desulfobacca* | *acetoxidans* DSM11109 | 3.28 | 255 | TransportDB |
| Bacteria | *Desulfotomaculum* | *acetoxidans* DSM771 | 4.55 | 308 | TransportDB |
| Bacteria | *Desulfosporosinus* | *acidiphilus* SJ4 | 4.99 | 424 | TransportDB |
| Bacteria | *Delftia* | *acidovorans* SPH1 | 6.77 | 797 | TransportDB |
| Bacteria | *Desulfovibrio* | *aespoeensis* Aspo2 | 3.63 | 368 | TransportDB |
| Bacteria | *Desulfovibrio* | *africanus* str WalvisBay | 4.2 | 391 | TransportDB |
| Bacteria | *Desulfovibrio* | *alaskensis* G20 | 3.73 | 389 | TransportDB |
| Bacteria | *Desulfurivibrio* | *alkaliphilus* AHT2 | 3.1 | 251 | TransportDB |
| Bacteria | *Desulfatibacillum* | *alkenivorans* AK01 | 6.52 | 469 | TransportDB |
| Bacteria | *Dechloromonas* | *aromatica* RCB | 4.5 | 406 | TransportDB |
| Bacteria | *Desulfobacterium* | *autotrophicum* HRM2 | 5.66 | 667 | TransportDB |
| Bacteria | *Desulfomicrobium* | *baculatum* DSM4028 | 3.94 | 423 | TransportDB |
| Bacteria | *Desulfarculus* | *baarsii* DSM2075 | 3.66 | 277 | TransportDB |
| Bacteria | *Desulfotomaculum* | *carboxydivorans* CO1SRB | 2.89 | 236 | TransportDB |
| Bacteria | *Dickeya* | *dadantii* 3937 | 4.92 | 692 | TransportDB |
| Bacteria | *Dickeya* | *dadantii* Ech586 | 4.82 | 642 | TransportDB |
| Bacteria | *Dickeya* | *dadantii* Ech703 | 4.68 | 679 | TransportDB |
| Bacteria | *Desulfitobacterium* | *dehalogenans* ATCC51507 | 4.32 | 415 | TransportDB |
| Bacteria | *Deinococcus* | *deserti* VCD115 | 3.86 | 370 | TransportDB |
| Bacteria | *Desulfovibrio* | *desulfuricans* G20 | 3.73 | 355 | TransportDB |
| Bacteria | *Desulfovibrio* | *desulfuricans* ND132 | 3.86 | 459 | TransportDB |
| Bacteria | *Deferribacter* | *desulfuricans* SSM1 | 2.54 | 247 | TransportDB |
| Bacteria | *Deferribacter* | *desulfuricans* subsp desulfuricans str ATCC27774 | 2.87 | 268 | TransportDB |
| Bacteria | *Desulfitobacterium* | *dichloroeliminans* LMGP21439 | 3.62 | 351 | TransportDB |
| Bacteria | *Dehalococcoides* | *ethenogenes* 195 | 1.47 | 115 | TransportDB |
| Bacteria | *Dyadobacter* | *fermentans* DSM18053 | 6.97 | 320 | TransportDB |
| Bacteria | *Deinococcus* | *geothermalis* DSM11300 | 3.25 | 337 | TransportDB |
| Bacteria | *Desulfotomaculum* | *gibsoniae* DSM7213 | 4.86 | 321 | TransportDB |
| Bacteria | *Desulfovibrio* | *gigas* DSM1382ATCC19364 | 3.80 | 331 | TransportDB |
| Bacteria | *Deinococcus* | *gobiensis* I0 | 4.41 | 403 | TransportDB |
| Bacteria | *Desulfitobacterium* | *hafniense* DCB2 | 5.28 | 619 | TransportDB |
| Bacteria | *Desulfitobacterium* | *hafniense* Y51 | 5.73 | 660 | TransportDB |
| Bacteria | *Desulfovibrio* | *hydrothermalis* AM13DSM14728 | 3.71 | 387 | TransportDB |
| Bacteria | *Desulfurispirillum* | *indicum* S5 | 2.93 | 271 | TransportDB |
| Bacteria | *Desulfotomaculum* | *kuznetsovii* DSM6115 | 3.6 | 269 | TransportDB |
| Bacteria | *Dehalogenimonas* | *lykanthroporepellens* BLDC9 | 1.69 | 124 | TransportDB |
| Bacteria | *Desulfovibrio* | *magneticus* RS1 | 5.32 | 407 | TransportDB |
| Bacteria | *Deinococcus* | *maricopensis* DSM21211 | 3.5 | 303 | TransportDB |
| Bacteria | *Dehalococcoides* | *mccartyi* BTF08 | 1.45 | 117 | TransportDB |
| Bacteria | *Dehalococcoides* | *mccartyi* DCMB5 | 1.43 | 119 | TransportDB |
| Bacteria | *Dehalococcoides* | *mccartyi* GY50 | 1.41 | 116 | TransportDB |
| Bacteria | *Desulfosporosinus* | *meridiei* DSM13257 | 4.87 | 499 | TransportDB |
| Bacteria | *Dichelobacter* | *nodosus* VCS1703A | 1.39 | 127 | TransportDB |
| Bacteria | *Desulfococcus* | *oleovorans* Hxd3 | 3.94 | 238 | TransportDB |
| Bacteria | *Desulfosporosinus* | *orientis* DSM765 | 5.86 | 572 | TransportDB |
| Bacteria | *Deinococcus* | *peraridilitoris* DSM19664 | 4.51 | 392 | TransportDB |
| Bacteria | *Desulfovibrio* | *piezophilus* C1TLV30 | 3.65 | 382 | TransportDB |
| Bacteria | *Desulfobulbus* | *propionicus* DSM2032 | 3.85 | 309 | TransportDB |
| Bacteria | *Deinococcus* | *proteolyticus* MRP | 2.89 | 223 | TransportDB |
| Bacteria | *Desulfotalea* | *psychrophila* LSv54 | 3.66 | 370 | TransportDB |
| Bacteria | *Deinococcus* | *radiodurans* R1 | 3.28 | 283 | TransportDB |
| Bacteria | *Desulfotomaculum* | *reducens* MI1 | 3.61 | 329 | TransportDB |
| Bacteria | *Desulfohalobium* | *retbaense* DSM5692 | 2.91 | 297 | TransportDB |
| Bacteria | *Desulfotomaculum* | *ruminis* DSM2154 | 3.97 | 396 | TransportDB |
| Bacteria | *Desulfovibrio* | *salexigens* DSM2638 | 4.29 | 502 | TransportDB |
| Bacteria | *Dactylococcopsis* | *salina* PCC8305 | 3.78 | 265 | TransportDB |
| Bacteria | *Dinoroseobacter* | *shibae* DFL12 | 4.42 | 564 | TransportDB |
| Bacteria | *Dehalococcoides* | *sBAV1* | 1.34 | 113 | TransportDB |
| Bacteria | *Dehalococcoides* | *sCBDB1* | 1.4 | 121 | TransportDB |
| Bacteria | *Dehalobacter* | *sCF* | 3.09 | 222 | TransportDB |
| Bacteria | *Delftia* | *sCs14* | 6.69 | 813 | TransportDB |
| Bacteria | *Dehalobacter* | *sDCA* | 3.07 | 227 | TransportDB |
| Bacteria | *Dehalococcoides* | *sGT* | 1.36 | 114 | TransportDB |
| Bacteria | *Dehalococcoides* | *sVS* | 1.41 | 119 | TransportDB |
| Bacteria | *Dechlorosoma* | *suillum* PS | 3.81 | 329 | TransportDB |
| Bacteria | *Desulfocapsa* | *sulfexigens* DSM10523 | 4.02 | 402 | TransportDB |
| Bacteria | *Desulfurobacterium* | *thermolithotrophum* DSM11699 | 1.54 | 115 | TransportDB |
| Bacteria | *Dictyoglomus* | *thermophilum* H612 | 1.96 | 284 | TransportDB |
| Bacteria | *Desulfomonile* | *tiedjei* DSM6799 | 6.53 | 541 | TransportDB |
| Bacteria | *Desulfobacula* | *toluolica* Tol2 | 5.2 | 431 | TransportDB |
| Bacteria | *Dictyoglomus* | *turgidum* DSM6724 | 1.86 | 254 | TransportDB |
| Bacteria | *Desulfovibrio* | *vulgaris* DP4 | 3.66 | 352 | TransportDB |
| Bacteria | *Desulfovibrio* | *vulgaris* RCH1 | 3.73 | 348 | TransportDB |
| Bacteria | *Desulfovibrio* | *vulgaris* str Hildenborough | 3.77 | 343 | TransportDB |
| Bacteria | *Desulfovibrio* | *vulgaris* str MiyazakiF | 4.04 | 372 | TransportDB |
| Bacteria | *Dickeya* | *zeae* Ech1591 | 4.81 | 629 | TransportDB |
| Bacteria | *Bordetella* | *Eubacterium* T287 | 1.97 | 163 | TransportDB |
| Bacteria | *Enterobacter* | *aerogenes* EA1509E | 5.59 | 790 | TransportDB |
| Bacteria | *Enterobacter* | *aerogenes* KCTC2190 | 5.28 | 771 | TransportDB |
| Bacteria | *Erwinia* | *amylovora* ATCC49946 | 3.91 | 441 | TransportDB |
| Bacteria | *Erwinia* | *amylovora* CFBP1430 | 3.83 | 446 | TransportDB |
| Bacteria | *Exiguobacterium* | *antarcticum* B7 | 2.82 | 347 | TransportDB |
| Bacteria | *Enterobacter* | *asburiae* LF7a | 5.01 | 758 | TransportDB |
| Bacteria | *Enterobacter* | *bacterium* str FGI57 | 4.76 | 689 | TransportDB |
| Bacteria | *Erwinia* | *billingiae* Eb661 | 5.37 | 805 | TransportDB |
| Bacteria | *Ehrlichia* | *canis* str Jake | 1.32 | 74 | TransportDB |
| Bacteria | *Enterococcus* | *casseliflavus* EC20 | 3.43 | 564 | TransportDB |
| Bacteria | *Ehrlichia* | *chaffeensis* str Arkansas | 1.18 | 73 | TransportDB |
| Bacteria | *Enterobacter* | *cloacae* EcWSU1 | 4.8 | 704 | TransportDB |
| Bacteria | *Enterobacter* | *cloacae* SCF1 | 4.81 | 736 | TransportDB |
| Bacteria | *Enterobacter* | *cloacae* subsp cloacae ATCC13047 | 5.6 | 736 | TransportDB |
| Bacteria | *Enterobacter* | *cloacae* subsp cloacae ENHKU01 | 4.73 | 668 | TransportDB |
| Bacteria | *Enterobacter* | *cloacae* subsp cloacae NCTC9394 | 4.91 | 574 | TransportDB |
| Bacteria | *Enterobacter* | *cloacae* subsp dissolvens SDM | 4.97 | 719 | TransportDB |
| Bacteria | *Escherichia* | *coli* 042 | 5.36 | 637 | TransportDB |
| Bacteria | *Escherichia* | *coli* 536 | 4.94 | 659 | TransportDB |
| Bacteria | *Escherichia* | *coli* 55989 | 5.15 | 631 | TransportDB |
| Bacteria | *Escherichia* | *coli* ABU83972 | 5.13 | 672 | TransportDB |
| Bacteria | *Escherichia* | *coli* APECO1 | 5.5 | 668 | TransportDB |
| Bacteria | *Escherichia* | *coli* APECO78 | 4.8 | 635 | TransportDB |
| Bacteria | *Escherichia* | *coli* ATCC8739 | 4.75 | 615 | TransportDB |
| Bacteria | *Escherichia* | *coli* BL21 DE3 | 4.56 | 604 | TransportDB |
| Bacteria | *Escherichia* | *coli* BL21 Gold DE3pLysSAG | 4.57 | 605 | TransportDB |
| Bacteria | *Escherichia* | *coli* B str REL606 | 4.63 | 605 | TransportDB |
| Bacteria | *Escherichia* | *coli* BW2952 | 4.58 | 592 | TransportDB |
| Bacteria | *Escherichia* | *coli* CFT073 | 5.23 | 664 | TransportDB |
| Bacteria | *Escherichia* | *coli* DH1 | 4.62 | 614 | TransportDB |
| Bacteria | *Escherichia* | *coli* E24377A | 5.25 | 623 | TransportDB |
| Bacteria | *Escherichia* | *coli* ED1a | 5.21 | 648 | TransportDB |
| Bacteria | *Escherichia* | *coli* ETECH10407 | 5.33 | 616 | TransportDB |
| Bacteria | *Escherichia* | *coli* HS | 4.64 | 603 | TransportDB |
| Bacteria | *Escherichia* | *coli* IAI1 | 4.7 | 615 | TransportDB |
| Bacteria | *Escherichia* | *coli* IAI39 | 5.13 | 648 | TransportDB |
| Bacteria | *Escherichia* | *coli* IHE3034 | 5.11 | 652 | TransportDB |
| Bacteria | *Escherichia* | *coli* JJ1886 | 5.31 | 696 | TransportDB |
| Bacteria | *Escherichia* | *coli* KO11FL | 5.03 | 620 | TransportDB |
| Bacteria | *Escherichia* | *coli* LF82 | 4.77 | 650 | TransportDB |
| Bacteria | *Escherichia* | *coli* LY180 | 4.84 | 607 | TransportDB |
| Bacteria | *Escherichia* | *coli* NA114 | 4.97 | 675 | TransportDB |
| Bacteria | *Escherichia* | *coli* O103 | 5.52 | 621 | TransportDB |
| Bacteria | *Escherichia* | *coli* O104 | 5.39 | 637 | TransportDB |
| Bacteria | *Escherichia* | *coli* O104 | 5.44 | 644 | TransportDB |
| Bacteria | *Escherichia* | *coli* O104 | 5.44 | 643 | TransportDB |
| Bacteria | *Escherichia* | *coli* O111 | 5.77 | 617 | TransportDB |
| Bacteria | *Escherichia* | *coli* O127 | 5.07 | 620 | TransportDB |
| Bacteria | *Escherichia* | *coli* SMS35 | 5.22 | 673 | TransportDB |
| Bacteria | *Escherichia* | *coli* str clone Di14 | 5.04 | 662 | TransportDB |
| Bacteria | *Escherichia* | *coli str clone Di2* | 5.04 | 662 | TransportDB |
| Bacteria | *Escherichia* | *coli* str K12 substr DH10B | 4.69 | 600 | TransportDB |
| Bacteria | *Escherichia* | *coli* str K12 substr MDS42 | 3.98 | 547 | TransportDB |
| Bacteria | *Escherichia* | *coli* str K12 substr MG1655 | 4.64 | 601 | TransportDB |
| Bacteria | *Escherichia* | *coli* str K12 substr W3110 | 4.65 | 608 | TransportDB |
| Bacteria | *Escherichia* | *coli* UM146 | 5.11 | 668 | TransportDB |
| Bacteria | *Escherichia* | *coli* UMN026 | 5.36 | 677 | TransportDB |
| Bacteria | *Escherichia* | *coli* UMNK88 | 5.67 | 645 | TransportDB |
| Bacteria | *Escherichia* | *coli* UTI89 | 5.18 | 665 | TransportDB |
| Bacteria | *Escherichia* | *coli* W | 5.01 | 620 | TransportDB |
| Bacteria | *Escherichia* | *coli* Xuzhou21 | 5.52 | 644 | TransportDB |
| Bacteria | *Eubacterium* | *eligens* ATCC27750 | 2.83 | 270 | TransportDB |
| Bacteria | *Enterococcus* | *faecalis* 62 | 3.13 | 465 | TransportDB |
| Bacteria | *Enterococcus* | *faecalis* D32 | 3.06 | 426 | TransportDB |
| Bacteria | *Enterococcus* | *faecalis* OG1RF | 2.74 | 429 | TransportDB |
| Bacteria | *Enterococcus* | *faecalis* str Symbioflor1 | 2.81 | 436 | TransportDB |
| Bacteria | *Enterococcus* | *faecalis* V583 | 3.36 | 459 | TransportDB |
| Bacteria | *Enterococcus* | *faecium* Aus0004 | 3.02 | 403 | TransportDB |
| Bacteria | *Escherichia* | *fergusonii* ATCC35469 | 4.64 | 585 | TransportDB |
| Bacteria | *Ethanoligenens* | *harbinense* YUAN3 | 3.01 | 292 | TransportDB |
| Bacteria | *Enterococcus* | *hirae* ATCC9790 | 2.86 | 358 | TransportDB |
| Bacteria | *Edwardsiella* | *ictaluri* 93146 | 3.81 | 428 | TransportDB |
| Bacteria | *Eggerthella* | *lenta* DSM2243 | 3.63 | 359 | TransportDB |
| Bacteria | *Eubacterium* | *limosum* KIST612 | 4.32 | 499 | TransportDB |
| Bacteria | *Erythrobacter* | *litoralis* HTCC2594 | 3.05 | 181 | TransportDB |
| Bacteria | *Elusimicrobium* | *minutum* Pei191 | 1.64 | 113 | TransportDB |
| Bacteria | *Enterococcus* | *mundtii* QU25 | 3.35 | 428 | TransportDB |
| Bacteria | *Ehrlichia* | *muris* AS145 | 1.2 | 73 | TransportDB |
| Bacteria | *Emticicia* | *oligotrophica* DSM17448 | 5.22 | 258 | TransportDB |
| Bacteria | *Erwinia* | *pyrifoliae* DSM12163 | 4.07 | 458 | TransportDB |
| Bacteria | *Erwinia* | *pyrifoliae* Ep196 | 4.07 | 442 | TransportDB |
| Bacteria | *Eubacterium* | *rectale* ATCC33656 | 3.45 | 320 | TransportDB |
| Bacteria | *Eubacterium* | *rectale* DSM17629 | 3.34 | 279 | TransportDB |
| Bacteria | *Eubacterium* | *rectale* M1041 | 3.7 | 279 | TransportDB |
| Bacteria | *Erysipelothrix* | *rhusiopathiae* str Fujisawa | 1.79 | 247 | TransportDB |
| Bacteria | *Erysipelothrix* | *rhusiopathiae* SY1027 | 1.75 | 249 | TransportDB |
| Bacteria | *Ehrlichia* | *ruminantium* str Gardel | 1.5 | 72 | TransportDB |
| Bacteria | *Ehrlichia* | *ruminantium* str Welgevonden | 1.51 | 74 | TransportDB |
| Bacteria | *Enterobacter* | *sakazakii* ATCC BAA-894 | 4.37 | 549 | TransportDB |
| Bacteria | *Arthrospira* | *bacteriumphylo* type RsD17 | 2.3 | 46 | TransportDB |
| Bacteria | *Sulfuricurvum* | *RIFRC1* | 2.36 | 161 | TransportDB |
| Bacteria | *Exiguobacterium* | *sibiricum* 25515 | 3.04 | 380 | TransportDB |
| Bacteria | *Eubacterium* | *siraeum* 703 | 2.94 | 210 | TransportDB |
| Bacteria | *Eubacterium* | *siraeum* V10Sc8a | 2.84 | 209 | TransportDB |
| Bacteria | *Enterobacter* | *s638* | 4.68 | 662 | TransportDB |
| Bacteria | *Enterococcus* | *s7L76* | 3.1 | 363 | TransportDB |
| Bacteria | *Exiguobacterium* | *sAT1b* | 3.0 | 387 | TransportDB |
| Bacteria | *Erwinia* | *sEjp617* | 3.96 | 456 | TransportDB |
| Bacteria | *Exiguobacterium* | *sMH3* | 3.16 | 413 | TransportDB |
| Bacteria | *Enterobacter* | *sR4368* | 5.16 | 695 | TransportDB |
| Bacteria | *Eggerthella* | *sYY7918* | 3.12 | 282 | TransportDB |
| Bacteria | *Edwardsiella* | *tarda* C07087 | 3.86 | 473 | TransportDB |
| Bacteria | *Edwardsiella* | *tarda* EIB202 | 3.8 | 470 | TransportDB |
| Bacteria | *Edwardsiella* | *tarda* FL660 | 3.73 | 466 | TransportDB |
| Bacteria | *Erwinia* | *tasmaniensis* Et199 | 4.07 | 503 | TransportDB |
| Bacteria | *Echinicola* | *vietnamensis* DSM17526 | 5.61 | 275 | TransportDB |
| Bacteria | *Fibrella* | *aestuarina* BUZ2 | 6.94 | 326 | TransportDB |
| Bacteria | *Frankia* | *alni* ACN14a | 7.5 | 433 | TransportDB |
| Bacteria | *Filifactor* | *alocis* ATCC35896 | 1.93 | 179 | TransportDB |
| Bacteria | *Frateuria* | *aurantia* DSM6220 | 3.6 | 270 | TransportDB |
| Bacteria | *Flavobacteriaceae* | *bacterium* 351910 | 2.77 | 142 | TransportDB |
| Bacteria | *Ferrimonas* | *balearica DSM9799* | 4.28 | 379 | TransportDB |
| Bacteria | *Flavobacterium* | *branchiophilum* FL15 | 3.56 | 147 | TransportDB |
| Bacteria | *Francisella* | *cnovicida* 3523 | 1.95 | 229 | TransportDB |
| Bacteria | *Francisella* | *cnovicida* Fx1 | 1.91 | 232 | TransportDB |
| Bacteria | *Flavobacterium* | *columnare* ATCC49512 | 3.16 | 152 | TransportDB |
| Bacteria | *Flavobacterium* | *indicum* GPTSA1009 | 2.99 | 161 | TransportDB |
| Bacteria | *Flavobacterium* | *johnsoniae* UW101 | 6.1 | 281 | TransportDB |
| Bacteria | *Flexibacter* | *litoralis* DSM6794 | 4.92 | 170 | TransportDB |
| Bacteria | *Finegoldia* | *magna* ATCC29328 | 1.99 | 257 | TransportDB |
| Bacteria | *Francisella* | *noatunensis* subsp orientalis LADL07285A | 1.86 | 183 | TransportDB |
| Bacteria | *Francisella* | *noatunensis* subsp orientalis str Toba04 | 1.85 | 179 | TransportDB |
| Bacteria | *Fervidobacterium* | *nodosum* Rt17B1 | 1.95 | 224 | TransportDB |
| Bacteria | *Francisella* | *novicida* U112 | 1.91 | 225 | TransportDB |
| Bacteria | *Fusobacterium* | *nucleatum* subsp animalis4_8 | 2.28 | 273 | TransportDB |
| Bacteria | *Fusobacterium* | *nucleatum* subsp nucleatum ATCC25586 | 2.17 | 286 | TransportDB |
| Bacteria | *Fusobacterium* | *nucleatum* subsp vincentii3_1_36A2 | 2.27 | 281 | TransportDB |
| Bacteria | *Fervidobacterium* | *pennivorans* DSM9078 | 2.17 | 256 | TransportDB |
| Bacteria | *Francisella* | *philomiragia* subsp philomiragia ATCC25017 | 2.05 | 236 | TransportDB |
| Bacteria | *Faecalibacterium* | *prausnitzii* L26 | 3.32 | 316 | TransportDB |
| Bacteria | *Faecalibacterium* | *prausnitzii* SL33 | 3.21 | 262 | TransportDB |
| Bacteria | *Flavobacterium* | *psychrophilum* JIP0286 | 2.86 | 152 | TransportDB |
| Bacteria | *Flexistipes* | *sinusarabici* DSM4947 | 2.53 | 274 | TransportDB |
| Bacteria | *Frankia* | *sCcI3* | 5.43 | 253 | TransportDB |
| Bacteria | *Frankia* | *sEAN1pec* | 8.98 | 507 | TransportDB |
| Bacteria | *Frankia* | *sEuI1c* | 8.82 | 599 | TransportDB |
| Bacteria | *Francisella* | *sTX077308* | 2.04 | 244 | TransportDB |
| Bacteria | *Fibrobacter* | *succinogenes* subsp succinogenes S85 | 3.84 | 194 | TransportDB |
| Bacteria | *Frankia* | *symbiont* of datiscaglomerata | 5.34 | 300 | TransportDB |
| Bacteria | *Fluviicola* | *taffensis* DSM16823 | 4.63 | 184 | TransportDB |
| Bacteria | *Francisella* | *tularensis novicida* U112 | 1.91 | 216 | TransportDB |
| Bacteria | *Francisella* | *tularensis* subsp holarctica F92 | 1.89 | 203 | TransportDB |
| Bacteria | *Francisella* | *tularensis* subsp holarctica FSC200 | 1.89 | 168 | TransportDB |
| Bacteria | *Francisella* | *tularensis* subsp holarctica FTNF00200 | 1.89 | 167 | TransportDB |
| Bacteria | *Francisella* | *tularensis* subsp holarctica LVS | 1.9 | 210 | TransportDB |
| Bacteria | *Francisella* | *tularensis* subsp holarctica OSU18 | 1.9 | 162 | TransportDB |
| Bacteria | *Francisella* | *tularensis* subsp mediasiatica FSC147 | 1.89 | 171 | TransportDB |
| Bacteria | *Francisella* | *tularensis* subsp tularensis FSC198 | 1.89 | 182 | TransportDB |
| Bacteria | *Francisella* | *tularensis* subsp tularensis NE061598 | 1.89 | 192 | TransportDB |
| Bacteria | *Francisella* | *tularensis* subsp tularensis SCHUS4 | 1.89 | 182 | TransportDB |
| Bacteria | *Francisella* | *tularensis* subsp tularensis TI0902 | 1.89 | 184 | TransportDB |
| Bacteria | *Francisella* | *tularensis* subsp tularensis TIGB03 | 1.97 | 187 | TransportDB |
| Bacteria | *Francisella* | *tularensis* subsp tularensis WY963418 | 1.9 | 190 | TransportDB |
| Bacteria | *Gallibacterium* | *anatis* UMN179 | 2.69 | 314 | TransportDB |
| Bacteria | *Gemmatimonas* | *aurantiaca* T27 | 4.64 | 286 | TransportDB |
| Bacteria | *Geobacter* | *bemidjiensis* Bem | 4.62 | 337 | TransportDB |
| Bacteria | *Granulibacter* | *bethesdensis* CGDNIH1 | 2.71 | 225 | TransportDB |
| Bacteria | *Gordonia* | *bronchialis* DSM43247 | 5.29 | 398 | TransportDB |
| Bacteria | *Gallionella* | *capsiferriformans* ES2 | 3.16 | 224 | TransportDB |
| Bacteria | *Geobacter* | *daltonii* FRC32 | 4.30 | 309 | TransportDB |
| Bacteria | *Gluconacetobacter* | *diazotrophicus* PAl5 | 4.0 | 363 | TransportDB |
| Bacteria | *Gramella* | *forsetii* KT0803 | 3.8 | 228 | TransportDB |
| Bacteria | *Gammaproteobacteria* | *gamma* HdN1 | 4.59 | 236 | TransportDB |
| Bacteria | *Geobacillus* | *kaustophilus* HTA426 | 3.59 | 378 | TransportDB |
| Bacteria | *Gloeobacter* | *kilaueensis* JS1 | 4.72 | 294 | TransportDB |
| Bacteria | *Geobacter* | *lovleyi* SZ | 3.99 | 306 | TransportDB |
| Bacteria | *Granulicella* | *mallensis* MP5ACTX8 | 6.24 | 292 | TransportDB |
| Bacteria | *Geobacter* | *metallireducens* GS15 | 4.01 | 265 | TransportDB |
| Bacteria | *Glaciecola* | *nitratireducens* FR1064 | 4.13 | 308 | TransportDB |
| Bacteria | *Geodermatophilus* | *obscurus* DSM43160 | 5.32 | 394 | TransportDB |
| Bacteria | *Gluconobacter* | *oxydans* 621H | 2.92 | 254 | TransportDB |
| Bacteria | *Gluconobacter* | *oxydans* H24 | 3.82 | 329 | TransportDB |
| Bacteria | *Gordonibacter* | *pamelaeae* 7101b | 3.61 | 228 | TransportDB |
| Bacteria | *Gordonia* | *polyisoprenivorans* VH2 | 5.84 | 512 | TransportDB |
| Bacteria | *Glaciecola* | *psychrophila* 170 | 5.41 | 398 | TransportDB |
| Bacteria | *Glaciecola* | *s4H37YE5* | 5.39 | 371 | TransportDB |
| Bacteria | *Geobacillus* | *sC56T3* | 3.65 | 403 | TransportDB |
| Bacteria | *Geobacillus* | *sGHH01* | 3.58 | 401 | TransportDB |
| Bacteria | *Geobacillus* | *sJF8* | 3.49 | 403 | TransportDB |
| Bacteria | *Gordonia* | *sKTR9* | 5.89 | 478 | TransportDB |
| Bacteria | *Geobacter* | *sM18* | 5.28 | 367 | TransportDB |
| Bacteria | *Geobacter* | *sM21* | 4.75 | 314 | TransportDB |
| Bacteria | *Geitlerinema* | *sPCC7407* | 4.68 | 304 | TransportDB |
| Bacteria | *Gloeocapsa* | *sPCC7428* | 5.88 | 487 | TransportDB |
| Bacteria | *Geobacillus* | *sWCH70* | 3.51 | 334 | TransportDB |
| Bacteria | *Geobacillus* | *sY412MC52* | 3.67 | 387 | TransportDB |
| Bacteria | *Geobacillus* | *sY412MC61* | 3.67 | 387 | TransportDB |
| Bacteria | *Geobacillus* | *sY41MC1* | 3.91 | 453 | TransportDB |
| Bacteria | *Geobacter* | *sulfurreducens* KN400 | 3.71 | 343 | TransportDB |
| Bacteria | *Geobacter* | *sulfurreducens* PCA | 3.81 | 342 | TransportDB |
| Bacteria | *Geobacillus* | *thermodenitrificans* NG802 | 3.61 | 437 | TransportDB |
| Bacteria | *Geobacillus* | *thermoglucosidasius* C56YS93 | 3.99 | 443 | TransportDB |
| Bacteria | *Geobacillus* | *thermoleovorans* CCB_US3_UF5 | 3.6 | 388 | TransportDB |
| Bacteria | *Granulicella* | *tundricola* MP5ACTX9 | 5.5 | 270 | TransportDB |
| Bacteria | *Geobacter* | *uraniireducens* Rf4 | 5.14 | 331 | TransportDB |
| Bacteria | *Gardnerella* | *vaginalis* 40905 | 1.62 | 180 | TransportDB |
| Bacteria | *Gardnerella* | *vaginalis* ATCC14019 | 1.67 | 219 | TransportDB |
| Bacteria | *Gardnerella* | *vaginalis* HMP9231 | 1.73 | 224 | TransportDB |
| Bacteria | *Gloeobacter* | *violaceus* PCC7421 | 4.66 | 284 | TransportDB |
| Bacteria | *Gluconacetobacter* | *xylinus* NBRC3288 | 3.51 | 273 | TransportDB |
| Bacteria | *Helicobacter* | *acinonychis* str Sheeba | 1.56 | 135 | TransportDB |
| Bacteria | *Herminiimonas* | *arsenicoxydans* | 3.42 | 339 | TransportDB |
| Bacteria | *Herpetosiphon* | *aurantiacus* DSM785 | 6.79 | 417 | TransportDB |
| Bacteria | *Hirschia* | *baltica* ATCC49814 | 3.54 | 238 | TransportDB |
| Bacteria | *Helicobacter* | *bizzozeronii* CIII1 | 1.81 | 124 | TransportDB |
| Bacteria | *Helicobacter* | *cetorum* MIT007128 | 1.96 | 120 | TransportDB |
| Bacteria | *Helicobacter* | *cetorum* MIT995656 | 1.85 | 129 | TransportDB |
| Bacteria | *Hahella* | *chejuensis* KCTC2396 | 7.22 | 590 | TransportDB |
| Bacteria | *Helicobacter* | *cinaedi* ATCCBAA847 | 2.24 | 167 | TransportDB |
| Bacteria | *Helicobacter* | *cinaedi* PAGU611 | 2.1 | 166 | TransportDB |
| Bacteria | *Hyphomicrobium* | *denitrificans* 1NES1 | 3.81 | 270 | TransportDB |
| Bacteria | *Hyphomicrobium* | *denitrificans* ATCC51888 | 3.64 | 247 | TransportDB |
| Bacteria | *Haemophilus* | *ducreyi* 35000HP | 1.7 | 168 | TransportDB |
| Bacteria | *Halomonas* | *elongata* DSM2581 | 4.06 | 582 | TransportDB |
| Bacteria | *Helicobacter* | *felis* ATCC49179 | 1.67 | 120 | TransportDB |
| Bacteria | *Halobacteroides* | *halobius* DSM5150 | 2.65 | 292 | TransportDB |
| Bacteria | *Halorhodospira* | *halophila* SL1 | 2.68 | 224 | TransportDB |
| Bacteria | *Halobacillus* | *halophilus* DSM2266 | 4.17 | 471 | TransportDB |
| Bacteria | *Halyomorpha* | *halys* carbekii | 1.15 | 74 | TransportDB |
| Bacteria | *Helicobacter* | *heilmannii* ASB14 | 1.8 | 127 | TransportDB |
| Bacteria | *Helicobacter* | *hepaticus* ATCC51449 | 1.8 | 150 | TransportDB |
| Bacteria | *Halanaerobium* | *hydrogeniformans* | 2.61 | 299 | TransportDB |
| Bacteria | *Haliscomenobacter* | *hydrossis* DSM1100 | 8.77 | 346 | TransportDB |
| Bacteria | *Haemophilus* | *influenzae* 10810 | 1.98 | 218 | TransportDB |
| Bacteria | *Haemophilus* | *influenzae* 86028NP | 1.91 | 201 | TransportDB |
| Bacteria | *Haemophilus* | *influenzae* F3031 | 1.99 | 195 | TransportDB |
| Bacteria | *Haemophilus* | *influenzae* F3047 | 2.01 | 194 | TransportDB |
| Bacteria | *Haemophilus* | *influenzae* KR494 | 1.86 | 217 | TransportDB |
| Bacteria | *Haemophilus* | *influenzae* PittEE | 1.81 | 191 | TransportDB |
| Bacteria | *Haemophilus* | *influenzae* PittGG | 1.89 | 191 | TransportDB |
| Bacteria | *Haemophilus* | *influenzae* R2846 | 1.82 | 209 | TransportDB |
| Bacteria | *Haemophilus* | *influenzae* R2866 | 1.93 | 211 | TransportDB |
| Bacteria | *Haemophilus* | *influenzae* RdKW20 | 1.83 | 216 | TransportDB |
| Bacteria | *Hippea* | *maritima* DSM10411 | 1.69 | 186 | TransportDB |
| Bacteria | *Heliobacterium* | *modesticaldum* Ice1 | 3.08 | 217 | TransportDB |
| Bacteria | *Helicobacter* | *mustelae* 12198 | 1.58 | 132 | TransportDB |
| Bacteria | *Halothiobacillus* | *neapolitanus* c2 | 2.58 | 214 | TransportDB |
| Bacteria | *Hyphomonas* | *neptunium* ATCC15444 | 3.71 | 252 | TransportDB |
| Bacteria | *Hyphomicrobium* | *nitrativorans* NL23 | 3.65 | 261 | TransportDB |
| Bacteria | *Haliangium* | *ochraceum* DSM14365 | 9.45 | 335 | TransportDB |
| Bacteria | *Halothermothrix* | *orenii* H168 | 2.58 | 325 | TransportDB |
| Bacteria | *Haemophilus* | *parainfluenzae* T3T1 | 2.09 | 282 | TransportDB |
| Bacteria | *Haemophilus* | *parasuis* SH0165 | 2.27 | 217 | TransportDB |
| Bacteria | *Haemophilus* | *parasuis* ZJ0906 | 2.32 | 220 | TransportDB |
| Bacteria | *Halanaerobium* | *praevalens* DSM2228 | 2.31 | 304 | TransportDB |
| Bacteria | *Helicobacter* | *pylori* 2017 | 1.55 | 140 | TransportDB |
| Bacteria | *Helicobacter* | *pylori* 2018 | 1.56 | 137 | TransportDB |
| Bacteria | *Helicobacter* | *pylori* 26695 | 1.67 | 144 | TransportDB |
| Bacteria | *Helicobacter* | *pylori* 35A | 1.57 | 139 | TransportDB |
| Bacteria | *Helicobacter* | *pylori* 51 | 1.59 | 142 | TransportDB |
| Bacteria | *Helicobacter* | *pylori* 52 | 1.57 | 142 | TransportDB |
| Bacteria | *Helicobacter* | *pylori* 83 | 1.62 | 144 | TransportDB |
| Bacteria | *Helicobacter* | *pylori* 908 | 1.55 | 140 | TransportDB |
| Bacteria | *Helicobacter* | *pylori* Aklavik117 | 1.64 | 134 | TransportDB |
| Bacteria | *Helicobacter* | *pylori* Aklavik86 | 1.51 | 127 | TransportDB |
| Bacteria | *Helicobacter* | *pylori* B38 | 1.58 | 127 | TransportDB |
| Bacteria | *Helicobacter* | *pylori* B8 | 1.68 | 143 | TransportDB |
| Bacteria | *Helicobacter* | *pylori* BM012A | 1.66 | 142 | TransportDB |
| Bacteria | *Helicobacter* | *pylori* BM012S | 1.66 | 142 | TransportDB |
| Bacteria | *Helicobacter* | *pylori* Cuz20 | 1.64 | 138 | TransportDB |
| Bacteria | *Helicobacter* | *pylori* ELS37 | 1.67 | 140 | TransportDB |
| Bacteria | *Helicobacter* | *pylori* F16 | 1.58 | 145 | TransportDB |
| Bacteria | *Helicobacter* | *pylori* F30 | 1.58 | 145 | TransportDB |
| Bacteria | *Helicobacter* | *pylori* F32 | 1.58 | 143 | TransportDB |
| Bacteria | *Helicobacter* | *pylori* F57 | 1.61 | 148 | TransportDB |
| Bacteria | *Helicobacter* | *pylori* G27 | 1.66 | 138 | TransportDB |
| Bacteria | *Helicobacter* | *pylori* Gambia9424 | 1.71 | 145 | TransportDB |
| Bacteria | *Helicobacter* | *pylori* HPAG1 | 1.61 | 146 | TransportDB |
| Bacteria | *Helicobacter* | *pylori* HUPB14 | 1.61 | 141 | TransportDB |
| Bacteria | *Helicobacter* | *pylori* India7 | 1.68 | 139 | TransportDB |
| Bacteria | *Helicobacter* | *pylori* J99 | 1.64 | 145 | TransportDB |
| Bacteria | *Helicobacter* | *pylori* Lithuania75 | 1.64 | 140 | TransportDB |
| Bacteria | *Helicobacter* | *pylori* OK113 | 1.62 | 144 | TransportDB |
| Bacteria | *Helicobacter* | *pylori* OK310 | 1.6 | 144 | TransportDB |
| Bacteria | *Helicobacter* | *pylori* P12 | 1.68 | 147 | TransportDB |
| Bacteria | *Helicobacter* | *pylori* PeCan18 | 1.66 | 144 | TransportDB |
| Bacteria | *Helicobacter* | *pylori* PeCan4 | 1.64 | 140 | TransportDB |
| Bacteria | *Helicobacter* | *pylori* Puno120 | 1.64 | 137 | TransportDB |
| Bacteria | *Helicobacter* | *pylori* Puno135 | 1.65 | 141 | TransportDB |
| Bacteria | *Helicobacter* | *pylori* Rif1 | 1.67 | 145 | TransportDB |
| Bacteria | *Helicobacter* | *pylori* Rif2 | 1.67 | 145 | TransportDB |
| Bacteria | *Helicobacter* | *pylori* Sat464 | 1.57 | 136 | TransportDB |
| Bacteria | *Helicobacter* | *pylori* Shi112 | 1.66 | 144 | TransportDB |
| Bacteria | *Helicobacter* | *pylori* Shi169 | 1.62 | 138 | TransportDB |
| Bacteria | *Helicobacter* | *pylori* Shi417 | 1.67 | 143 | TransportDB |
| Bacteria | *Helicobacter* | *pylori* Shi470 | 1.61 | 137 | TransportDB |
| Bacteria | *Helicobacter* | *pylori* SJM180 | 1.66 | 142 | TransportDB |
| Bacteria | *Helicobacter* | *pylori* SNT49 | 1.61 | 138 | TransportDB |
| Bacteria | *Helicobacter* | *pylori* South Africa 20 | 1.62 | 137 | TransportDB |
| Bacteria | *Helicobacter* | *pylori* South Africa 7 | 1.68 | 132 | TransportDB |
| Bacteria | *Helicobacter* | *pylori* UM032 | 1.6 | 146 | TransportDB |
| Bacteria | *Helicobacter* | *pylori* UM037 | 1.69 | 146 | TransportDB |
| Bacteria | *Helicobacter* | *pylori* UM066 | 1.66 | 147 | TransportDB |
| Bacteria | *Helicobacter* | *pylori* UM298 | 1.6 | 138 | TransportDB |
| Bacteria | *Helicobacter* | *pylori* UM299 | 1.6 | 146 | TransportDB |
| Bacteria | *Helicobacter* | *pylori* v225d | 1.6 | 140 | TransportDB |
| Bacteria | *Helicobacter* | *pylori* XZ274 | 1.66 | 110 | TransportDB |
| Bacteria | *Herbaspirillum* | *seropedicae* SmR1 | 5.51 | 689 | TransportDB |
| Bacteria | *Haemophilus* | *somnus* 129PT | 2.01 | 228 | TransportDB |
| Bacteria | *Haemophilus* | *somnus* 2336 | 2.26 | 254 | TransportDB |
| Bacteria | *Hydrogenobaculum* | *sHO* | 1.55 | 107 | TransportDB |
| Bacteria | *Hyphomicrobium* | *sMC1* | 4.76 | 439 | TransportDB |
| Bacteria | *Halothece* | *sPCC7418* | 4.18 | 320 | TransportDB |
| Bacteria | *Hydrogenobaculum* | *sSN* | 1.55 | 108 | TransportDB |
| Bacteria | *Hydrogenobaculum* | *sY04AAS1* | 1.56 | 111 | TransportDB |
| Bacteria | *Hydrogenobacter* | *thermophilus* TK6 | 1.74 | 129 | TransportDB |
| Bacteria | *Ignavibacterium* | *album* JCM16511 | 3.66 | 202 | TransportDB |
| Bacteria | *Intrasporangium* | *calvum* DSM43043 | 4.02 | 375 | TransportDB |
| Bacteria | *Ilumatobacter* | *coccineum* YM16304 | 4.83 | 366 | TransportDB |
| Bacteria | *Idiomarina* | *loihiensis* GSL199 | 2.84 | 237 | TransportDB |
| Bacteria | *Idiomarina* | *loihiensis* L2TR | 2.84 | 236 | TransportDB |
| Bacteria | *Isosphaera* | *pallida* ATCC43644 | 5.53 | 214 | TransportDB |
| Bacteria | *Ilyobacter* | *polytropus* DSM2926 | 3.13 | 337 | TransportDB |
| Bacteria | *Isoptericola* | *variabilis* 225 | 3.31 | 360 | TransportDB |
| Bacteria | *Jonesia* | *denitrificans* DSM20603 | 2.75 | 331 | TransportDB |
| Bacteria | *Jannaschia* | *sCCS1* | 4.4 | 555 | TransportDB |
| Bacteria | *Janthinobacterium* | *sMarseille* | 4.11 | 422 | TransportDB |
| Bacteria | *Kribbella* | *flavida* DSM17836 | 7.58 | 773 | TransportDB |
| Bacteria | *Kangiella* | *koreensis* DSM16069 | 2.85 | 190 | TransportDB |
| Bacteria | *Kosmotoga* | *olearia* TBF1951 | 2.3 | 293 | TransportDB |
| Bacteria | *Klebsiella* | *oxytoca* E718 | 6.56 | 997 | TransportDB |
| Bacteria | *Klebsiella* | *oxytoca* KCTC1686 | 5.97 | 977 | TransportDB |
| Bacteria | *Klebsiella* | *pneumoniae* | 5.38 | 869 | TransportDB |
| Bacteria | *Klebsiella* | *pneumoniae* 342 | 5.92 | 933 | TransportDB |
| Bacteria | *Klebsiella* | *pneumoniae* CG43 | 5.17 | 844 | TransportDB |
| Bacteria | *Klebsiella* | *pneumoniae* JM45 | 5.6 | 835 | TransportDB |
| Bacteria | *Klebsiella* | *pneumoniae* KCTC2242 | 5.46 | 893 | TransportDB |
| Bacteria | *Klebsiella* | *pneumoniae* subsp pneumoniae1084 | 5.39 | 870 | TransportDB |
| Bacteria | *Klebsiella* | *pneumoniae* subsp pneumoniae HS11286 | 5.68 | 881 | TransportDB |
| Bacteria | *Klebsiella* | *pneumoniae* subsp pneumoniae MGH78578 | 5.69 | 884 | TransportDB |
| Bacteria | *Klebsiella* | *pneumoniae* subsp pneumoniae NTUHK2044 | 5.47 | 893 | TransportDB |
| Bacteria | *Kineococcus* | *radiotolerans* SRS30216 | 4.96 | 482 | TransportDB |
| Bacteria | *Kocuria* | *rhizophila* DC2201 | 2.7 | 262 | TransportDB |
| Bacteria | *Kytococcus* | *sedentarius* DSM20547 | 2.79 | 299 | TransportDB |
| Bacteria | *Kitasatospora* | *setae* KM6054 | 8.78 | 682 | TransportDB |
| Bacteria | *Krokinobacter* | *s4H375* | 3.39 | 201 | TransportDB |
| Bacteria | *Kyrpidia* | *tusciae* DSM2912 | 3.38 | 299 | TransportDB |
| Bacteria | *Klebsiella* | *variicola* At22 | 5.46 | 917 | TransportDB |
| Bacteria | *Korebacter* | *versatilis* Ellin 345 | 5.65 | 307 | TransportDB |
| Bacteria | *Ketogulonicigenium* | *vulgare* WSH001 | 3.28 | 560 | TransportDB |
| Bacteria | *Ketogulonicigenium* | *vulgare* Y25 | 3.29 | 591 | TransportDB |
| Bacteria | *Lactobacillus* | *acidophilus* 30SC | 2.1 | 266 | TransportDB |
| Bacteria | *Lactobacillus* | *acidophilus* La14 | 1.99 | 281 | TransportDB |
| Bacteria | *Lactobacillus* | *acidophilus* NCFM | 1.99 | 281 | TransportDB |
| Bacteria | *Lactobacillus* | *amylovorus* GRL1112 | 2.13 | 257 | TransportDB |
| Bacteria | *Lactobacillus* | *amylovorus* GRL1118 | 1.98 | 253 | TransportDB |
| Bacteria | *Listonella* | *anguillarum* M3 | 4.12 | 442 | TransportDB |
| Bacteria | *Leptospira* | *biflexa* serovar Patocstrain Patoc1 Ames | 3.96 | 230 | TransportDB |
| Bacteria | *Leptospira* | *biflexa* serovar Patocstrain Patoc1 Paris | 3.95 | 231 | TransportDB |
| Bacteria | *Leptospira* | *borgpetersenii* serovar Hardjobovis str JB197 | 3.88 | 166 | TransportDB |
| Bacteria | *Leptospira* | *borgpetersenii* serovar Hardjobovis str L550 | 3.93 | 172 | TransportDB |
| Bacteria | *Lactobacillus* | *brevis* ATCC367 | 2.34 | 295 | TransportDB |
| Bacteria | *Lactobacillus* | *brevis* KB290 | 2.59 | 332 | TransportDB |
| Bacteria | *Leptotrichia* | *buccalis* C1013b | 2.47 | 236 | TransportDB |
| Bacteria | *Lactobacillus* | *buchneri* CD034 | 2.56 | 324 | TransportDB |
| Bacteria | *Lactobacillus* | *buchneri* NRRLB30929 | 2.59 | 310 | TransportDB |
| Bacteria | *Leadbetterella* | *byssophila* DSM17132 | 4.06 | 206 | TransportDB |
| Bacteria | *Leuconostoc* | *carnosum* JB16 | 1.77 | 257 | TransportDB |
| Bacteria | *Lactobacillus* | *casei* ATCC334 | 2.92 | 387 | TransportDB |
| Bacteria | *Lactobacillus* | *casei* BDII | 3.13 | 475 | TransportDB |
| Bacteria | *Lactobacillus* | *casei* BL23 | 3.08 | 479 | TransportDB |
| Bacteria | *Lactobacillus* | *casei* LC2W | 3.08 | 475 | TransportDB |
| Bacteria | *Lactobacillus* | *casei* LOCK919 | 3.14 | 474 | TransportDB |
| Bacteria | *Lactobacillus* | *casei* str Zhang | 2.9 | 437 | TransportDB |
| Bacteria | *Lactobacillus* | *casei* W56 | 3.13 | 471 | TransportDB |
| Bacteria | *Leptothrix* | *cholodnii* SP6 | 4.91 | 502 | TransportDB |
| Bacteria | *Leuconostoc* | *citreum* KM20 | 1.9 | 260 | TransportDB |
| Bacteria | *Lactobacillus* | *crispatus* ST1 | 2.04 | 294 | TransportDB |
| Bacteria | *Lactobacillus* | *delbrueckii* subsp bulgaricus 2038 | 1.87 | 261 | TransportDB |
| Bacteria | *Lactobacillus* | *delbrueckii* subsp bulgaricus ATCC11842 | 1.87 | 202 | TransportDB |
| Bacteria | *Lactobacillus* | *delbrueckii* subsp bulgaricus ATCCBAA365 | 1.86 | 198 | TransportDB |
| Bacteria | *Lactobacillus* | *delbrueckii* subsp bulgaricus ND02 | 2.13 | 245 | TransportDB |
| Bacteria | *Lactobacillus* | *fermentum* CECT5716 | 2.1 | 172 | TransportDB |
| Bacteria | *Lactobacillus* | *fermentum* F6 | 2.06 | 231 | TransportDB |
| Bacteria | *Lactobacillus* | *fermentum* IFO3956 | 2.1 | 222 | TransportDB |
| Bacteria | *Leptospirillum* | *ferriphilum* ML04 | 2.41 | 159 | TransportDB |
| Bacteria | *Leptospirillum* | *ferrooxidans* C23 | 2.56 | 159 | TransportDB |
| Bacteria | *Lactococcus* | *garvieae* ATCC49156 | 1.95 | 248 | TransportDB |
| Bacteria | *Lactococcus* | *garvieae* Lg2 | 1.96 | 245 | TransportDB |
| Bacteria | *Leuconostoc* | *gasicomitatum* LMG18811 | 1.95 | 287 | TransportDB |
| Bacteria | *Lactobacillus* | *gasseri* ATCC33323 | 1.89 | 254 | TransportDB |
| Bacteria | *Leuconostoc* | *gelidum* JB7 | 1.89 | 274 | TransportDB |
| Bacteria | *Lactobacillus* | *helveticus* CNRZ32 | 2.23 | 227 | TransportDB |
| Bacteria | *Lactobacillus* | *helveticus* DPC4571 | 2.08 | 214 | TransportDB |
| Bacteria | *Lactobacillus* | *helveticus* H10 | 2.17 | 256 | TransportDB |
| Bacteria | *Lactobacillus* | *helveticus* R0052 | 2.13 | 245 | TransportDB |
| Bacteria | *Laribacter* | *hongkongensis* HLHK9 | 3.17 | 331 | TransportDB |
| Bacteria | *Listeria* | *innocua* Clip11262 | 3.09 | 439 | TransportDB |
| Bacteria | *Leptospira* | *interrogans* serovar Copenhageni str Fiocruz L1130 | 4.63 | 189 | TransportDB |
| Bacteria | *Leptospira* | *interrogans* serovar Lai str 56601 | 4.7 | 199 | TransportDB |
| Bacteria | *Leptospira* | *interrogans* serovar Lai str IPAV | 4.71 | 198 | TransportDB |
| Bacteria | *Lawsonia* | *intracellularis* N343 | 1.72 | 154 | TransportDB |
| Bacteria | *Lawsonia* | *intracellularis* PHEMN100 | 1.72 | 154 | TransportDB |
| Bacteria | *Listeria* | *ivanovii* subsp ivanovii PAM55 | 2.93 | 423 | TransportDB |
| Bacteria | *Lactobacillus* | *johnsonii* DPC6026 | 1.97 | 287 | TransportDB |
| Bacteria | *Lactobacillus* | *johnsonii* FI9785 | 1.79 | 272 | TransportDB |
| Bacteria | *Lactobacillus* | *johnsonii* N62 | 1.89 | 273 | TransportDB |
| Bacteria | *Lactobacillus* | *johnsonii* NCC533 | 1.99 | 306 | TransportDB |
| Bacteria | *Lactobacillus* | *kefiranofaciens* ZW3 | 2.35 | 243 | TransportDB |
| Bacteria | *Leuconostoc* | *kimchii* IMSNU11154 | 2.1 | 300 | TransportDB |
| Bacteria | *Lactococcus* | *lactis* subsp cremorisA76 | 2.58 | 317 | TransportDB |
| Bacteria | *Lactococcus* | *lactis* subsp cremoris KW2 | 2.43 | 308 | TransportDB |
| Bacteria | *Lactococcus* | *lactis* subsp cremoris MG1363 | 2.53 | 285 | TransportDB |
| Bacteria | *Lactococcus* | *lactis* subsp cremoris NZ9000 | 2.53 | 286 | TransportDB |
| Bacteria | *Lactococcus* | *lactis* subsp cremoris SK11 | 2.6 | 272 | TransportDB |
| Bacteria | *Lactococcus* | *lactis* subsp cremoris UC5099 | 2.46 | 264 | TransportDB |
| Bacteria | *Lactococcus* | *lactis* subsp lactis CV56 | 2.52 | 284 | TransportDB |
| Bacteria | *Lactococcus* | *lactis* subsp lactis Il1403 | 2.37 | 268 | TransportDB |
| Bacteria | *Lactococcus* | *lactis* subsp lactis IO1 | 2.42 | 298 | TransportDB |
| Bacteria | *Lactococcus* | *lactis* subsp lactis KF147 | 2.64 | 330 | TransportDB |
| Bacteria | *Lactococcus* | *lactis* subsp lactis KLDS40325 | 2.59 | 283 | TransportDB |
| Bacteria | *Legionella* | *longbeachae* NSW150 | 4.15 | 313 | TransportDB |
| Bacteria | *Leuconostoc* | *mesenteroides* subsp mesenteroides ATCC8293 | 2.08 | 298 | TransportDB |
| Bacteria | *Leuconostoc* | *mesenteroides* subsp mesenteroides J18 | 2.02 | 286 | TransportDB |
| Bacteria | *Leisingera* | *methylohalidivorans* DSM14336 | 4.65 | 471 | TransportDB |
| Bacteria | *Listeria* | *monocytogenes* | 2.78 | 485 | TransportDB |
| Bacteria | *Listeria* | *monocytogenes* 07PF0776 | 2.9 | 429 | TransportDB |
| Bacteria | *Listeria* | *monocytogenes* 085578 | 3.11 | 447 | TransportDB |
| Bacteria | *Listeria* | *monocytogenes* 085923 | 3.0 | 439 | TransportDB |
| Bacteria | *Listeria* | *monocytogenes* 10403S | 2.9 | 436 | TransportDB |
| Bacteria | *Listeria* | *monocytogenes* ATCC19117 | 2.95 | 440 | TransportDB |
| Bacteria | *Listeria* | *monocytogenes* EGD | 2.91 | 436 | TransportDB |
| Bacteria | *Listeria* | *monocytogenes* EGDe | 2.94 | 434 | TransportDB |
| Bacteria | *Listeria* | *monocytogenes* Finland1998 | 2.87 | 436 | TransportDB |
| Bacteria | *Listeria* | *monocytogenes* FSLR2561 | 2.97 | 437 | TransportDB |
| Bacteria | *Listeria* | *monocytogenes* HCC23 | 2.98 | 421 | TransportDB |
| Bacteria | *Listeria* | *monocytogenes* J0161 | 3.0 | 441 | TransportDB |
| Bacteria | *Listeria* | *monocytogenes* J1220 | 3.03 | 433 | TransportDB |
| Bacteria | *Listeria* | *monocytogenes* J1816 | 2.95 | 279 | TransportDB |
| Bacteria | *Listeria* | *monocytogenes* L312 | 2.91 | 440 | TransportDB |
| Bacteria | *Listeria* | *monocytogenes* L99 | 2.98 | 421 | TransportDB |
| Bacteria | *Listeria* | *monocytogenes* M7 | 2.98 | 422 | TransportDB |
| Bacteria | *Listeria* | *monocytogenes* N531 | 2.78 | 487 | TransportDB |
| Bacteria | *Listeria* | *monocytogenes* serotype 4b str CLIP80459 | 2.91 | 439 | TransportDB |
| Bacteria | *Listeria* | *monocytogenes* serotype 4b str F2365 | 2.91 | 422 | TransportDB |
| Bacteria | *Listeria* | *monocytogenes* serotype 4b str LL195 | 2.9 | 431 | TransportDB |
| Bacteria | *Listeria* | *monocytogenes* serotype 7 str SLCC2482 | 2.99 | 439 | TransportDB |
| Bacteria | *Listeria* | *monocytogenes* SLCC2372 | 3.02 | 440 | TransportDB |
| Bacteria | *Listeria* | *monocytogenes* SLCC2376 | 2.84 | 431 | TransportDB |
| Bacteria | *Listeria* | *monocytogenes* SLCC2378 | 2.94 | 443 | TransportDB |
| Bacteria | *Listeria* | *monocytogenes* SLCC2479 | 2.97 | 438 | TransportDB |
| Bacteria | *Listeria* | *monocytogenes* SLCC2540 | 2.98 | 438 | TransportDB |
| Bacteria | *Listeria* | *monocytogenes* SLCC2755 | 3.02 | 441 | TransportDB |
| Bacteria | *Listeria* | *monocytogenes* SLCC5850 | 2.91 | 436 | TransportDB |
| Bacteria | *Listeria* | *monocytogenes* SLCC7179 | 2.88 | 434 | TransportDB |
| Bacteria | *Lactobacillus* | *paracasei* subsp paracasei8700 | 3.03 | 451 | TransportDB |
| Bacteria | *Lactobacillus* | *plantarum* 16 | 3.36 | 427 | TransportDB |
| Bacteria | *Lactobacillus* | *plantarum* JDM1 | 3.2 | 446 | TransportDB |
| Bacteria | *Lactobacillus* | *plantarum* subsp plantarum P8 | 3.23 | 418 | TransportDB |
| Bacteria | *Lactobacillus* | *plantarum* subsp plantarum STIII | 3.31 | 441 | TransportDB |
| Bacteria | *Lactobacillus* | *plantarum* WCFS1 | 3.35 | 453 | TransportDB |
| Bacteria | *Lactobacillus* | *plantarum* ZJ316 | 3.2 | 438 | TransportDB |
| Bacteria | *Legionella* | *pneumophila* 230099 Alcoy | 3.52 | 290 | TransportDB |
| Bacteria | *Legionella* | *pneumophila* str Corby | 3.58 | 294 | TransportDB |
| Bacteria | *Legionella* | *pneumophila* str Lens | 3.41 | 271 | TransportDB |
| Bacteria | *Legionella* | *pneumophila* str Paris | 3.64 | 290 | TransportDB |
| Bacteria | *Legionella* | *pneumophila* subsp pneumophila | 3.49 | 292 | TransportDB |
| Bacteria | *Legionella* | *pneumophila* subsp pneumophila ATCC43290 | 3.36 | 275 | TransportDB |
| Bacteria | *Legionella* | *pneumophila* subsp pneumophila LPE509 | 3.51 | 304 | TransportDB |
| Bacteria | *Legionella* | *pneumophila* subsp pneumophila str Lorraine | 3.62 | 293 | TransportDB |
| Bacteria | *Legionella* | *pneumophila* subsp pneumophila str Philadelphia1 | 3.4 | 288 | TransportDB |
| Bacteria | *Legionella* | *pneumophila* subsp pneumophila str Thunder Bay | 3.46 | 295 | TransportDB |
| Bacteria | *Lactobacillus* | *reuteri* DSM20016 | 2.0 | 223 | TransportDB |
| Bacteria | *Lactobacillus* | *reuteri* I5007 | 2.09 | 223 | TransportDB |
| Bacteria | *Lactobacillus* | *reuteri* JCM1112 | 2.04 | 223 | TransportDB |
| Bacteria | *Lactobacillus* | *reuteri* SD2112 | 2.32 | 233 | TransportDB |
| Bacteria | *Lactobacillus* | *reuteri* TD1 | 2.15 | 217 | TransportDB |
| Bacteria | *Lactobacillus* | *rhamnosus* ATCC8530 | 2.96 | 443 | TransportDB |
| Bacteria | *Lactobacillus* | *rhamnosus* GG | 3.01 | 439 | TransportDB |
| Bacteria | *Lactobacillus* | *rhamnosus* Lc705 | 3.03 | 474 | TransportDB |
| Bacteria | *Lactobacillus* | *rhamnosus* LOCK900 | 2.88 | 439 | TransportDB |
| Bacteria | *Lactobacillus* | *rhamnosus* LOCK908 | 2.99 | 448 | TransportDB |
| Bacteria | *Lactobacillus* | *ruminis* ATCC27782 | 2.07 | 238 | TransportDB |
| Bacteria | *Lactobacillus* | *sakei* subsp sakei 23K | 1.88 | 265 | TransportDB |
| Bacteria | *Lactobacillus* | *salivarius* CECT5713 | 2.14 | 238 | TransportDB |
| Bacteria | *Lactobacillus* | *salivarius* UCC118 | 2.13 | 241 | TransportDB |
| Bacteria | *Lactobacillus* | *sanfranciscensis* TMW11304 | 1.38 | 158 | TransportDB |
| Bacteria | *Listeria* | *seeligeri* serovar 12b str SLCC3954 | 2.8 | 388 | TransportDB |
| Bacteria | *Lacinutrix* | *s5H374* | 3.3 | 189 | TransportDB |
| Bacteria | *Leuconostoc* | *sC2* | 1.88 | 280 | TransportDB |
| Bacteria | *Leptolyngbya* | *sPCC7376* | 5.13 | 298 | TransportDB |
| Bacteria | *Lysinibacillus* | *sphaericus* C341 | 4.82 | 566 | TransportDB |
| Bacteria | *Listeria* | *welshimeri* serovar 6b str SLCC5334 | 2.81 | 413 | TransportDB |
| Bacteria | *Leifsonia* | *xyli* subsp cynodontis DSM46306 | 2.69 | 223 | TransportDB |
| Bacteria | *Leifsonia* | *xyli* subsp xyli str CTCB07 | 2.58 | 199 | TransportDB |
| Bacteria | *Mycobacterium* | *abscessus* | 5.09 | 388 | TransportDB |
| Bacteria | *Mycobacterium* | *abscessus* ATCC19977 | 5.09 | 388 | TransportDB |
| Bacteria | *Mycobacterium* | *abscessus* subsp bolletii50594 | 5.27 | 375 | TransportDB |
| Bacteria | *Marinobacter* | *adhaerens* HP15 | 4.65 | 484 | TransportDB |
| Bacteria | *Microcystis* | *aeruginosa* NIES843 | 5.84 | 291 | TransportDB |
| Bacteria | *Micavibrio* | *aeruginosavorus* ARL13 | 2.48 | 143 | TransportDB |
| Bacteria | *Micavibrio* | *aeruginosavorus* EPB | 2.46 | 137 | TransportDB |
| Bacteria | *Mycobacterium* | *africanum* GM041182 | 4.39 | 255 | TransportDB |
| Bacteria | *Mycoplasma* | *agalactiae* | 1.01 | 86 | TransportDB |
| Bacteria | *Mycoplasma* | *agalactiae* PG2 | 0.88 | 81 | TransportDB |
| Bacteria | *Methylomicrobium* | *alcaliphilum* 20Z | 4.67 | 293 | TransportDB |
| Bacteria | *Marinobacter* | *aquaeolei* VT8 | 4.78 | 437 | TransportDB |
| Bacteria | *Mycoplasma* | *arthritidis* 158L31 | 0.82 | 58 | TransportDB |
| Bacteria | *Micromonospora* | *aurantiaca* ATCC27029 | 7.03 | 575 | TransportDB |
| Bacteria | *Mesorhizobium* | *australicum* WSM2073 | 6.2 | 877 | TransportDB |
| Bacteria | *Mahella* | *australiensis* 501BON | 3.14 | 396 | TransportDB |
| Bacteria | *Mycobacterium* | *avium* 104 | 5.48 | 297 | TransportDB |
| Bacteria | *Mycobacterium* | *avium* subsp paratuberculosis K10 | 4.83 | 316 | TransportDB |
| Bacteria | *Mycobacterium* | *avium* subsp paratuberculosis MAP4 | 4.83 | 305 | TransportDB |
| Bacteria | *Mycobacterium* | *bovis* AF212297 | 4.35 | 264 | TransportDB |
| Bacteria | *Mycobacterium* | *bovis* BCG str Korea1168P | 4.38 | 263 | TransportDB |
| Bacteria | *Mycobacterium* | *bovis* BCG str Mexico | 4.35 | 260 | TransportDB |
| Bacteria | *Mycobacterium* | *bovis* BCG str Pasteur1173P2 | 4.37 | 261 | TransportDB |
| Bacteria | *Mycobacterium* | *bovis* BCG str Tokyo172 | 4.37 | 262 | TransportDB |
| Bacteria | *Mycoplasma* | *bovis* HB0801 | 0.99 | 72 | TransportDB |
| Bacteria | *Mycoplasma* | *bovis* Hubei1 | 0.95 | 76 | TransportDB |
| Bacteria | *Mycoplasma* | *bovis* PG45 | 1.0 | 77 | TransportDB |
| Bacteria | *Mycobacterium* | *canettii* CIPT140010059 | 4.48 | 265 | TransportDB |
| Bacteria | *Mycobacterium* | *canettii* CIPT140060008 | 4.43 | 266 | TransportDB |
| Bacteria | *Mycobacterium* | *canettii* CIPT140070008 | 4.42 | 266 | TransportDB |
| Bacteria | *Mycobacterium* | *canettii* CIPT140070010 | 4.53 | 270 | TransportDB |
| Bacteria | *Mycobacterium* | *canettii* CIPT140070017 | 4.52 | 273 | TransportDB |
| Bacteria | *Mycoplasma* | *capricolum* subsp capricolum ATCC27343 | 1.01 | 91 | TransportDB |
| Bacteria | *Methylococcus* | *capsulatus* str Bath | 3.3 | 243 | TransportDB |
| Bacteria | *Macrococcus* | *caseolyticus* JCSC5402 | 2.22 | 257 | TransportDB |
| Bacteria | *Moraxella* | *catarrhalis* RH4 | 1.86 | 153 | TransportDB |
| Bacteria | *Mycobacterium* | *chubuense* NBB4 | 6.34 | 491 | TransportDB |
| Bacteria | *Mesorhizobium* | *ciceri* biovar biserrulae WSM1271 | 6.69 | 973 | TransportDB |
| Bacteria | *Mycoplasma* | *conjunctivae* HRC581 | 0.85 | 77 | TransportDB |
| Bacteria | *Mycoplasma* | *crocodyli* MP145 | 0.93 | 88 | TransportDB |
| Bacteria | *Mobiluncus* | *curtisii* ATCC43063 | 2.15 | 212 | TransportDB |
| Bacteria | *Mycoplasma* | *cynos* C142 | 1.0 | 78 | TransportDB |
| Bacteria | *Megasphaera* | *elsdenii* DSM20460 | 2.47 | 235 | TransportDB |
| Bacteria | *Methylobacterium* | *extorquens* AM1 | 6.88 | 469 | TransportDB |
| Bacteria | *Methylobacterium* | *extorquens* DM4 | 6.12 | 495 | TransportDB |
| Bacteria | *Methylobacterium* | *extorquens* PA1 | 5.47 | 430 | TransportDB |
| Bacteria | *Mycoplasma* | *fermentans* JER | 0.98 | 90 | TransportDB |
| Bacteria | *Mycoplasma* | *fermentans* M64 | 1.12 | 105 | TransportDB |
| Bacteria | *Mycoplasma* | *fermentans* PG18 | 1.0 | 99 | TransportDB |
| Bacteria | *Methylobacillus* | *flagellatus* KT | 2.97 | 273 | TransportDB |
| Bacteria | *Mycobacterium* | *flavenscens* (gilvum) PYR-GCK | 5.62 | 464 | TransportDB |
| Bacteria | *Mesoplasma* | *florum* L1 | 0.79 | 78 | TransportDB |
| Bacteria | *Mesoplasma* | *florum* W37 | 0.83 | 83 | TransportDB |
| Bacteria | *Myxococcus* | *fulvus* HW1 | 9.0 | 404 | TransportDB |
| Bacteria | *Mycoplasma* | *gallisepticum* CA06_200605252P | 0.98 | 83 | TransportDB |
| Bacteria | *Mycoplasma* | *gallisepticum* NC06_200608052P | 0.94 | 83 | TransportDB |
| Bacteria | *Mycoplasma* | *gallisepticum* NC08_200803143P | 0.93 | 83 | TransportDB |
| Bacteria | *Mycoplasma* | *gallisepticum* NC95_1329522P | 0.95 | 84 | TransportDB |
| Bacteria | *Mycoplasma* | *gallisepticum* NC96_159642P | 0.99 | 87 | TransportDB |
| Bacteria | *Mycoplasma* | *gallisepticum* NY01_200104751P | 0.97 | 84 | TransportDB |
| Bacteria | *Mycoplasma* | *gallisepticum* R | 1.0 | 93 | TransportDB |
| Bacteria | *Mycoplasma* | *gallisepticum* S6 | 0.99 | 76 | TransportDB |
| Bacteria | *Mycoplasma* | *gallisepticum* str F | 0.98 | 88 | TransportDB |
| Bacteria | *Mycoplasma* | *gallisepticum* str Rhigh | 1.01 | 82 | TransportDB |
| Bacteria | *Mycoplasma* | *gallisepticum* str Rlow | 1.01 | 82 | TransportDB |
| Bacteria | *Mycoplasma* | *gallisepticum* VA94_799417P | 0.96 | 85 | TransportDB |
| Bacteria | *Mycoplasma* | *gallisepticum* WI01_2001043132P | 0.94 | 82 | TransportDB |
| Bacteria | *Mycoplasma* | *genitalium* G37 | 0.58 | 53 | TransportDB |
| Bacteria | *Mycoplasma* | *genitalium* M2288 | 0.58 | 52 | TransportDB |
| Bacteria | *Mycoplasma* | *genitalium* M2321 | 0.58 | 50 | TransportDB |
| Bacteria | *Mycoplasma* | *genitalium* M6282 | 0.58 | 46 | TransportDB |
| Bacteria | *Mycoplasma* | *genitalium* M6320 | 0.58 | 51 | TransportDB |
| Bacteria | *Mycobacterium* | *gilvum* PYRGCK | 5.98 | 447 | TransportDB |
| Bacteria | *Mycobacterium* | *gilvum* Spyr1 | 5.78 | 422 | TransportDB |
| Bacteria | *Methylovorus* | *glucosetrophus* SIP34 | 3.08 | 233 | TransportDB |
| Bacteria | *Magnetospirillum* | *gryphiswaldense* MSR1v2 | 4.37 | 366 | TransportDB |
| Bacteria | *Mycoplasma* | *haemocanis* str Illinois | 0.92 | 30 | TransportDB |
| Bacteria | *Mycoplasma* | *haemofelis* Ohio2 | 1.16 | 29 | TransportDB |
| Bacteria | *Mycoplasma* | *haemofelis* str Langford1 | 1.15 | 31 | TransportDB |
| Bacteria | *Mannheimia* | *haemolytica* D153 | 2.68 | 297 | TransportDB |
| Bacteria | *Mannheimia* | *haemolytica* D171 | 2.5 | 297 | TransportDB |
| Bacteria | *Mannheimia* | *haemolytica* D174 | 2.7 | 297 | TransportDB |
| Bacteria | *Mannheimia* | *haemolytica* M42548 | 2.73 | 329 | TransportDB |
| Bacteria | *Mannheimia* | *haemolytica* USDAARSUSMARC183 | 2.66 | 328 | TransportDB |
| Bacteria | *Mannheimia* | *haemolytica* USDAARSUSMARC185 | 2.54 | 331 | TransportDB |
| Bacteria | *Mannheimia* | *haemolytica* USMARC_2286 | 2.66 | 320 | TransportDB |
| Bacteria | *Mycoplasma* | *hominis* ATCC23114 | 0.67 | 51 | TransportDB |
| Bacteria | *Marinobacter* | *hydrocarbonoclasticus* (aquaeolei) VT8 | 4.33 | 433 | TransportDB |
| Bacteria | *Marinobacter* | *hydrocarbonoclasticus* ATCC49840 | 3.99 | 411 | TransportDB |
| Bacteria | *Marinithermus* | *hydrothermalis* DSM14884 | 2.27 | 257 | TransportDB |
| Bacteria | *Mycoplasma* | *hyopneumoniae* 168 | 0.93 | 92 | TransportDB |
| Bacteria | *Mycoplasma* | *hyopneumoniae* 168L | 0.92 | 92 | TransportDB |
| Bacteria | *Mycoplasma* | *hyopneumoniae* 232 | 0.89 | 97 | TransportDB |
| Bacteria | *Mycoplasma* | *hyopneumoniae* 7422 | 0.9 | 92 | TransportDB |
| Bacteria | *Mycoplasma* | *hyopneumoniae* 7448 | 0.92 | 92 | TransportDB |
| Bacteria | *Mycoplasma* | *hyopneumoniae* J | 0.9 | 90 | TransportDB |
| Bacteria | *Mycoplasma* | *hyorhinis* DBS1050 | 0.84 | 80 | TransportDB |
| Bacteria | *Mycoplasma* | *hyorhinis* GDL1 | 0.84 | 76 | TransportDB |
| Bacteria | *Mycoplasma* | *hyorhinis* HUB1 | 0.84 | 71 | TransportDB |
| Bacteria | *Mycoplasma* | *hyorhinis* MCLD | 0.83 | 77 | TransportDB |
| Bacteria | *Mycoplasma* | *hyorhinis* SK76 | 0.84 | 84 | TransportDB |
| Bacteria | *Megamonas* | *hypermegale* ART121 | 2.21 | 290 | TransportDB |
| Bacteria | *Mycobacterium* | *indicus* praniiMTCC9506 | 5.59 | 322 | TransportDB |
| Bacteria | *Methylacidiphilum* | *infernorum* V4 | 2.29 | 120 | TransportDB |
| Bacteria | *Mycobacterium* | *intracellulare* ATCC13950 | 5.4 | 308 | TransportDB |
| Bacteria | *Mycobacterium* | *intracellulare* MOTT02 | 5.41 | 304 | TransportDB |
| Bacteria | *Mycobacterium* | *intracellulare* MOTT64 | 5.5 | 317 | TransportDB |
| Bacteria | *Mycobacterium* | *kansasii* ATCC12478 | 6.58 | 347 | TransportDB |
| Bacteria | *Mycoplasma* | *leachii* 990146 | 1.02 | 104 | TransportDB |
| Bacteria | *Mycoplasma* | *leachii* PG50 | 1.01 | 104 | TransportDB |
| Bacteria | *Mycobacterium* | *leprae* Br4923 | 3.27 | 116 | TransportDB |
| Bacteria | *Mycobacterium* | *leprae* TN | 3.27 | 114 | TransportDB |
| Bacteria | *Mycobacterium* | *liflandii* 128FXT | 6.4 | 314 | TransportDB |
| Bacteria | *Mesorhizobium* | *loti* MAFF303099 | 7.6 | 968 | TransportDB |
| Bacteria | *Micrococcus* | *luteus* NCTC2665 | 2.5 | 225 | TransportDB |
| Bacteria | *Magnetospirillum* | *magneticum* AMB1 | 4.97 | 374 | TransportDB |
| Bacteria | *Mycobacterium* | *marinum* M | 6.66 | 385 | TransportDB |
| Bacteria | *Modestobacter* | *marinus* | 5.58 | 528 | TransportDB |
| Bacteria | *Magnetococcus* | *marinus* MC1 | 4.72 | 237 | TransportDB |
| Bacteria | *Maricaulis* | *maris* MCS10 | 3.37 | 231 | TransportDB |
| Bacteria | *Mycobacterium* | *massiliense* str GO06 | 5.07 | 293 | TransportDB |
| Bacteria | *Marinomonas* | *mediterranea* MMB1 | 4.68 | 535 | TransportDB |
| Bacteria | *Methylomonas* | *methanica* MC09 | 5.05 | 298 | TransportDB |
| Bacteria | *Mycoplasma* | *mobile* 163K | 0.78 | 74 | TransportDB |
| Bacteria | *Methylotenera* | *mobilis* JLW8 | 2.55 | 193 | TransportDB |
| Bacteria | *Morganella* | *morganii* subsp morganii KT | 3.8 | 492 | TransportDB |
| Bacteria | *Mycoplasma* | *mycoides* subsp capriLC str 95010 | 1.16 | 104 | TransportDB |
| Bacteria | *Mycoplasma* | *mycoides* subsp mycoides SC str Gladysdale | 1.19 | 112 | TransportDB |
| Bacteria | *Mycoplasma* | *mycoides* subsp mycoides SC str PG1 | 1.21 | 104 | TransportDB |
| Bacteria | *Mycobacterium* | *neoaurum* VKMAc1815D | 5.44 | 508 | TransportDB |
| Bacteria | *Methylobacterium* | *nodulans* ORS2060 | 8.84 | 762 | TransportDB |
| Bacteria | *Mesorhizobium* | *opportunistum* WSM2075 | 6.88 | 981 | TransportDB |
| Bacteria | *Mycoplasma* | *ovis* str Michigan | 0.7 | 33 | TransportDB |
| Bacteria | *Mycoplasma* | *parvum* str Indiana | 0.56 | 33 | TransportDB |
| Bacteria | *Mycoplasma* | *penetrans* HF2 | 1.36 | 99 | TransportDB |
| Bacteria | *Methylibium* | *petroleiphilum* PM1 | 4.64 | 387 | TransportDB |
| Bacteria | *Microlunatus* | *phosphovorus* NM1 | 5.68 | 515 | TransportDB |
| Bacteria | *Marinitoga* | *piezophila* KA3 | 2.24 | 278 | TransportDB |
| Bacteria | *Melissococcus* | *plutonius* ATCC35311 | 2.07 | 295 | TransportDB |
| Bacteria | *Melissococcus* | *plutonius* DAT561 | 2.05 | 267 | TransportDB |
| Bacteria | *Mycoplasma* | *pneumoniae* 309 | 0.82 | 69 | TransportDB |
| Bacteria | *Mycoplasma* | *pneumoniae* FH | 0.81 | 60 | TransportDB |
| Bacteria | *Mycoplasma* | *pneumoniae* M129 | 0.82 | 70 | TransportDB |
| Bacteria | *Mycoplasma* | *pneumoniae* M129B7 | 0.82 | 69 | TransportDB |
| Bacteria | *Methylobacterium* | *populi* BJ001 | 5.85 | 474 | TransportDB |
| Bacteria | *Marinomonas* | *posidonica* IVIAPo181 | 3.9 | 533 | TransportDB |
| Bacteria | *Mesotoga* | *prima* MesG1Ag42 | 2.98 | 442 | TransportDB |
| Bacteria | *Mycoplasma* | *pulmonis* UABCTIP | 0.96 | 103 | TransportDB |
| Bacteria | *Mycoplasma* | *putrefaciens* KS1 | 0.83 | 69 | TransportDB |
| Bacteria | *Mycoplasma* | *putrefaciens* Mput9231 | 0.86 | 70 | TransportDB |
| Bacteria | *Methylobacterium* | *radiotolerans* JCM2831 | 6.9 | 684 | TransportDB |
| Bacteria | *Mycobacterium* | *rhodesiae* NBB3 | 6.42 | 459 | TransportDB |
| Bacteria | *Melioribacter* | *roseus* P3M2 | 3.3 | 211 | TransportDB |
| Bacteria | *Meiothermus* | *ruber* DSM1279 | 3.1 | 378 | TransportDB |
| Bacteria | *Muricauda* | *ruestringensis* DSM13258 | 3.84 | 208 | TransportDB |
| Bacteria | *Meiothermus* | *silvanus* DSM9946 | 3.72 | 393 | TransportDB |
| Bacteria | *Methylocella* | *silvestris* BL2 | 4.31 | 335 | TransportDB |
| Bacteria | *Mycobacterium* | *smegmatis* JS623 | 7.22 | 497 | TransportDB |
| Bacteria | *Mycobacterium* | *smegmatis* str MC2155 | 6.99 | 713 | TransportDB |
| Bacteria | *Methylobacterium* | *s446* | 7.74 | 773 | TransportDB |
| Bacteria | *Mesorhizobium* | *sBNC1* | 4.41 | 650 | TransportDB |
| Bacteria | *Marinobacter* | *sBSs20148* | 4.06 | 556 | TransportDB |
| Bacteria | *Maribacter* | *sHTCC2170* | 3.87 | 211 | TransportDB |
| Bacteria | *Methylophaga* | *sJAM1* | 3.14 | 253 | TransportDB |
| Bacteria | *Methylophaga* | *sJAM7* | 2.75 | 228 | TransportDB |
| Bacteria | *Mycobacterium* | *sJDM601* | 4.64 | 263 | TransportDB |
| Bacteria | *Mycobacterium* | *sJLS* | 6.05 | 475 | TransportDB |
| Bacteria | *Mycobacterium* | *sKMS* | 6.26 | 463 | TransportDB |
| Bacteria | *Micromonospora* | *sL5* | 6.96 | 587 | TransportDB |
| Bacteria | *Magnetococcus* | *sMC-1* | 4.7 | 233 | TransportDB |
| Bacteria | *Mycobacterium* | *sMCS* | 5.92 | 447 | TransportDB |
| Bacteria | *Mycobacterium* | *sMOTT36Y* | 5.61 | 320 | TransportDB |
| Bacteria | *Methylovorus* | *sMP688* | 2.86 | 226 | TransportDB |
| Bacteria | *Marinomonas* | *sMWYL1* | 5.1 | 715 | TransportDB |
| Bacteria | *Microcoleus* | *sPCC7113* | 7.97 | 422 | TransportDB |
| Bacteria | *Methylocystis* | *sSC2* | 3.77 | 238 | TransportDB |
| Bacteria | *Myxococcus* | *stipitatus* DSM14675 | 10.35 | 468 | TransportDB |
| Bacteria | *Mannheimia* | *succiniciproducens* MBEL55E | 2.31 | 304 | TransportDB |
| Bacteria | *Mycoplasma* | *suis* KI3806 | 0.71 | 31 | TransportDB |
| Bacteria | *Mycoplasma* | *suis* str Illinois | 0.74 | 32 | TransportDB |
| Bacteria | *Mycoplasma* | *synoviae* 53 | 0.8 | 77 | TransportDB |
| Bacteria | *Microbacterium* | *testaceum* StLB037 | 3.98 | 507 | TransportDB |
| Bacteria | *Moorella* | *thermoacetica* ATCC39073 | 2.63 | 235 | TransportDB |
| Bacteria | *Marivirga* | *tractuosa* DSM4126 | 4.52 | 248 | TransportDB |
| Bacteria | *Mycobacterium* | *tuberculosis* 719999 | 4.42 | 268 | TransportDB |
| Bacteria | *Mycobacterium* | *tuberculosis* CASNITR204 | 4.39 | 222 | TransportDB |
| Bacteria | *Mycobacterium* | *tuberculosis* CCDC5079 | 4.41 | 266 | TransportDB |
| Bacteria | *Mycobacterium* | *tuberculosis* CCDC5180 | 4.41 | 254 | TransportDB |
| Bacteria | *Mycobacterium* | *tuberculosis* CDC1551 | 4.4 | 264 | TransportDB |
| Bacteria | *Mycobacterium* | *tuberculosis* CTRI2 | 4.4 | 265 | TransportDB |
| Bacteria | *Mycobacterium* | *tuberculosis* EAI5 | 4.39 | 254 | TransportDB |
| Bacteria | *Mycobacterium* | *tuberculosis* EAI5NITR206 | 4.39 | 251 | TransportDB |
| Bacteria | *Mycobacterium* | *tuberculosis* F11 | 4.42 | 267 | TransportDB |
| Bacteria | *Mycobacterium* | *tuberculosis* H37Ra | 4.42 | 268 | TransportDB |
| Bacteria | *Mycobacterium* | *tuberculosis* H37Rv | 4.41 | 267 | TransportDB |
| Bacteria | *Mycobacterium* | *tuberculosis* KZN1435 | 4.4 | 265 | TransportDB |
| Bacteria | *Mycobacterium* | *tuberculosis* KZN4207 | 4.39 | 264 | TransportDB |
| Bacteria | *Mycobacterium* | *tuberculosis* KZN605 | 4.4 | 263 | TransportDB |
| Bacteria | *Mycobacterium* | *tuberculosis* RGTB327 | 4.38 | 206 | TransportDB |
| Bacteria | *Mycobacterium* | *tuberculosis* RGTB423 | 4.41 | 194 | TransportDB |
| Bacteria | *Mycobacterium* | *tuberculosis* str Beijing NITR203 | 4.41 | 256 | TransportDB |
| Bacteria | *Mycobacterium* | *tuberculosis* str Erdman ATCC35801 | 4.39 | 259 | TransportDB |
| Bacteria | *Mycobacterium* | *tuberculosis* str Haarlem | 4.41 | 265 | TransportDB |
| Bacteria | *Mycobacterium* | *tuberculosis* str Haarlem NITR202 | 4.4 | 198 | TransportDB |
| Bacteria | *Mycobacterium* | *tuberculosis* UT205 | 4.42 | 262 | TransportDB |
| Bacteria | *Mycobacterium* | *ulcerans* Agy99 | 5.81 | 252 | TransportDB |
| Bacteria | *Mycobacterium* | *vanbaalenii* PYR1 | 6.49 | 506 | TransportDB |
| Bacteria | *Methylotenera* | *versatilis* 301 | 3.06 | 240 | TransportDB |
| Bacteria | *Mycoplasma* | *wenyonii* str Massachusetts | 0.65 | 38 | TransportDB |
| Bacteria | *Myxococcus* | *xanthus* DK1622 | 9.14 | 436 | TransportDB |
| Bacteria | *Mycobacterium* | *yongonense* 051390 | 5.66 | 327 | TransportDB |
| Bacteria | *Nocardiopsis* | *alba* ATCCBAA2165 | 5.85 | 543 | TransportDB |
| Bacteria | *Novosphingobium* | *aromaticivorans* DSM12444 | 4.23 | 242 | TransportDB |
| Bacteria | *Nocardia* | *brasiliensis* ATCC700358 | 9.44 | 734 | TransportDB |
| Bacteria | *Nocardia* | *cyriacigeorgica* GUH2 | 6.19 | 446 | TransportDB |
| Bacteria | *Nocardiopsis* | *dassonvillei* subsp dassonvillei DSM43111 | 6.54 | 597 | TransportDB |
| Bacteria | *Nonlabens* | *dokdonensis* DSW6 | 3.91 | 207 | TransportDB |
| Bacteria | *Nitrosomonas* | *europaea* ATCC19718 | 2.81 | 189 | TransportDB |
| Bacteria | *Nitrosomonas* | *eutropha* C91 | 2.78 | 228 | TransportDB |
| Bacteria | *Nocardia* | *farcinica* IFM10152 | 6.29 | 483 | TransportDB |
| Bacteria | *Neisseria* | *gonorrhoeae* FA1090 | 2.15 | 153 | TransportDB |
| Bacteria | *Neisseria* | *gonorrhoeae* NCCP11945 | 2.24 | 182 | TransportDB |
| Bacteria | *Neisseria* | *gonorrhoeae* TCDCNG08107 | 2.19 | 182 | TransportDB |
| Bacteria | *Nitrosococcus* | *halophilus* Nc4 | 4.15 | 292 | TransportDB |
| Bacteria | *Nitrobacter* | *hamburgensis* X14 | 5.01 | 323 | TransportDB |
| Bacteria | *Niastella* | *koreensis* GR2010 | 9.03 | 373 | TransportDB |
| Bacteria | *Neisseria* | *lactamica* 02006 | 2.22 | 160 | TransportDB |
| Bacteria | *Neisseria* | *meningitidis* 053442 | 2.15 | 155 | TransportDB |
| Bacteria | *Neisseria* | *meningitidis* 8013 | 2.28 | 166 | TransportDB |
| Bacteria | *Neisseria* | *meningitidis* alpha14 | 2.15 | 163 | TransportDB |
| Bacteria | *Neisseria* | *meningitidis* alpha710 | 2.24 | 163 | TransportDB |
| Bacteria | *Neisseria* | *meningitidis* FAM18 | 2.19 | 165 | TransportDB |
| Bacteria | *Neisseria* | *meningitidis* G2136 | 2.18 | 165 | TransportDB |
| Bacteria | *Neisseria* | *meningitidis* H4476 | 2.24 | 160 | TransportDB |
| Bacteria | *Neisseria* | *meningitidis* M01240149 | 2.22 | 160 | TransportDB |
| Bacteria | *Neisseria* | *meningitidis* M01240355 | 2.29 | 162 | TransportDB |
| Bacteria | *Neisseria* | *meningitidis* M04240196 | 2.25 | 162 | TransportDB |
| Bacteria | *Neisseria* | *meningitidis* MC58 | 2.27 | 162 | TransportDB |
| Bacteria | *Neisseria* | *meningitidis* NZ0533 | 2.25 | 160 | TransportDB |
| Bacteria | *Neisseria* | *meningitidis* WUE2594 | 2.23 | 154 | TransportDB |
| Bacteria | *Neisseria* | *meningitidis* Z2491 | 2.18 | 160 | TransportDB |
| Bacteria | *Nitrosospira* | *multiformis* ATCC25196 | 3.23 | 235 | TransportDB |
| Bacteria | *Nakamurella* | *multipartita* DSM44233 | 6.06 | 463 | TransportDB |
| Bacteria | *Nitrosococcus* | *oceani* ATCC19707 | 3.52 | 243 | TransportDB |
| Bacteria | *Nautilia* | *profundicola* AmH | 1.68 | 164 | TransportDB |
| Bacteria | *Nostoc* | *punctiforme* PCC73102 | 9.06 | 463 | TransportDB |
| Bacteria | *Neorickettsia* | *risticii* str Illinois | 0.88 | 66 | TransportDB |
| Bacteria | *Nitratifractor* | *salsuginis* DSM16511 | 2.1 | 153 | TransportDB |
| Bacteria | *Neorickettsia* | *sennetsu* str Miyayama | 0.86 | 67 | TransportDB |
| Bacteria | *Nitrosomonas* | *sAL212* | 3.34 | 234 | TransportDB |
| Bacteria | *Nitrosomonas* | *sIs79A3* | 3.78 | 213 | TransportDB |
| Bacteria | *Nocardioides* | *sJS614* | 5.29 | 459 | TransportDB |
| Bacteria | *Nostoc* | *sPCC7107* | 6.33 | 388 | TransportDB |
| Bacteria | *Nostoc* | *sPCC7120* | 7.21 | 481 | TransportDB |
| Bacteria | *Nostoc* | *sPCC7524* | 6.72 | 422 | TransportDB |
| Bacteria | *Novosphingobium* | *sPP1Y* | 5.31 | 321 | TransportDB |
| Bacteria | *Nitratiruptor* | *sSB1552* | 1.88 | 151 | TransportDB |
| Bacteria | *Natranaerobius* | *thermophilus* JWNMWNLF | 3.19 | 315 | TransportDB |
| Bacteria | *Nitrosococcus* | *watsonii* C113 | 3.37 | 248 | TransportDB |
| Bacteria | *Nitrobacter* | *winogradskyi* Nb255 | 3.4 | 215 | TransportDB |
| Bacteria | *Oscillatoria* | *acuminata* PCC6304 | 7.8 | 329 | TransportDB |
| Bacteria | *Octadecabacter* | *antarcticus* 307 | 4.88 | 558 | TransportDB |
| Bacteria | *Ochrobactrum* | *anthropi* ATCC49188 | 5.21 | 855 | TransportDB |
| Bacteria | *Octadecabacter* | *arcticus* 238 | 5.48 | 525 | TransportDB |
| Bacteria | *Oligotropha* | *carboxidovorans* OM4 | 3.84 | 401 | TransportDB |
| Bacteria | *Oligotropha* | *carboxidovorans* OM5 | 3.9 | 409 | TransportDB |
| Bacteria | *Owenweeksia* | *hongkongensis* DSM17368 | 4.0 | 208 | TransportDB |
| Bacteria | *Oceanobacillus* | *iheyensis* HTE831 | 3.63 | 505 | TransportDB |
| Bacteria | *Oscillatoria* | *nigro-viridis* PCC7112 | 8.27 | 377 | TransportDB |
| Bacteria | *Oenococcus* | *oeni* PSU1 | 1.78 | 266 | TransportDB |
| Bacteria | *Oceanithermus* | *profundus* DSM14977 | 2.44 | 282 | TransportDB |
| Bacteria | *Ornithobacterium* | *rhinotracheale* DSM15997 | 2.4 | 139 | TransportDB |
| Bacteria | *Oceanimonas* | *sGK1* | 3.53 | 439 | TransportDB |
| Bacteria | *Odoribacter* | *splanchnicus* DSM20712 | 4.39 | 231 | TransportDB |
| Bacteria | *Opitutus* | *terrae* PB901 | 5.96 | 348 | TransportDB |
| Bacteria | *Orientia* | *tsutsugamushi* str Boryong | 2.13 | 83 | TransportDB |
| Bacteria | *Orientia* | *tsutsugamushi* str Ikeda | 2.01 | 120 | TransportDB |
| Bacteria | *Olsenella* | *uli* DSM7084 | 2.05 | 250 | TransportDB |
| Bacteria | *Oscillibacter* | *valericigenes* Sjm1820 | 4.47 | 412 | TransportDB |
| Bacteria | *Onion* | *yellows phytoplasma* OYM | 0.85 | 64 | TransportDB |
| Bacteria | *Parachlamydia* | *acanthamoebae* UV7 | 3.07 | 208 | TransportDB |
| Bacteria | *Propionibacterium* | *acidipropionici* ATCC4875 | 3.66 | 431 | TransportDB |
| Bacteria | *Propionibacterium* | *acnes* 266 | 2.49 | 321 | TransportDB |
| Bacteria | *Propionibacterium* | *acnes* 6609 | 2.56 | 323 | TransportDB |
| Bacteria | *Propionibacterium* | *acnes* ATCC11828 | 2.49 | 314 | TransportDB |
| Bacteria | *Propionibacterium* | *acnes* C1 | 2.52 | 327 | TransportDB |
| Bacteria | *Propionibacterium* | *acnes* HL096PA1 | 2.55 | 311 | TransportDB |
| Bacteria | *Propionibacterium* | *acnes* KPA171202 | 2.56 | 324 | TransportDB |
| Bacteria | *Propionibacterium* | *acnes* SK137 | 2.5 | 324 | TransportDB |
| Bacteria | *Propionibacterium* | *acnes* Type IA2 Pacn17 | 2.52 | 316 | TransportDB |
| Bacteria | *Propionibacterium* | *acnes* Type IA2 Pacn31 | 2.5 | 315 | TransportDB |
| Bacteria | *Propionibacterium* | *acnes* Type IA2 Pacn33 | 2.49 | 315 | TransportDB |
| Bacteria | *Pseudomonas* | *aeruginosa* B13633 | 6.42 | 697 | TransportDB |
| Bacteria | *Pseudomonas* | *aeruginosa* c7447m | 6.26 | 707 | TransportDB |
| Bacteria | *Pseudomonas* | *aeruginosa* DK2 | 6.4 | 702 | TransportDB |
| Bacteria | *Pseudomonas* | *aeruginosa* LES431 | 6.55 | 712 | TransportDB |
| Bacteria | *Pseudomonas* | *aeruginosa* LESB58 | 6.6 | 711 | TransportDB |
| Bacteria | *Pseudomonas* | *aeruginosa* M18 | 6.33 | 703 | TransportDB |
| Bacteria | *Pseudomonas* | *aeruginosa* MTB1 | 6.58 | 689 | TransportDB |
| Bacteria | *Pseudomonas* | *aeruginosa* NCGM2S1 | 6.76 | 709 | TransportDB |
| Bacteria | *Pseudomonas* | *aeruginosa* PA1 | 6.53 | 706 | TransportDB |
| Bacteria | *Pseudomonas* | *aeruginosa* PA1R | 6.31 | 682 | TransportDB |
| Bacteria | *Pseudomonas* | *aeruginosa* PA7 | 6.59 | 681 | TransportDB |
| Bacteria | *Pseudomonas* | *aeruginosa* PAO1 | 6.26 | 706 | TransportDB |
| Bacteria | *Pseudomonas* | *aeruginosa* PAO1VE13 | 6.27 | 707 | TransportDB |
| Bacteria | *Pseudomonas* | *aeruginosa* PAO1VE2 | 6.27 | 708 | TransportDB |
| Bacteria | *Pseudomonas* | *aeruginosa* PAO581 | 6.04 | 686 | TransportDB |
| Bacteria | *Pseudomonas* | *aeruginosa* RP73 | 6.34 | 697 | TransportDB |
| Bacteria | *Pseudomonas* | *aeruginosa* SCV20265 | 6.73 | 716 | TransportDB |
| Bacteria | *Pseudomonas* | *aeruginosa* UCBPPPA14 | 6.54 | 705 | TransportDB |
| Bacteria | *Prosthecochloris* | *aestuarii* DSM271 | 2.58 | 205 | TransportDB |
| Bacteria | *Paracoccus* | *aminophilus* JCM7686 | 4.87 | 726 | TransportDB |
| Bacteria | *Pantoea* | *ananatis* AJ13355 | 4.88 | 686 | TransportDB |
| Bacteria | *Pantoea* | *ananatis* LMG20103 | 4.7 | 683 | TransportDB |
| Bacteria | *Pantoea* | *ananatis* LMG5342 | 4.91 | 680 | TransportDB |
| Bacteria | *Pantoea* | *ananatis* PA13 | 4.87 | 689 | TransportDB |
| Bacteria | *Psychrobacter* | *arcticus* 2734 | 2.65 | 174 | TransportDB |
| Bacteria | *Porphyromonas* | *asaccharolytica* DSM20707 | 2.19 | 108 | TransportDB |
| Bacteria | *Photorhabdus* | *asymbiotica* | 5.09 | 405 | TransportDB |
| Bacteria | *Pseudoalteromonas* | *atlantica T6c* | 5.19 | 363 | TransportDB |
| Bacteria | *Pectobacterium* | *atrosepticum* SCRI1043 | 5.06 | 699 | TransportDB |
| Bacteria | *Propionibacterium* | *avidum* 44067 | 2.53 | 309 | TransportDB |
| Bacteria | *Parvularcula* | *bermudensis* HTCC2503 | 2.9 | 225 | TransportDB |
| Bacteria | *Planctomyces* | *brasiliensis* DSM5305 | 6.01 | 268 | TransportDB |
| Bacteria | *Pseudomonas* | *brassicacearum* subsp brassicacearum NFM421 | 6.84 | 783 | TransportDB |
| Bacteria | *Pelobacter* | *carbinolicus* DSM2380 | 3.67 | 295 | TransportDB |
| Bacteria | *Pectobacterium* | *carotovorum* subsp carotovorum PC1 | 4.86 | 720 | TransportDB |
| Bacteria | *Pectobacterium* | *carotovorum* subsp carotovorum PCC21 | 4.84 | 703 | TransportDB |
| Bacteria | *Pediococcus* | *claussenii* ATCCBAA344 | 1.98 | 245 | TransportDB |
| Bacteria | *Psychrobacter* | *cryohalolentis* K5 | 3.1 | 261 | TransportDB |
| Bacteria | *Pseudomonas* | *denitrificans* ATCC13867 | 5.7 | 599 | TransportDB |
| Bacteria | *Paracoccus* | *denitrificans* PD1222 | 5.24 | 801 | TransportDB |
| Bacteria | *Prevotella* | *dentalis* DSM3688 | 3.35 | 152 | TransportDB |
| Bacteria | *Prevotella* | *denticola* F0289 | 2.94 | 136 | TransportDB |
| Bacteria | *Pseudonocardia* | *dioxanivorans* CB1190 | 7.44 | 560 | TransportDB |
| Bacteria | *Parabacteroides* | *distasonis* ATCC8503 | 4.81 | 295 | TransportDB |
| Bacteria | *Pseudomonas* | *entomophila* L48 | 5.89 | 611 | TransportDB |
| Bacteria | *Pseudomonas* | *fluorescens* A506 | 6.02 | 688 | TransportDB |
| Bacteria | *Pseudomonas* | *fluorescens* F113 | 6.85 | 768 | TransportDB |
| Bacteria | *Pseudomonas* | *fluorescens* Pf01 | 6.44 | 709 | TransportDB |
| Bacteria | *Pseudomonas* | *fluorescens* Pf-5 | 7.07 | 767 | TransportDB |
| Bacteria | *Pseudomonas* | *fluorescens* SBW25 | 6.72 | 807 | TransportDB |
| Bacteria | *Propionibacterium* | *freudenreichii* subsp shermanii CIRMBIA1 | 2.62 | 261 | TransportDB |
| Bacteria | *Pseudomonas* | *fulva* 12X | 4.92 | 606 | TransportDB |
| Bacteria | *Phaeobacter* | *gallaeciensis* 210 | 4.16 | 509 | TransportDB |
| Bacteria | *Phaeobacter* | *gallaeciensis* DSM17395CIP105210 | 4.23 | 505 | TransportDB |
| Bacteria | *Phaeobacter* | *gallaeciensis* DSM26640 | 4.54 | 569 | TransportDB |
| Bacteria | *Polymorphum* | *gilvum* SL003B26A1 | 4.72 | 581 | TransportDB |
| Bacteria | *Porphyromonas* | *gingivalis* ATCC33277 | 2.35 | 128 | TransportDB |
| Bacteria | *Porphyromonas* | *gingivalis* TDC60 | 2.34 | 127 | TransportDB |
| Bacteria | *Porphyromonas* | *gingivalis* W83 | 2.34 | 113 | TransportDB |
| Bacteria | *Pseudoalteromonas* | *haloplanktis* TAC125 | 3.85 | 295 | TransportDB |
| Bacteria | *Pelagibacterium* | *halotolerans* B2 | 3.95 | 649 | TransportDB |
| Bacteria | *Pedobacter* | *heparinus* DSM2366 | 5.17 | 257 | TransportDB |
| Bacteria | *Psychromonas* | *ingrahamii* 37 | 4.56 | 479 | TransportDB |
| Bacteria | *Prevotella* | *intermedia* 17 | 2.7 | 111 | TransportDB |
| Bacteria | *Paenibacillus* | *larvae* subsp larvae DSM25430 | 4.06 | 391 | TransportDB |
| Bacteria | *Parvibaculum* | *lavamentivorans* DS1 | 3.91 | 306 | TransportDB |
| Bacteria | *Planctomyces* | *limnophilus* DSM3776 | 5.46 | 223 | TransportDB |
| Bacteria | *Photorhabdus* | *luminescens* subsp laumondii TTO1 | 5.69 | 417 | TransportDB |
| Bacteria | *Pelodictyon* | *luteolum* DSM 273 | 2.36 | 183 | TransportDB |
| Bacteria | *Persephonella* | *marina* EXH1 | 1.98 | 144 | TransportDB |
| Bacteria | *Prochlorococcus* | *marinus* str AS9601 | 1.67 | 103 | TransportDB |
| Bacteria | *Prochlorococcus* | *marinus* str MIT9211 | 1.69 | 107 | TransportDB |
| Bacteria | *Prochlorococcus* | *marinus* str MIT9215 | 1.74 | 102 | TransportDB |
| Bacteria | *Prochlorococcus* | *marinus* str MIT9301 | 1.64 | 109 | TransportDB |
| Bacteria | *Prochlorococcus* | *marinus* str MIT9303 | 2.68 | 177 | TransportDB |
| Bacteria | *Prochlorococcus* | *marinus* str MIT9312 | 1.71 | 115 | TransportDB |
| Bacteria | *Prochlorococcus* | *marinus* str MIT9313 | 2.41 | 162 | TransportDB |
| Bacteria | *Prochlorococcus* | *marinus* str MIT9515 | 1.7 | 105 | TransportDB |
| Bacteria | *Prochlorococcus* | *marinus* str NATL1A | 1.86 | 118 | TransportDB |
| Bacteria | *Prochlorococcus* | *marinus* str NATL2A | 1.84 | 117 | TransportDB |
| Bacteria | *Prochlorococcus* | *marinus* subsp marinus str CCMP1375 | 1.75 | 107 | TransportDB |
| Bacteria | *Prochlorococcus* | *marinus* subsp pastoris str CCMP1986 | 1.66 | 106 | TransportDB |
| Bacteria | *Prevotella* | *melaninogenica* ATCC25845 | 3.17 | 125 | TransportDB |
| Bacteria | *Pseudomonas* | *mendocina* NK01 | 5.43 | 608 | TransportDB |
| Bacteria | *Pseudomonas* | *mendocina* ymp | 5.07 | 565 | TransportDB |
| Bacteria | *Phycisphaera* | *mikurensis* NBRC102666 | 3.88 | 237 | TransportDB |
| Bacteria | *Proteus* | *mirabilis* BB2000 | 3.85 | 429 | TransportDB |
| Bacteria | *Proteus* | *mirabilis* HI4320 | 4.1 | 430 | TransportDB |
| Bacteria | *Petrotoga* | *mobilis* SJ95 | 2.17 | 303 | TransportDB |
| Bacteria | *Pseudomonas* | *monteilii* SB3078 | 6.0 | 648 | TransportDB |
| Bacteria | *Pseudomonas* | *monteilii* SB3101 | 5.95 | 648 | TransportDB |
| Bacteria | *Paenibacillus* | *mucilaginosus* 3016 | 8.74 | 1020 | TransportDB |
| Bacteria | *Paenibacillus* | *mucilaginosus* K02 | 8.82 | 1031 | TransportDB |
| Bacteria | *Paenibacillus* | *mucilaginosus* KNP414 | 8.66 | 1038 | TransportDB |
| Bacteria | *Pasteurella* | *multocida* 36950 | 2.35 | 310 | TransportDB |
| Bacteria | *Pasteurella* | *multocida* subsp multocida str 3480 | 2.38 | 320 | TransportDB |
| Bacteria | *Pasteurella* | *multocida* subsp multocida str HN06 | 2.41 | 316 | TransportDB |
| Bacteria | *Pasteurella* | *multocida* subsp multocida str Pm70 | 2.26 | 317 | TransportDB |
| Bacteria | *Polaromonas* | *naphthalenivorans* CJ2 | 5.37 | 517 | TransportDB |
| Bacteria | *Polynucleobacter* | *necessarius* subsp asymbioticus QLWP1DMWA1 | 2.16 | 215 | TransportDB |
| Bacteria | *Polynucleobacter* | *necessarius* subsp necessarius STIR1 | 1.56 | 107 | TransportDB |
| Bacteria | *Pediococcus* | *pentosaceus* ATCC25745 | 1.83 | 242 | TransportDB |
| Bacteria | *Pediococcus* | *pentosaceus* SL4 | 1.79 | 228 | TransportDB |
| Bacteria | *Pelodictyon* | *phaeoclathratiforme* BU1 | 3.02 | 179 | TransportDB |
| Bacteria | *Pandoraea* | *pnomenusa* 3kgm | 5.44 | 694 | TransportDB |
| Bacteria | *Pseudomonas* | *poae* RE1114 | 5.51 | 605 | TransportDB |
| Bacteria | *Paenibacillus* | *polymyxa* CR1 | 6.02 | 753 | TransportDB |
| Bacteria | *Paenibacillus* | *polymyxa* E681 | 5.39 | 647 | TransportDB |
| Bacteria | *Paenibacillus* | *polymyxa* M1 | 6.23 | 712 | TransportDB |
| Bacteria | *Paenibacillus* | *polymyxa* SC2 | 6.24 | 708 | TransportDB |
| Bacteria | *Photobacterium* | *profundum* SS9 | 6.4 | 660 | TransportDB |
| Bacteria | *Paludibacter* | *propionicigenes* WB4 | 3.69 | 200 | TransportDB |
| Bacteria | *Propionibacterium* | *propionicum* F0230a | 3.45 | 418 | TransportDB |
| Bacteria | *Pelobacter* | *propionicus* DSM2379 | 4.24 | 332 | TransportDB |
| Bacteria | *Pseudomonas* | *protegens* CHA0 | 6.87 | 781 | TransportDB |
| Bacteria | *Pseudomonas* | *protegens* Pf5 | 7.07 | 780 | TransportDB |
| Bacteria | *Pseudomonas* | *putida* BIRD1 | 5.73 | 665 | TransportDB |
| Bacteria | *Pseudomonas* | *putida* DOTT1E | 6.26 | 686 | TransportDB |
| Bacteria | *Pseudomonas* | *putida* F1 | 5.96 | 667 | TransportDB |
| Bacteria | *Pseudomonas* | *putida* GB1 | 6.08 | 711 | TransportDB |
| Bacteria | *Pseudomonas* | *putida* H8234 | 6.87 | 754 | TransportDB |
| Bacteria | *Pseudomonas* | *putida* HB3267 | 5.96 | 659 | TransportDB |
| Bacteria | *Pseudomonas* | *putida* KT2440 | 6.18 | 677 | TransportDB |
| Bacteria | *Pseudomonas* | *putida* NBRC14164 | 6.16 | 721 | TransportDB |
| Bacteria | *Pseudomonas* | *putida* ND6 | 6.09 | 679 | TransportDB |
| Bacteria | *Pseudomonas* | *putida* S16 | 5.98 | 660 | TransportDB |
| Bacteria | *Pseudomonas* | *putida* W619 | 5.77 | 636 | TransportDB |
| Bacteria | *Pseudomonas* | *resinovorans* NBRC106553 | 6.48 | 697 | TransportDB |
| Bacteria | *Prevotella* | *ruminicola* 23 | 3.62 | 166 | TransportDB |
| Bacteria | *Pedobacter* | *saltans* DSM12145 | 4.64 | 227 | TransportDB |
| Bacteria | *Polynucleobacter* | *sp QLW-P1DMWA-1* | 2.16 | 227 | TransportDB |
| Bacteria | *Pantoea* | *sAt9b* | 6.31 | 1028 | TransportDB |
| Bacteria | *Prochlorococcus* | *sCC9311* | 2.61 | 198 | TransportDB |
| Bacteria | *Prochlorococcus* | *sCC9605* (oligotrophic) | 2.51 | 174 | TransportDB |
| Bacteria | *Prochlorococcus* | *sCC9902* (coastal) | 2.23 | 158 | TransportDB |
| Bacteria | *Psychromonas* | *sCNPT3* | 3.05 | 329 | TransportDB |
| Bacteria | *Pseudovibrio* | *sFOBEG1* | 5.92 | 765 | TransportDB |
| Bacteria | *Psychrobacter* | *sG* | 3.11 | 274 | TransportDB |
| Bacteria | *Paenibacillus* | *sJDR2* | 7.18 | 1031 | TransportDB |
| Bacteria | *Polaromonas* | *sJS666* | 5.9 | 765 | TransportDB |
| Bacteria | *Polaribacter* | *sMED152* | 2.96 | 169 | TransportDB |
| Bacteria | *Pseudogulbenkiania* | *sNH8B* | 4.33 | 464 | TransportDB |
| Bacteria | *Prevotella* | *soraltaxon299* str F0039 | 2.48 | 116 | TransportDB |
| Bacteria | *Pleurocapsa* | *sPCC7327* | 4.99 | 334 | TransportDB |
| Bacteria | *Pseudanabaena* | *sPCC7367* | 4.89 | 276 | TransportDB |
| Bacteria | *Psychrobacter* | *sPRwf1* | 3.0 | 254 | TransportDB |
| Bacteria | *Pandoraea* | *sRB44* | 5.39 | 689 | TransportDB |
| Bacteria | *Pectobacterium* | *sSCC3193* | 5.16 | 700 | TransportDB |
| Bacteria | *Pseudoalteromonas* | *sSM9913* | 4.04 | 303 | TransportDB |
| Bacteria | *Pusillimonas* | *sT77* | 3.92 | 592 | TransportDB |
| Bacteria | *Pseudomonas* | *sTKP* | 7.01 | 851 | TransportDB |
| Bacteria | *Pseudomonas* | *sUW4* | 6.18 | 695 | TransportDB |
| Bacteria | *Pseudomonas* | *sVLB120* | 5.97 | 618 | TransportDB |
| Bacteria | *Prochlorococcus* | *sWH 7803* | 2.37 | 213 | TransportDB |
| Bacteria | *Prochlorococcus* | *sWH8102* | 2.43 | 180 | TransportDB |
| Bacteria | *Paenibacillus* | *sY412MC10* | 7.12 | 1095 | TransportDB |
| Bacteria | *Pseudoxanthomonas* | *spadix* BDa59 | 3.45 | 254 | TransportDB |
| Bacteria | *Pirellula* | *staleyi* DSM6068 | 6.2 | 238 | TransportDB |
| Bacteria | *Plautia* | *stali symbiont* | 4.09 | 762 | TransportDB |
| Bacteria | *Providencia* | *stuartii* MRSN2154 | 4.4 | 484 | TransportDB |
| Bacteria | *Pseudomonas* | *stutzeri* A1501 | 4.57 | 490 | TransportDB |
| Bacteria | *Pseudomonas* | *stutzeri* ATCC17588LMG11199 | 4.55 | 488 | TransportDB |
| Bacteria | *Pseudomonas* | *stutzeri* CCUG29243 | 4.71 | 495 | TransportDB |
| Bacteria | *Pseudomonas* | *stutzeri* DSM10701 | 4.17 | 447 | TransportDB |
| Bacteria | *Pseudomonas* | *stutzeri* DSM4166 | 4.69 | 536 | TransportDB |
| Bacteria | *Pseudomonas* | *stutzeri* RCH2 | 4.6 | 490 | TransportDB |
| Bacteria | *Pseudoxanthomonas* | *suwonensis* 111 | 3.42 | 259 | TransportDB |
| Bacteria | *Pseudomonas* | *syringae pvphaseolicola* 1448A | 6.11 | 670 | TransportDB |
| Bacteria | *Pseudomonas* | *syringae pvsyringae* B728a | 6.09 | 668 | TransportDB |
| Bacteria | *Pseudomonas* | *syringae pvtomato* str DC3000 | 6.54 | 673 | TransportDB |
| Bacteria | *Paenibacillus* | *terrae* HPL003 | 6.08 | 788 | TransportDB |
| Bacteria | *Pelotomaculum* | *thermopropionicum* SI | 3.03 | 222 | TransportDB |
| Bacteria | *Psychroflexus* | *torquis* ATCC700755 | 4.32 | 212 | TransportDB |
| Bacteria | *Pantoea* | *vagans* C91 | 4.89 | 698 | TransportDB |
| Bacteria | *Pectobacterium* | *wasabiae* WPP163 | 5.06 | 675 | TransportDB |
| Bacteria | *Phenylobacterium* | *zucineum* HLK1 | 4.38 | 303 | TransportDB |
| Bacteria | *Rickettsia* | *africae* ESF5 | 1.29 | 104 | TransportDB |
| Bacteria | *Rickettsia* | *akari* str Hartford | 1.23 | 106 | TransportDB |
| Bacteria | *Ruminococcus* | *albus 7* | 4.48 | 236 | TransportDB |
| Bacteria | *Riemerella* | *anatipestifer* ATCC11845DSM15868 | 2.16 | 112 | TransportDB |
| Bacteria | *Riemerella* | *anatipestifer* RACH1 | 2.31 | 111 | TransportDB |
| Bacteria | *Riemerella* | *anatipestifer* RACH2 | 2.17 | 111 | TransportDB |
| Bacteria | *Riemerella* | *anatipestifer* RAGD | 2.17 | 108 | TransportDB |
| Bacteria | *Rahnella* | *aquatilis* CIP7865ATCC33071 | 5.45 | 820 | TransportDB |
| Bacteria | *Rahnella* | *aquatilis* HX2 | 5.66 | 858 | TransportDB |
| Bacteria | *Rickettsia* | *australis* str Cutlack | 1.32 | 112 | TransportDB |
| Bacteria | *Rhodopirellula* | *baltica* SH1 | 7.15 | 308 | TransportDB |
| Bacteria | *Rickettsia* | *bellii* OSU85389 | 1.53 | 118 | TransportDB |
| Bacteria | *Rickettsia* | *bellii* RML369C | 1.52 | 134 | TransportDB |
| Bacteria | *Robiginitalea* | *biformata* HTCC2501 | 3.53 | 202 | TransportDB |
| Bacteria | *Ruminococcus* | *bromii* L263 | 2.25 | 147 | TransportDB |
| Bacteria | *Rickettsia* | *canadensis* str CA410 | 1.15 | 102 | TransportDB |
| Bacteria | *Rickettsia* | *canadensis* str McKiel | 1.16 | 97 | TransportDB |
| Bacteria | *Rhodobacter* | *capsulatus* SB1003 | 3.87 | 468 | TransportDB |
| Bacteria | *Roseiflexus* | *castenholzii* DSM13941 | 5.72 | 397 | TransportDB |
| Bacteria | *Rhodospirillum* | *centenum* SW | 4.36 | 339 | TransportDB |
| Bacteria | *Ruminococcus* | *champanellensis* 18P13 | 2.57 | 159 | TransportDB |
| Bacteria | *Rickettsia* | *conorii* str Malish7 | 1.27 | 125 | TransportDB |
| Bacteria | *Roseobacter* | *denitrificans OCh114* | 4.33 | 579 | TransportDB |
| Bacteria | *Rothia* | *dentocariosa* ATCC17931 | 2.51 | 222 | TransportDB |
| Bacteria | *Rhodococcus* | *equi* 103S | 5.04 | 468 | TransportDB |
| Bacteria | *Rhodococcus* | *erythropolis* CCM2595 | 6.37 | 706 | TransportDB |
| Bacteria | *Rhodococcus* | *erythropolis* PR4 | 6.9 | 718 | TransportDB |
| Bacteria | *Rhizobium* | *etli bvmimosae* str Mim1 | 7.2 | 1049 | TransportDB |
| Bacteria | *Rhizobium* | *etli* CFN42 | 6.53 | 893 | TransportDB |
| Bacteria | *Rhizobium* | *etli* CIAT652 | 6.45 | 875 | TransportDB |
| Bacteria | *Ralstonia* | *eutropha* H16 | 7.42 | 839 | TransportDB |
| Bacteria | *Ralstonia* | *eutropha* JMP134 | 7.26 | 791 | TransportDB |
| Bacteria | *Rickettsia* | *felis* URRWXCal2 | 1.59 | 132 | TransportDB |
| Bacteria | *Rhodoferax* | *ferrireducens* T118 | 4.97 | 566 | TransportDB |
| Bacteria | *Rubrivivax* | *gelatinosus* IL144 | 5.04 | 482 | TransportDB |
| Bacteria | *Rickettsia* | *heilongjiangensis* 054 | 1.28 | 120 | TransportDB |
| Bacteria | *Roseburia* | *hominis* A2183 | 3.59 | 376 | TransportDB |
| Bacteria | *Roseburia* | *intestinalis* M501 | 4.14 | 379 | TransportDB |
| Bacteria | *Roseburia* | *intestinalis* XB6B4 | 4.29 | 380 | TransportDB |
| Bacteria | *Rickettsia* | *japonica* YH | 1.28 | 102 | TransportDB |
| Bacteria | *Rhodococcus* | *jostii* RHA1 | 9.7 | 804 | TransportDB |
| Bacteria | *Rhizobium* | *leguminosarum bvtrifolii* WSM1325 | 7.42 | 1087 | TransportDB |
| Bacteria | *Rhizobium* | *leguminosarum bvtrifolii* WSM2304 | 6.87 | 1030 | TransportDB |
| Bacteria | *Rhizobium* | *leguminosarum bvviciae3841* | 7.75 | 1169 | TransportDB |
| Bacteria | *Roseobacter* | *litoralis* Och149 | 4.75 | 675 | TransportDB |
| Bacteria | *Rhodothermus* | *marinus* DSM4252 | 3.39 | 208 | TransportDB |
| Bacteria | *Rhodothermus* | *marinus* SG05JP17172 | 3.33 | 197 | TransportDB |
| Bacteria | *Rickettsia* | *massiliae* MTU5 | 1.38 | 104 | TransportDB |
| Bacteria | *Rickettsia* | *massiliae* str AZT80 | 1.28 | 104 | TransportDB |
| Bacteria | *Rickettsia* | *montanensis* str OSU85930 | 1.28 | 113 | TransportDB |
| Bacteria | *Rothia* | *mucilaginosa* DY18 | 2.26 | 206 | TransportDB |
| Bacteria | *Rhodococcus* | *opacus* B4 | 8.83 | 773 | TransportDB |
| Bacteria | *Raoultella* | *ornithinolytica* B6 | 5.4 | 929 | TransportDB |
| Bacteria | *Rhodopseudomonas* | *palustris* BisA53 | 5.51 | 486 | TransportDB |
| Bacteria | *Rhodopseudomonas* | *palustris* BisB18 | 5.51 | 541 | TransportDB |
| Bacteria | *Rhodopseudomonas* | *palustris* BisB5 | 4.89 | 525 | TransportDB |
| Bacteria | *Rhodopseudomonas* | *palustris* CGA009 | 5.47 | 629 | TransportDB |
| Bacteria | *Rhodopseudomonas* | *palustris* DX1 | 5.4 | 600 | TransportDB |
| Bacteria | *Rhodopseudomonas* | *palustris* HaA2 | 5.33 | 596 | TransportDB |
| Bacteria | *Rhodopseudomonas* | *palustris* TIE1 | 5.74 | 641 | TransportDB |
| Bacteria | *Rickettsia* | *parkeri* str Portsmouth | 1.3 | 118 | TransportDB |
| Bacteria | *Rickettsia* | *peacockii* str Rustic | 1.31 | 96 | TransportDB |
| Bacteria | *Rickettsia* | *philipii* str 364D | 1.29 | 120 | TransportDB |
| Bacteria | *Rhodospirillum* | *photometricum* DSM122 | 3.88 | 371 | TransportDB |
| Bacteria | *Ralstonia* | *pickettii* 12D | 5.69 | 560 | TransportDB |
| Bacteria | *Ralstonia* | *pickettii* 12J | 5.33 | 561 | TransportDB |
| Bacteria | *Ralstonia* | *pickettii* DTP0602 | 8.13 | 936 | TransportDB |
| Bacteria | *Ruegeria* | *pomeroyi* DSS3 | 4.6 | 602 | TransportDB |
| Bacteria | *Rickettsia* | *prowazekii* str Breinl | 1.11 | 99 | TransportDB |
| Bacteria | *Rickettsia* | *prowazekii* str BuV67CWPP | 1.11 | 97 | TransportDB |
| Bacteria | *Rickettsia* | *prowazekii* str Chernikova | 1.11 | 98 | TransportDB |
| Bacteria | *Rickettsia* | *prowazekii* str Dachau | 1.11 | 96 | TransportDB |
| Bacteria | *Rickettsia* | *prowazekii* str GvV257 | 1.11 | 96 | TransportDB |
| Bacteria | *Rickettsia* | *prowazekii* str Katsinyian | 1.11 | 97 | TransportDB |
| Bacteria | *Rickettsia* | *prowazekii* str MadridE | 1.11 | 98 | TransportDB |
| Bacteria | *Rickettsia* | *prowazekii* str NMRCMadridE | 1.11 | 99 | TransportDB |
| Bacteria | *Rickettsia* | *prowazekii* str Rp22 | 1.11 | 98 | TransportDB |
| Bacteria | *Rickettsia* | *prowazekii* str RpGvF24 | 1.11 | 97 | TransportDB |
| Bacteria | *Rhodococcus* | *pyridinivorans* SB3094 | 5.59 | 464 | TransportDB |
| Bacteria | *Rickettsia* | *rhipicephali* str 37female6CWPP | 1.31 | 115 | TransportDB |
| Bacteria | *Rickettsia* | *rickettsii* str Arizona | 1.27 | 117 | TransportDB |
| Bacteria | *Rickettsia* | *rickettsii* str Brazil | 1.26 | 116 | TransportDB |
| Bacteria | *Rickettsia* | *rickettsii* str Colombia | 1.27 | 118 | TransportDB |
| Bacteria | *Rickettsia* | *rickettsii* str Hauke | 1.27 | 118 | TransportDB |
| Bacteria | *Rickettsia* | *rickettsii* str Hino | 1.27 | 118 | TransportDB |
| Bacteria | *Rickettsia* | *rickettsii* str Hlp2 | 1.27 | 113 | TransportDB |
| Bacteria | *Rickettsia* | *rickettsii* str Iowa | 1.27 | 112 | TransportDB |
| Bacteria | *Rickettsia* | *rickettsii* str Sheila Smith | 1.26 | 107 | TransportDB |
| Bacteria | *Rhodospirillum* | *rubrum* ATCC11170 | 4.41 | 498 | TransportDB |
| Bacteria | *Rhodospirillum* | *rubrum* F11 | 4.35 | 496 | TransportDB |
| Bacteria | *Renibacterium* | *salmoninarum* ATCC33209 | 3.16 | 403 | TransportDB |
| Bacteria | *Runella* | *slithyformis* DSM19594 | 6.92 | 302 | TransportDB |
| Bacteria | *Rickettsia* | *slovaca* 13B | 1.28 | 100 | TransportDB |
| Bacteria | *Rickettsia* | *slovaca* str DCWPP | 1.28 | 123 | TransportDB |
| Bacteria | *Ralstonia* | *solanacearum* CFBP2957 | 3.42 | 313 | TransportDB |
| Bacteria | *Ralstonia* | *solanacearum* CMR15 | 5.59 | 499 | TransportDB |
| Bacteria | *Ralstonia* | *solanacearum* FQY_4 | 5.81 | 508 | TransportDB |
| Bacteria | *Ralstonia* | *solanacearum* GMI1000 | 5.81 | 511 | TransportDB |
| Bacteria | *Ralstonia* | *solanacearum* Po82 | 5.43 | 501 | TransportDB |
| Bacteria | *Ralstonia* | *solanacearum* PSI07 | 5.61 | 515 | TransportDB |
| Bacteria | *Rhodococcus* | *sp RHA1* | 7.8 | 834 | TransportDB |
| Bacteria | *Rhodanobacter* | *s2APBS1* | 4.23 | 301 | TransportDB |
| Bacteria | *Rhizobium* | *sIRBG74* | 5.46 | 917 | TransportDB |
| Bacteria | *Rivularia* | *sPCC7116* | 8.73 | 466 | TransportDB |
| Bacteria | *Roseiflexus* | *sRS1* | 5.8 | 391 | TransportDB |
| Bacteria | *Ruminococcus* | *sSR15* | 3.55 | 350 | TransportDB |
| Bacteria | *Ruegeria* | *sTM1040* | 4.15 | 502 | TransportDB |
| Bacteria | *Rahnella* | *sY9602* | 5.61 | 859 | TransportDB |
| Bacteria | *Rhodobacter* | *sphaeroides* 241 | 4.6 | 575 | TransportDB |
| Bacteria | *Rhodobacter* | *sphaeroides* ATCC17025 | 4.56 | 526 | TransportDB |
| Bacteria | *Rhodobacter* | *sphaeroides* ATCC17029 | 4.49 | 592 | TransportDB |
| Bacteria | *Rhodobacter* | *sphaeroides* KD131 | 4.71 | 629 | TransportDB |
| Bacteria | *Ramlibacter* | *tataouinensis* TTB310 | 4.07 | 480 | TransportDB |
| Bacteria | *Rhizobium* | *tropici* CIAT899 | 6.69 | 964 | TransportDB |
| Bacteria | *Rickettsia* | *typhi* str B9991CWPP | 1.11 | 97 | TransportDB |
| Bacteria | *Rickettsia* | *typhi* str TH1527 | 1.11 | 97 | TransportDB |
| Bacteria | *Rickettsia* | *typhi* str Wilmington | 1.11 | 96 | TransportDB |
| Bacteria | *Rhodomicrobium* | *vannielii* ATCC17100 | 4.01 | 322 | TransportDB |
| Bacteria | *Rubrobacter* | *xylanophilus* DSM9941 | 3.23 | 361 | TransportDB |
| Bacteria | *Singulisphaera* | *acidiphila* DSM18658 | 9.76 | 355 | TransportDB |
| Bacteria | *Syntrophus* | *aciditrophicus* SB | 3.18 | 244 | TransportDB |
| Bacteria | *Sulfobacillus* | *acidophilus* DSM10332 | 3.56 | 333 | TransportDB |
| Bacteria | *Sulfobacillus* | *acidophilus* TPY | 3.55 | 342 | TransportDB |
| Bacteria | *Spirochaeta* | *africana* DSM8902 | 3.29 | 311 | TransportDB |
| Bacteria | *Streptococcus* | *agalactiae* | 1.84 | 246 | TransportDB |
| Bacteria | *Streptococcus* | *agalactiae* 09mas018883 | 2.14 | 301 | TransportDB |
| Bacteria | *Streptococcus* | *agalactiae* 2603VR | 2.16 | 299 | TransportDB |
| Bacteria | *Streptococcus* | *agalactiae* A909 | 2.13 | 301 | TransportDB |
| Bacteria | *Streptococcus* | *agalactiae* GD201008001 | 2.06 | 301 | TransportDB |
| Bacteria | *Streptococcus* | *agalactiae* ILRI005 | 2.11 | 306 | TransportDB |
| Bacteria | *Streptococcus* | *agalactiae* ILRI112 | 2.03 | 322 | TransportDB |
| Bacteria | *Streptococcus* | *agalactiae* NEM316 | 2.21 | 311 | TransportDB |
| Bacteria | *Streptococcus* | *agalactiae* SA2006 | 1.82 | 288 | TransportDB |
| Bacteria | *Simiduia* | *agarivorans* SA1DSM21679 | 4.3 | 279 | TransportDB |
| Bacteria | *Sphingopyxis* | *alaskensis* RB2256 | 3.37 | 216 | TransportDB |
| Bacteria | *Streptomyces* | *albus* J1074 | 6.84 | 632 | TransportDB |
| Bacteria | *Shewanella* | *amazonensis* SB2B | 4.31 | 326 | TransportDB |
| Bacteria | *Streptococcus* | *anginosus* C1051 | 1.91 | 252 | TransportDB |
| Bacteria | *Streptococcus* | *anginosus* C238 | 2.23 | 272 | TransportDB |
| Bacteria | *Spiroplasma* | *apis* B31 | 1.16 | 113 | TransportDB |
| Bacteria | *Stigmatella* | *aurantiaca* DW431 | 10.26 | 476 | TransportDB |
| Bacteria | *Staphylococcus* | *aureus* 0402981 | 2.82 | 353 | TransportDB |
| Bacteria | *Staphylococcus* | *aureus* 08BA02176 | 2.78 | 344 | TransportDB |
| Bacteria | *Staphylococcus* | *aureus aureus* MRSA USA300 FPR3757 | 2.87 | 359 | TransportDB |
| Bacteria | *Staphylococcus* | *aureus* Bmb9393 | 2.98 | 349 | TransportDB |
| Bacteria | *Staphylococcus* | *aureus* CA347 | 2.88 | 358 | TransportDB |
| Bacteria | *Staphylococcus* | *aureus* M1 | 2.89 | 353 | TransportDB |
| Bacteria | *Staphylococcus* | *aureus* RF122 | 2.74 | 347 | TransportDB |
| Bacteria | *Staphylococcus* | *aureus* subsp aureus1181997 | 2.87 | 348 | TransportDB |
| Bacteria | *Staphylococcus* | *aureus* subsp aureus552053 | 2.78 | 356 | TransportDB |
| Bacteria | *Staphylococcus* | *aureus* subsp aureus6850 | 2.74 | 344 | TransportDB |
| Bacteria | *Staphylococcus* | *aureus* subsp aureus71193 | 2.72 | 345 | TransportDB |
| Bacteria | *Staphylococcus* | *aureus* subsp aureusCN1 | 2.76 | 351 | TransportDB |
| Bacteria | *Staphylococcus* | *aureus* subsp aureusCOL | 2.81 | 347 | TransportDB |
| Bacteria | *Staphylococcus* | *aureus* subsp aureusECTR2 | 2.76 | 351 | TransportDB |
| Bacteria | *Staphylococcus* | *aureus* subsp aureusED133 | 2.83 | 347 | TransportDB |
| Bacteria | *Staphylococcus* | *aureus* subsp aureusED98 | 2.85 | 352 | TransportDB |
| Bacteria | *Staphylococcus* | *aureus subsp* aureus HO50960412 | 2.83 | 346 | TransportDB |
| Bacteria | *Staphylococcus* | *aureus* subsp aureus JH1 | 2.94 | 354 | TransportDB |
| Bacteria | *Staphylococcus* | *aureus* subsp aureus JH9 | 2.94 | 355 | TransportDB |
| Bacteria | *Staphylococcus* | *aureus* subsp aureus JKD6159 | 2.83 | 350 | TransportDB |
| Bacteria | *Staphylococcus* | *aureus* subsp aureus LGA251 | 2.75 | 347 | TransportDB |
| Bacteria | *Staphylococcus* | *aureus* subsp aureus M013 | 2.79 | 351 | TransportDB |
| Bacteria | *Staphylococcus* | *aureus* subsp aureus MRSA252 | 2.9 | 217 | TransportDB |
| Bacteria | *Staphylococcus* | *aureus* subsp aureus MSHR1132 | 2.79 | 353 | TransportDB |
| Bacteria | *Staphylococcus* | *aureus* subsp aureus MSSA476 | 2.82 | 356 | TransportDB |
| Bacteria | *Staphylococcus* | *aureus* subsp aureus Mu3 | 2.88 | 358 | TransportDB |
| Bacteria | *Staphylococcus* | *aureus* subsp aureus Mu50 | 2.9 | 359 | TransportDB |
| Bacteria | *Staphylococcus* | *aureus* subsp aureus MW2 | 2.82 | 352 | TransportDB |
| Bacteria | *Staphylococcus* | *aureus* subsp aureus N315 | 2.84 | 357 | TransportDB |
| Bacteria | *Staphylococcus* | *aureus* subsp aureus NCTC8325 | 2.82 | 350 | TransportDB |
| Bacteria | *Staphylococcus* | *aureus* subsp aureus SA40 | 2.73 | 345 | TransportDB |
| Bacteria | *Staphylococcus* | *aureus* subsp aureus SA957 | 2.79 | 347 | TransportDB |
| Bacteria | *Staphylococcus* | *aureus* subsp aureus ST228 | 2.78 | 330 | TransportDB |
| Bacteria | *Staphylococcus* | *aureus* subsp aureus ST398 | 2.89 | 350 | TransportDB |
| Bacteria | *Staphylococcus* | *aureus* subsp aureus str JKD6008 | 2.92 | 347 | TransportDB |
| Bacteria | *Staphylococcus* | *aureus* subsp aureus str Newman | 2.88 | 348 | TransportDB |
| Bacteria | *Staphylococcus* | *aureus* subsp aureus T0131 | 2.91 | 352 | TransportDB |
| Bacteria | *Staphylococcus* | *aureus* subsp aureus TCH60 | 2.83 | 356 | TransportDB |
| Bacteria | *Staphylococcus* | *aureus* subsp aureus TW20 | 3.08 | 357 | TransportDB |
| Bacteria | *Staphylococcus* | *aureus* subsp aureus USA300_FPR3757 | 2.92 | 362 | TransportDB |
| Bacteria | *Staphylococcus* | *aureus* subsp aureus USA300_TCH1516 | 2.9 | 348 | TransportDB |
| Bacteria | *Staphylococcus* | *aureus* subsp aureus VC40 | 2.69 | 350 | TransportDB |
| Bacteria | *Staphylococcus* | *aureus* subsp aureus Z172 | 3.02 | 359 | TransportDB |
| Bacteria | *Sulfurimonas* | *autotrophica* DSM16294 | 2.15 | 166 | TransportDB |
| Bacteria | *Streptomyces* | *avermitilis* MA4680 | 9.12 | 766 | TransportDB |
| Bacteria | *Sulfurihydrogenibium* | *azorense* AzFu1 | 1.64 | 112 | TransportDB |
| Bacteria | *Synergistetes* | *bacterium* SGP1 | 2.73 | 239 | TransportDB |
| Bacteria | *Shewanella* | *baltica* BA175 | 5.2 | 410 | TransportDB |
| Bacteria | *Shewanella* | *baltica* OS117 | 5.53 | 403 | TransportDB |
| Bacteria | *Shewanella* | *baltica* OS155 | 5.34 | 403 | TransportDB |
| Bacteria | *Shewanella* | *baltica* OS185 | 5.31 | 401 | TransportDB |
| Bacteria | *Shewanella* | *baltica* OS195 | 5.55 | 420 | TransportDB |
| Bacteria | *Shewanella* | *baltica* OS223 | 5.36 | 404 | TransportDB |
| Bacteria | *Shewanella* | *baltica* OS678 | 5.37 | 410 | TransportDB |
| Bacteria | *Sulfurospirillum* | *barnesii* SES3 | 2.51 | 277 | TransportDB |
| Bacteria | *Streptomyces* | *bingchenggensis* BCW1 | 11.94 | 1010 | TransportDB |
| Bacteria | *Shimwellia* | *blattae* DSM4481NBRC105725 | 4.16 | 542 | TransportDB |
| Bacteria | *Salmonella* | *bongori* N26808 | 4.77 | 596 | TransportDB |
| Bacteria | *Salmonella* | *bongori* NCTC12419 | 4.46 | 577 | TransportDB |
| Bacteria | *Shigella* | *boydii* CDC308394 | 4.87 | 498 | TransportDB |
| Bacteria | *Shigella* | *boydii* Sb227 | 4.65 | 496 | TransportDB |
| Bacteria | *Spirochaeta* | *caldaria* DSM7334 | 3.24 | 357 | TransportDB |
| Bacteria | *Solitalea* | *canadensis* DSM3403 | 5.2 | 248 | TransportDB |
| Bacteria | *Staphylococcus* | *carnosus subsp* carnosus TM300 | 2.57 | 345 | TransportDB |
| Bacteria | *Streptomyces* | *cattleya* NRRL8057DSM46488 | 8.09 | 570 | TransportDB |
| Bacteria | *Sorangium* | *cellulosum* So01572 | 14.78 | 541 | TransportDB |
| Bacteria | *Sorangium* | *cellulosum* Soce56 | 13.03 | 468 | TransportDB |
| Bacteria | *Sphingobium* | *chlorophenolicum* L1 | 4.57 | 248 | TransportDB |
| Bacteria | *Spiroplasma* | *chrysopicola* DF1 | 1.12 | 107 | TransportDB |
| Bacteria | *Sphaerochaeta* | *coccoides* DSM17374 | 2.23 | 302 | TransportDB |
| Bacteria | *Streptomyces* | *coelicolor* A32 | 9.05 | 798 | TransportDB |
| Bacteria | *Streptomyces* | *collinus* Tu365 | 8.38 | 646 | TransportDB |
| Bacteria | *Streptococcus* | *constellatus* subsp pharyngis C1050 | 1.99 | 244 | TransportDB |
| Bacteria | *Streptococcus* | *constellatus* subsp pharyngis C232 | 1.94 | 240 | TransportDB |
| Bacteria | *Streptococcus* | *constellatus* subsp pharyngis C818 | 1.94 | 239 | TransportDB |
| Bacteria | *Stanieria* | *cyanosphaera* PCC7437 | 5.54 | 340 | TransportDB |
| Bacteria | *Streptomyces* | *davawensis* JCM4913 | 9.56 | 816 | TransportDB |
| Bacteria | *Saccharophagus* | *degradans* 240 | 5.06 | 287 | TransportDB |
| Bacteria | *Sulfurospirillum* | *deleyianum* DSM6946 | 2.31 | 227 | TransportDB |
| Bacteria | *Sulfurimonas* | *denitrificans* DSM1251 | 2.2 | 169 | TransportDB |
| Bacteria | *Shewanella* | *denitrificans* OS217 | 4.55 | 295 | TransportDB |
| Bacteria | *Sulfuricella* | *denitrificans* skB26 | 3.22 | 278 | TransportDB |
| Bacteria | *Spiroplasma* | *diminutum* CUAS1 | 0.95 | 90 | TransportDB |
| Bacteria | *Shigella* | *dysenteriae* 1617 | 4.48 | 617 | TransportDB |
| Bacteria | *Shigella* | *dysenteriae* Sd197 | 4.56 | 484 | TransportDB |
| Bacteria | *Streptococcus* | *dysgalactiae* subsp equisimilis 167 | 2.08 | 326 | TransportDB |
| Bacteria | *Streptococcus* | *dysgalactiae* subsp equisimilis AC2713 | 2.18 | 307 | TransportDB |
| Bacteria | *Streptococcus* | *dysgalactiae* subsp equisimilis ATCC12394 | 2.16 | 305 | TransportDB |
| Bacteria | *Streptococcus* | *dysgalactiae* subsp equisimilis GGS_124 | 2.11 | 312 | TransportDB |
| Bacteria | *Streptococcus* | *dysgalactiae* subsp equisimilis RE378 | 2.15 | 315 | TransportDB |
| Bacteria | *Synechococcus* | *elongatus* PCC6301 | 2.7 | 225 | TransportDB |
| Bacteria | *Synechococcus* | *elongatus* PCC7942 | 2.74 | 228 | TransportDB |
| Bacteria | *Salmonella* | *enterica arizonae* sv 62:z4.z23 RSK2980 | 4.6 | 555 | TransportDB |
| Bacteria | *Salmonella* | *enterica* subsp arizonae serovar62 | 4.6 | 576 | TransportDB |
| Bacteria | *Salmonella* | *enterica* subsp enterica serovar4512 | 4.82 | 615 | TransportDB |
| Bacteria | *Salmonella* | *enterica subsp* entericaserovar Agona str 24249 | 4.76 | 595 | TransportDB |
| Bacteria | *Salmonella* | *enterica* subsp entericaserovar Agona str SL483 | 4.84 | 596 | TransportDB |
| Bacteria | *Salmonella* | *enterica* subsp entericaserovar Bareilly str CFSAN000189 | 4.81 | 625 | TransportDB |
| Bacteria | *Salmonella* | *enterica* subsp entericaserovar Bovismorbificans str 3114 | 4.77 | 613 | TransportDB |
| Bacteria | *Salmonella* | *enterica* subsp entericaserovar Choleraesuis str SCB67 | 4.94 | 594 | TransportDB |
| Bacteria | *Salmonella* | *enterica* subsp entericaserovar Cubana str CFSAN002050 | 5.27 | 631 | TransportDB |
| Bacteria | *Salmonella* | *enterica* subsp entericaserovar Dublin str CT_02021853 | 4.92 | 585 | TransportDB |
| Bacteria | *Salmonella* | *enterica* subsp entericaserovar Enteritidis str P125109 | 4.69 | 599 | TransportDB |
| Bacteria | *Salmonella* | *enterica* subsp enterica serovar Gallinarumpullorum str CDC198367 | 4.62 | 565 | TransportDB |
| Bacteria | *Salmonella* | *enterica* subsp enterica serovar Gallinarumpullorum str RKS5078 | 4.64 | 552 | TransportDB |
| Bacteria | *Salmonella* | *enterica* subsp entericaserovar Gallinarum str 28791 | 4.66 | 558 | TransportDB |
| Bacteria | *Salmonella* | *enterica* subsp enterica serovar Heidelberg str 41578 | 4.95 | 636 | TransportDB |
| Bacteria | *Salmonella* | *enterica* subsp entericab serovar Heidelberg str B182 | 4.79 | 619 | TransportDB |
| Bacteria | *Salmonella* | *enterica* subsp enterica serovar Heidelberg str CFSAN002069 | 4.93 | 632 | TransportDB |
| Bacteria | *Salmonella* | *enterica* subsp enterica serovar Heidelberg str SL476 | 4.98 | 616 | TransportDB |
| Bacteria | *Salmonella* | *enterica* subsp enterica serovar Javiana str CFSAN001992 | 4.68 | 611 | TransportDB |
| Bacteria | *Salmonella* | *enterica* subsp enterica serovar Newport str SL254 | 5.01 | 621 | TransportDB |
| Bacteria | *Salmonella* | *enterica* subsp enterica serovar Newport str USMARCS31241 | 4.92 | 624 | TransportDB |
| Bacteria | *Salmonella* | *enterica* subsp enterica serovar ParatyphiA str AKU_12601 | 4.58 | 558 | TransportDB |
| Bacteria | *Salmonella* | *enterica* subsp enterica serovar ParatyphiA str ATCC9150 | 4.59 | 560 | TransportDB |
| Bacteria | *Salmonella* | *enterica* subsp enterica serovar ParatyphiB str SPB7 | 4.86 | 627 | TransportDB |
| Bacteria | *Salmonella* | *enterica* subsp enterica serovar ParatyphiC strain RKS4594 | 4.89 | 593 | TransportDB |
| Bacteria | *Salmonella* | *enterica* subsp enterica serovar Pullorum str S06004 | 4.68 | 529 | TransportDB |
| Bacteria | *Salmonella* | *enterica* subsp enterica serovar Schwarzengrund str CVM19633 | 4.82 | 598 | TransportDB |
| Bacteria | *Salmonella* | *enterica* subsp enterica serovar Thompson str RM6836 | 4.71 | 619 | TransportDB |
| Bacteria | *Salmonella* | *enterica* subsp enterica serovar Typhimurium str 14028S | 4.96 | 620 | TransportDB |
| Bacteria | *Salmonella* | *enterica* subsp enterica serovar Typhimurium str 798 | 4.97 | 625 | TransportDB |
| Bacteria | *Salmonella* | *enterica* subsp enterica serovar Typhimurium str D23580 | 4.88 | 613 | TransportDB |
| Bacteria | *Salmonella* | *enterica* subsp enterica serovar Typhimurium str DT104 | 5.03 | 628 | TransportDB |
| Bacteria | *Salmonella* | *enterica* subsp enterica serovar Typhimurium str DT2 | 4.81 | 604 | TransportDB |
| Bacteria | *Salmonella* | *enterica* subsp enterica serovar Typhimurium str LT2 | 4.95 | 627 | TransportDB |
| Bacteria | *Salmonella* | *enterica* subsp enterica serovar Typhimurium str SL1344 | 5.07 | 631 | TransportDB |
| Bacteria | *Salmonella* | *enterica* subsp enterica serovar Typhimurium str ST474 | 5.07 | 631 | TransportDB |
| Bacteria | *Salmonella* | *enterica* subsp enterica serovar Typhimurium str T000240 | 5.07 | 651 | TransportDB |
| Bacteria | *Salmonella* | *enterica* subsp enterica serovar Typhimurium str U288 | 5.02 | 638 | TransportDB |
| Bacteria | *Salmonella* | *enterica* subsp enterica serovar Typhimurium str UK1 | 4.91 | 625 | TransportDB |
| Bacteria | *Salmonella* | *enterica* subsp enterica serovar Typhimurium var 5 str CFSAN001921 | 5.09 | 633 | TransportDB |
| Bacteria | *Salmonella* | *enterica* subsp enterica serovar Typhi str CT18 | 5.13 | 574 | TransportDB |
| Bacteria | *Salmonella* | *enterica* subsp enterica serovar Typhi str Pstx12 | 4.95 | 596 | TransportDB |
| Bacteria | *Salmonella* | *enterica* subsp enterica serovar Typhi str Ty2 | 4.79 | 568 | TransportDB |
| Bacteria | *Salmonella* | *enterica* subsp enterica serovar Typhi str Ty21a | 4.79 | 584 | TransportDB |
| Bacteria | *Salmonella* | *enterica* subsp enterica serovar Weltevreden str 20076032891 | 4.92 | 606 | TransportDB |
| Bacteria | *Salmonella* | *enterica* sv Paratyphi B SPB7 | 4.8 | 608 | TransportDB |
| Bacteria | *Staphylococcus* | *epidermidis* ATCC12228 | 2.56 | 322 | TransportDB |
| Bacteria | *Staphylococcus* | *epidermidis* RP62A | 2.64 | 311 | TransportDB |
| Bacteria | *Streptococcus* | *equi* subsp equi4047 | 2.25 | 246 | TransportDB |
| Bacteria | *Streptococcus* | *equi* subsp zooepidemicus | 2.15 | 275 | TransportDB |
| Bacteria | *Streptococcus* | *equi* subsp zooepidemicus ATCC35246 | 2.17 | 267 | TransportDB |
| Bacteria | *Streptococcus* | *equi* subsp zooepidemicus MGCS10565 | 2.02 | 272 | TransportDB |
| Bacteria | *Saccharopolyspora* | *erythraea* NRRL2338 | 8.21 | 694 | TransportDB |
| Bacteria | *Saccharothrix* | *espanaensis* DSM44229 | 9.36 | 712 | TransportDB |
| Bacteria | *Streptomyces* | *flavogriseus* ATCC33331 | 7.66 | 704 | TransportDB |
| Bacteria | *Shigella* | *flexneri* 2002017 | 4.89 | 533 | TransportDB |
| Bacteria | *Shigella* | *flexneri* 2a str 2457T | 4.6 | 514 | TransportDB |
| Bacteria | *Shigella* | *flexneri* 2a str 301 | 4.83 | 536 | TransportDB |
| Bacteria | *Shigella* | *flexneri* 5 str 8401 | 4.57 | 524 | TransportDB |
| Bacteria | *Sinorhizobium* | *fredii* HH103 | 7.81 | 896 | TransportDB |
| Bacteria | *Sinorhizobium* | *fredii* NGR234 | 6.89 | 926 | TransportDB |
| Bacteria | *Sinorhizobium* | *fredii* USDA257 | 7.03 | 935 | TransportDB |
| Bacteria | *Shewanella* | *frigidimarina* NCIMB400 | 4.85 | 376 | TransportDB |
| Bacteria | *Streptomyces* | *fulvissimus* DSM40593 | 7.91 | 729 | TransportDB |
| Bacteria | *Syntrophobacter* | *fumaroxidans* MPOB | 4.99 | 413 | TransportDB |
| Bacteria | *Streptococcus* | *gallolyticus* subsp gallolyticus ATCC43143 | 2.36 | 333 | TransportDB |
| Bacteria | *Streptococcus* | *gallolyticus* subsp gallolyticus ATCCBAA2069 | 2.38 | 349 | TransportDB |
| Bacteria | *Streptococcus* | *gallolyticus* UCN34 | 2.35 | 343 | TransportDB |
| Bacteria | *Sphaerochaeta* | *globus* str Buddy | 3.32 | 529 | TransportDB |
| Bacteria | *Sodalis* | *glossinidius* str morsitans | 4.29 | 273 | TransportDB |
| Bacteria | *Syntrophobotulus* | *glycolicus* DSM8271 | 3.41 | 374 | TransportDB |
| Bacteria | *Streptococcus* | *gordonii* str Challissub str CH1 | 2.20 | 294 | TransportDB |
| Bacteria | *Saprospira* | *grandis* str Lewin | 4.4 | 147 | TransportDB |
| Bacteria | *Streptomyces* | *griseus* subsp griseus NBRC13350 | 8.55 | 785 | TransportDB |
| Bacteria | *Staphylococcus* | *haemolyticus* JCSC1435 | 2.7 | 341 | TransportDB |
| Bacteria | *Shewanella* | *halifaxensis* HAWEB4 | 5.23 | 436 | TransportDB |
| Bacteria | *Slackia* | *heliotrinireducens* DSM20476 | 3.17 | 231 | TransportDB |
| Bacteria | *Streptomyces* | *hygroscopicus* subsp jinggangensis 5008 | 10.38 | 783 | TransportDB |
| Bacteria | *Streptomyces* | *hygroscopicus* subsp jinggangensis TL01 | 10.08 | 768 | TransportDB |
| Bacteria | *Streptococcus* | *infantarius* subsp infantarius CJ18 | 2.01 | 266 | TransportDB |
| Bacteria | *Streptococcus* | *iniae* SF1 | 2.15 | 298 | TransportDB |
| Bacteria | *Streptococcus* | *intermedius* B196 | 2.0 | 267 | TransportDB |
| Bacteria | *Streptococcus* | *intermedius* C270 | 1.96 | 258 | TransportDB |
| Bacteria | *Streptococcus* | *intermedius* JTH08 | 1.93 | 270 | TransportDB |
| Bacteria | *Sanguibacter* | *keddieii* DSM10542 | 4.25 | 549 | TransportDB |
| Bacteria | *Sulfuricurvum* | *kujiense* DSM16994 | 2.82 | 192 | TransportDB |
| Bacteria | *Strawberry* | *lethal yellows phytoplasma* CPA str NZSb11 | 0.96 | 52 | TransportDB |
| Bacteria | *Spirosoma* | *linguale* DSM74 | 8.49 | 394 | TransportDB |
| Bacteria | *Syntrophothermus* | *lipocalidus* DSM12680 | 2.41 | 178 | TransportDB |
| Bacteria | *Serratia* | *liquefaciens* ATCC27592 | 5.28 | 787 | TransportDB |
| Bacteria | *Sideroxydans* | *lithotrophicus* ES1 | 3.0 | 234 | TransportDB |
| Bacteria | *Shewanella* | *loihica* PV4 | 4.6 | 347 | TransportDB |
| Bacteria | *Staphylococcus* | *lugdunensis* HKU0901 | 2.66 | 380 | TransportDB |
| Bacteria | *Staphylococcus* | *lugdunensis* N920143 | 2.6 | 354 | TransportDB |
| Bacteria | *Streptococcus* | *lutetiensis* 033 | 1.98 | 248 | TransportDB |
| Bacteria | *Streptococcus* | *macedonicus* ACADC198 | 2.14 | 295 | TransportDB |
| Bacteria | *Stenotrophomonas* | *maltophilia* D457 | 4.77 | 357 | TransportDB |
| Bacteria | *Stenotrophomonas* | *maltophilia* JV3 | 4.54 | 350 | TransportDB |
| Bacteria | *Stenotrophomonas* | *maltophilia* K279a | 4.85 | 358 | TransportDB |
| Bacteria | *Stenotrophomonas* | *maltophilia* R5513 | 4.57 | 351 | TransportDB |
| Bacteria | *Serratia* | *marcescens* FGI94 | 4.86 | 638 | TransportDB |
| Bacteria | *Serratia* | *marcescens* WW4 | 5.24 | 748 | TransportDB |
| Bacteria | *Sinorhizobium* | *medicae* WSM419 | 6.82 | 1013 | TransportDB |
| Bacteria | *Sinorhizobium* | *meliloti* 1021 | 6.69 | 1024 | TransportDB |
| Bacteria | *Sinorhizobium* | *meliloti* 2011 | 6.69 | 1023 | TransportDB |
| Bacteria | *Sinorhizobium* | *meliloti* AK83 | 7.14 | 971 | TransportDB |
| Bacteria | *Sinorhizobium* | *meliloti* BL225C | 6.98 | 1023 | TransportDB |
| Bacteria | *Sinorhizobium* | *meliloti* GR4 | 7.14 | 1027 | TransportDB |
| Bacteria | *Sinorhizobium* | *meliloti* Rm41 | 7.15 | 1105 | TransportDB |
| Bacteria | *Sinorhizobium* | *meliloti* SM11 | 7.17 | 1024 | TransportDB |
| Bacteria | *Streptococcus* | *mitis* B6 | 2.15 | 269 | TransportDB |
| Bacteria | *Streptobacillus* | *moniliformis* DSM12112 | 1.67 | 229 | TransportDB |
| Bacteria | *Streptococcus* | *mutans* GS5 | 2.03 | 268 | TransportDB |
| Bacteria | *Streptococcus* | *mutans* LJ23 | 2.02 | 269 | TransportDB |
| Bacteria | *Streptococcus* | *mutans* NN2025 | 2.01 | 280 | TransportDB |
| Bacteria | *Streptococcus* | *mutans* UA159 | 2.03 | 279 | TransportDB |
| Bacteria | *Stackebrandtia* | *nassauensis* DSM44728 | 6.84 | 671 | TransportDB |
| Bacteria | *Simkania* | *negevensis* Z | 2.63 | 204 | TransportDB |
| Bacteria | *Starkeya* | *novella* DSM506 | 4.77 | 701 | TransportDB |
| Bacteria | *Streptococcus* | *oligofermentans* AS13089 | 2.14 | 235 | TransportDB |
| Bacteria | *Shewanella* | *oneidensis* MR1 | 5.13 | 366 | TransportDB |
| Bacteria | *Streptococcus* | *oralis* Uo5 | 1.96 | 239 | TransportDB |
| Bacteria | *Streptococcus* | *parasanguinis* ATCC15912 | 2.15 | 294 | TransportDB |
| Bacteria | *Streptococcus* | *parasanguinis* FW213 | 2.17 | 292 | TransportDB |
| Bacteria | *Streptococcus* | *parauberis* KCTC11537 | 2.14 | 304 | TransportDB |
| Bacteria | *Staphylococcus* | *pasteuri* SP1 | 2.56 | 329 | TransportDB |
| Bacteria | *Streptococcus* | *pasteurianus* ATCC43144 | 2.1 | 308 | TransportDB |
| Bacteria | *Shewanella* | *pealeana* ATCC700345 | 5.17 | 427 | TransportDB |
| Bacteria | *Shewanella* | *piezotolerans* WP3 | 5.4 | 394 | TransportDB |
| Bacteria | *Sphaerochaeta* | *pleomorpha* str Grapes | 3.59 | 636 | TransportDB |
| Bacteria | *Serratia* | *plymuthica* 4Rx13 | 5.4 | 794 | TransportDB |
| Bacteria | *Serratia* | *plymuthica* AS9 | 5.44 | 783 | TransportDB |
| Bacteria | *Serratia* | *plymuthica* S13 | 5.47 | 804 | TransportDB |
| Bacteria | *Streptococcus* | *pneumoniae* 6706B | 2.24 | 329 | TransportDB |
| Bacteria | *Streptococcus* | *pneumoniae* 70585 | 2.18 | 314 | TransportDB |
| Bacteria | *Streptococcus* | *pneumoniae* A026 | 2.09 | 302 | TransportDB |
| Bacteria | *Streptococcus* | *pneumoniae* AP200 | 2.13 | 321 | TransportDB |
| Bacteria | *Streptococcus* | *pneumoniae* ATCC700669 | 2.22 | 315 | TransportDB |
| Bacteria | *Streptococcus* | *pneumoniae* CGSP14 | 2.21 | 329 | TransportDB |
| Bacteria | *Streptococcus* | *pneumoniae* D39 | 2.05 | 304 | TransportDB |
| Bacteria | *Streptococcus* | *pneumoniae* G54 | 2.08 | 322 | TransportDB |
| Bacteria | *Streptococcus* | *pneumoniae* gamPNI0373 | 2.06 | 318 | TransportDB |
| Bacteria | *Streptococcus* | *pneumoniae* Hungary19A6 | 2.25 | 306 | TransportDB |
| Bacteria | *Streptococcus* | *pneumoniae* INV104 | 2.14 | 304 | TransportDB |
| Bacteria | *Streptococcus* | *pneumoniae* INV200 | 2.09 | 311 | TransportDB |
| Bacteria | *Streptococcus* | *pneumoniae* JJA | 2.12 | 300 | TransportDB |
| Bacteria | *Streptococcus* | *pneumoniae* OXC141 | 2.04 | 296 | TransportDB |
| Bacteria | *Streptococcus* | *pneumoniae* P1031 | 2.11 | 298 | TransportDB |
| Bacteria | *Streptococcus* | *pneumoniae* R6 | 2.04 | 316 | TransportDB |
| Bacteria | *Streptococcus* | *pneumoniae* SPN034156 | 2.02 | 295 | TransportDB |
| Bacteria | *Streptococcus* | *pneumoniae* SPN034183 | 2.04 | 291 | TransportDB |
| Bacteria | *Streptococcus* | *pneumoniae* SPN994038 | 2.03 | 292 | TransportDB |
| Bacteria | *Streptococcus* | *pneumoniae* SPN994039 | 2.03 | 292 | TransportDB |
| Bacteria | *Streptococcus* | *pneumoniae* SPNA45 | 2.13 | 276 | TransportDB |
| Bacteria | *Streptococcus* | *pneumoniae* ST556 | 2.15 | 313 | TransportDB |
| Bacteria | *Streptococcus* | *pneumoniae* Taiwan19F14 | 2.11 | 298 | TransportDB |
| Bacteria | *Streptococcus* | *pneumoniae* TCH843119A | 2.09 | 314 | TransportDB |
| Bacteria | *Streptococcus* | *pneumoniae* TIGR4 | 2.16 | 309 | TransportDB |
| Bacteria | *Silicibacter* | *pomeroyi* DSS*-3* | 4.11 | 620 | TransportDB |
| Bacteria | *Serratia* | *proteamaculans* 568 | 5.5 | 794 | TransportDB |
| Bacteria | *Staphylococcus* | *pseudintermedius* ED99 | 2.57 | 331 | TransportDB |
| Bacteria | *Staphylococcus* | *pseudintermedius* HKU1003 | 2.62 | 337 | TransportDB |
| Bacteria | *Streptococcus* | *pseudopneumoniae* IS7493 | 2.2 | 289 | TransportDB |
| Bacteria | *Shewanella* | *putrefaciens* 200 | 4.84 | 371 | TransportDB |
| Bacteria | *Shewanella* | *putrefaciens* CN32 | 4.66 | 395 | TransportDB |
| Bacteria | *Streptococcus* | *pyogenes* A20 | 1.84 | 238 | TransportDB |
| Bacteria | *Streptococcus* | *pyogenes* Alab49 | 1.83 | 236 | TransportDB |
| Bacteria | *Streptococcus* | *pyogenes* HSC5 | 1.82 | 235 | TransportDB |
| Bacteria | *Streptococcus* | *pyogenes* M1476 | 1.83 | 250 | TransportDB |
| Bacteria | *Streptococcus* | *pyogenes* M1GAS | 1.85 | 235 | TransportDB |
| Bacteria | *Streptococcus* | *pyogenes* MGAS10270 | 1.93 | 245 | TransportDB |
| Bacteria | *Streptococcus* | *pyogenes* MGAS10394 | 1.9 | 235 | TransportDB |
| Bacteria | *Streptococcus* | *pyogenes* MGAS10750 | 1.94 | 244 | TransportDB |
| Bacteria | *Streptococcus* | *pyogenes* MGAS15252 | 1.75 | 233 | TransportDB |
| Bacteria | *Streptococcus* | *pyogenes* MGAS1882 | 1.78 | 233 | TransportDB |
| Bacteria | *Streptococcus* | *pyogenes* MGAS2096 | 1.86 | 252 | TransportDB |
| Bacteria | *Streptococcus* | *pyogenes* MGAS315 | 1.9 | 236 | TransportDB |
| Bacteria | *Streptococcus* | *pyogenes* MGAS5005 | 1.84 | 243 | TransportDB |
| Bacteria | *Streptococcus* | *pyogenes* MGAS6180 | 1.9 | 241 | TransportDB |
| Bacteria | *Streptococcus* | *pyogenes* MGAS8232 | 1.9 | 232 | TransportDB |
| Bacteria | *Streptococcus* | *pyogenes* MGAS9429 | 1.84 | 237 | TransportDB |
| Bacteria | *Streptococcus* | *pyogenes* NZ131 | 1.82 | 233 | TransportDB |
| Bacteria | *Streptococcus* | *pyogenes* SSI1 | 1.89 | 232 | TransportDB |
| Bacteria | *Streptococcus* | *pyogenes* str Manfredo | 1.84 | 232 | TransportDB |
| Bacteria | *Streptomyces* | *rapamycinicus* NRRL5491 | 12.7 | 986 | TransportDB |
| Bacteria | *Streptosporangium* | *roseum* DSM43021 | 10.37 | 891 | TransportDB |
| Bacteria | *Segniliparus* | *rotundus* DSM44985 | 3.16 | 220 | TransportDB |
| Bacteria | *Salinibacter* | *ruber* DSM13855 | 3.59 | 223 | TransportDB |
| Bacteria | *Salinibacter* | *ruber* M8 | 3.83 | 97 | TransportDB |
| Bacteria | *Selenomonas* | *ruminantium* subsp lactilytica TAM6421 | 3.63 | 321 | TransportDB |
| Bacteria | *Spiribacter* | *salinus* M1940 | 1.74 | 217 | TransportDB |
| Bacteria | *Streptococcus* | *salivarius* CCHSS3 | 2.22 | 294 | TransportDB |
| Bacteria | *Streptococcus* | *salivarius* JIM8777 | 2.21 | 276 | TransportDB |
| Bacteria | *Streptococcus* | *sanguinis* SK36 | 2.39 | 303 | TransportDB |
| Bacteria | *Staphylococcus* | *saprophyticus* subsp saprophyticus ATCC15305 | 2.58 | 381 | TransportDB |
| Bacteria | *Streptomyces* | *scabiei* 8722 | 10.15 | 775 | TransportDB |
| Bacteria | *Shewanella* | *sediminis* HAWEB3 | 5.52 | 458 | TransportDB |
| Bacteria | *Solibacillus* | *silvestris* StLB046 | 3.98 | 471 | TransportDB |
| Bacteria | *Spirochaeta* | *smaragdinae* DSM11293 | 4.65 | 747 | TransportDB |
| Bacteria | *Shigella* | *sonnei* 53G | 5.22 | 601 | TransportDB |
| Bacteria | *Shigella* | *sonnei* Ss046 | 5.06 | 540 | TransportDB |
| Bacteria | *Sphingobacterium* | *s21* | 6.23 | 354 | TransportDB |
| Bacteria | *Shewanella* | *sANA3* | 5.25 | 396 | TransportDB |
| Bacteria | *Serratia* | *sAS12* | 5.44 | 783 | TransportDB |
| Bacteria | *Serratia* | *sAS13* | 5.44 | 782 | TransportDB |
| Bacteria | *Serratia* | *sATCC39006* | 4.95 | 730 | TransportDB |
| Bacteria | *Synechococcus* | *sCC9311* | 2.61 | 199 | TransportDB |
| Bacteria | *Synechococcus* | *sCC9605* | 2.51 | 169 | TransportDB |
| Bacteria | *Synechococcus* | *sCC9902* | 2.23 | 152 | TransportDB |
| Bacteria | *Streptococcus* | *sIG2* | 1.99 | 256 | TransportDB |
| Bacteria | *Streptococcus* | *sIP16* | 2.02 | 272 | TransportDB |
| Bacteria | *Synechococcus* | *sJA23Ba213* | 3.05 | 245 | TransportDB |
| Bacteria | *Synechococcus* | *sJA33Ab* | 2.93 | 223 | TransportDB |
| Bacteria | *Spirochaeta* | *sL21RPulD2* | 3.78 | 370 | TransportDB |
| Bacteria | *Sphingomonas* | *sMM1* | 4.63 | 315 | TransportDB |
| Bacteria | *Shewanella* | *sMR4* | 4.71 | 371 | TransportDB |
| Bacteria | *Shewanella* | *sMR7* | 4.8 | 370 | TransportDB |
| Bacteria | *Sulfurovum* | *sNBC371* | 2.56 | 182 | TransportDB |
| Bacteria | *Streptomyces* | *sPAMC26508* | 7.63 | 706 | TransportDB |
| Bacteria | *Synechococcus* | *sPCC6312* | 3.72 | 278 | TransportDB |
| Bacteria | *Synechocystis* | *sPCC6803* | 3.95 | 297 | TransportDB |
| Bacteria | *Synechocystis* | *sPCC6803* sub str GTI | 3.57 | 290 | TransportDB |
| Bacteria | *Synechocystis* | *sPCC6803* sub str PCCN | 3.57 | 290 | TransportDB |
| Bacteria | *Synechocystis* | *sPCC6803* sub str PCCP | 3.57 | 290 | TransportDB |
| Bacteria | *Synechococcus* | *sPCC7002* | 3.41 | 275 | TransportDB |
| Bacteria | *Synechococcus* | *sPCC7502* | 3.58 | 256 | TransportDB |
| Bacteria | *Synechococcus* | *sRCC307* | 2.22 | 171 | TransportDB |
| Bacteria | *Streptomyces* | *sSirexAAE* | 7.41 | 707 | TransportDB |
| Bacteria | *Sphingobium* | *sSYK6* | 4.35 | 295 | TransportDB |
| Bacteria | *Silicibacter* | *sTM1040* | 3.2 | 501 | TransportDB |
| Bacteria | *Spiribacter* | *sUAHSP71* | 1.93 | 250 | TransportDB |
| Bacteria | *Shewanella* | *sW3181* | 4.71 | 384 | TransportDB |
| Bacteria | *Synechococcus* | *sWH7803* | 2.37 | 204 | TransportDB |
| Bacteria | *Synechococcus* | *sWH8102* | 2.43 | 172 | TransportDB |
| Bacteria | *Sulfurihydrogenibium* | *sYO3AOP1* | 1.84 | 120 | TransportDB |
| Bacteria | *Selenomonas* | *sputigena* ATCC35185 | 2.57 | 243 | TransportDB |
| Bacteria | *Streptococcus* | *suis* 05ZYH33 | 2.1 | 294 | TransportDB |
| Bacteria | *Streptococcus* | *suis* 98HAH33 | 2.1 | 279 | TransportDB |
| Bacteria | *Streptococcus* | *suis* A7 | 2.04 | 261 | TransportDB |
| Bacteria | *Streptococcus* | *suis* BM407 | 2.17 | 262 | TransportDB |
| Bacteria | *Streptococcus* | *suis* D12 | 2.18 | 260 | TransportDB |
| Bacteria | *Streptococcus* | *suis* D9 | 2.18 | 266 | TransportDB |
| Bacteria | *Streptococcus* | *suis* GZ1 | 2.04 | 261 | TransportDB |
| Bacteria | *Streptococcus* | *suis* JS14 | 2.14 | 267 | TransportDB |
| Bacteria | *Streptococcus* | *suis* P17 | 2.01 | 253 | TransportDB |
| Bacteria | *Streptococcus* | *suis* S735 | 1.98 | 258 | TransportDB |
| Bacteria | *Streptococcus* | *suis* SC070731 | 2.14 | 270 | TransportDB |
| Bacteria | *Streptococcus* | *suis* SC84 | 2.1 | 260 | TransportDB |
| Bacteria | *Streptococcus* | *suis* SS12 | 2.1 | 261 | TransportDB |
| Bacteria | *Streptococcus* | *suis* ST1 | 2.03 | 241 | TransportDB |
| Bacteria | *Streptococcus* | *suis* ST3 | 2.03 | 256 | TransportDB |
| Bacteria | *Streptococcus* | *suis* T15 | 2.24 | 266 | TransportDB |
| Bacteria | *Streptococcus* | *suis* TL13 | 2.04 | 245 | TransportDB |
| Bacteria | *Streptococcus* | *suis* YB51 | 2.04 | 265 | TransportDB |
| Bacteria | *Serratia* | *symbiotica* str Cinaracedri | 1.76 | 74 | TransportDB |
| Bacteria | *Spiroplasma* | *syrphidicola* EA1 | 1.11 | 114 | TransportDB |
| Bacteria | *Spiroplasma* | *taiwanense* CT1 | 1.09 | 94 | TransportDB |
| Bacteria | *Sebaldella* | *termitidis* ATCC33386 | 4.49 | 501 | TransportDB |
| Bacteria | *Spirochaeta* | *thermophila* DSM6192 | 2.47 | 261 | TransportDB |
| Bacteria | *Spirochaeta* | *thermophila* DSM6578 | 2.56 | 274 | TransportDB |
| Bacteria | *Symbiobacterium* | *thermophilum* IAM14863 | 3.57 | 423 | TransportDB |
| Bacteria | *Streptococcus* | *thermophilus* CNRZ1066 | 1.8 | 269 | TransportDB |
| Bacteria | *Sphaerobacter* | *thermophilus* DSM20745 | 3.99 | 405 | TransportDB |
| Bacteria | *Streptococcus* | *thermophilus* JIM8232 | 1.93 | 264 | TransportDB |
| Bacteria | *Streptococcus* | *thermophilus* LMD9 | 1.86 | 217 | TransportDB |
| Bacteria | *Streptococcus* | *thermophilus* LMG18311 | 1.8 | 266 | TransportDB |
| Bacteria | *Streptococcus* | *thermophilus* MNZLW002 | 1.85 | 248 | TransportDB |
| Bacteria | *Streptococcus* | *thermophilus* ND03 | 1.83 | 245 | TransportDB |
| Bacteria | *Salinispora* | *tropica* CNB440 | 5.18 | 413 | TransportDB |
| Bacteria | *Streptococcus* | *uberis* 0140J | 1.85 | 270 | TransportDB |
| Bacteria | *Solibacter* | *usitatus* Ellin6076 | 9.97 | 495 | TransportDB |
| Bacteria | *Streptomyces* | *venezuelae* ATCC10712 | 8.23 | 761 | TransportDB |
| Bacteria | *Shewanella* | *violacea* DSS12 | 4.96 | 361 | TransportDB |
| Bacteria | *Streptomyces* | *violaceusniger* Tu4113 | 11.14 | 815 | TransportDB |
| Bacteria | *Saccharomonospora* | *viridis* DSM43017 | 4.31 | 369 | TransportDB |
| Bacteria | *Staphylococcus* | *warneri* SG1 | 2.56 | 332 | TransportDB |
| Bacteria | *Sphingomonas* | *wittichii* RW1 | 5.92 | 325 | TransportDB |
| Bacteria | *Syntrophomonas* | *wolfei* subsp wolfeistr Goettingen | 2.94 | 208 | TransportDB |
| Bacteria | *Shewanella* | *woodyi* ATCC51908 | 5.94 | 430 | TransportDB |
| Bacteria | *Tepidanaerobacter* | *acetatoxydans* Re1 | 2.76 | 347 | TransportDB |
| Bacteria | *Thermanaerovibrio* | *acidaminovorans* DSM6589 | 1.85 | 200 | TransportDB |
| Bacteria | *Thermosipho* | *africanus* TCF52B | 2.02 | 235 | TransportDB |
| Bacteria | *Thermocrinis* | *albus* DSM14484 | 1.5 | 115 | TransportDB |
| Bacteria | *Thermovibrio* | *ammonificans* HB1 | 1.76 | 121 | TransportDB |
| Bacteria | *Taylorella* | *asinigenitalis* 1445 | 1.54 | 178 | TransportDB |
| Bacteria | *Taylorella* | *asinigenitalis* MCE3 | 1.64 | 184 | TransportDB |
| Bacteria | *Tolumonas* | *auensis* DSM9187 | 3.47 | 428 | TransportDB |
| Bacteria | *Treponema* | *azotonutricium* ZAS9 | 3.86 | 411 | TransportDB |
| Bacteria | *Thermobispora* | *bispora* DSM43833 | 4.19 | 368 | TransportDB |
| Bacteria | *Treponema* | *brennaborense* DSM12168 | 3.06 | 328 | TransportDB |
| Bacteria | *Thermoanaerobacter* | *brockii* subsp finnii Ako1 | 2.34 | 259 | TransportDB |
| Bacteria | *Thermobacillus* | *composti* KWC4 | 4.36 | 544 | TransportDB |
| Bacteria | *Thiomicrospira* | *crunogena* XCL2 | 2.43 | 225 | TransportDB |
| Bacteria | *Thermomonospora* | *curvata* DSM43183 | 5.64 | 384 | TransportDB |
| Bacteria | *Thioalkalimicrobium* | *cyclicum* ALM1 | 1.93 | 140 | TransportDB |
| Bacteria | *Thiobacillus* | *denitrificans* ATCC25259 | 2.91 | 233 | TransportDB |
| Bacteria | *Treponema* | *denticola* ATCC35405 | 2.84 | 336 | TransportDB |
| Bacteria | *Thermosynechococcus* | *elongatus* BP1 | 2.59 | 215 | TransportDB |
| Bacteria | *Taylorella* | *equigenitalis* 1456 | 1.65 | 194 | TransportDB |
| Bacteria | *Taylorella* | *equigenitalis* ATCC35865 | 1.73 | 197 | TransportDB |
| Bacteria | *Taylorella* | *equigenitalis* MCE9 | 1.7 | 197 | TransportDB |
| Bacteria | *Trichodesmium* | *erythraeum* IMS101 | 7.75 | 252 | TransportDB |
| Bacteria | *Thermoanaerobacter* | *ethanolicus* X514 | 2.46 | 289 | TransportDB |
| Bacteria | *Tannerella* | *forsythia* ATCC43037 | 3.41 | 157 | TransportDB |
| Bacteria | *Thermobifida* | *fusca* YX | 3.64 | 272 | TransportDB |
| Bacteria | *Thermodesulfobacterium* | *geofontis* OPF15 | 1.63 | 123 | TransportDB |
| Bacteria | *Tetragenococcus* | *halophilus* NBRC12172 | 2.56 | 378 | TransportDB |
| Bacteria | *Thermodesulfatator* | *indicus* DSM15286 | 2.32 | 208 | TransportDB |
| Bacteria | *Thiomonas* | *intermedia* K12 | 3.46 | 360 | TransportDB |
| Bacteria | *Thermoanaerobacter* | *italicus* Ab9 | 2.45 | 275 | TransportDB |
| Bacteria | *Thermotoga* | *lettingae* TMO | 2.14 | 396 | TransportDB |
| Bacteria | *Thermovirga* | *lienii* DSM17291 | 2.0 | 257 | TransportDB |
| Bacteria | *Thermaerobacter* | *marianensis* DSM12885 | 2.84 | 298 | TransportDB |
| Bacteria | *Thermotoga* | *maritima* MSB8 | 1.86 | 268 | TransportDB |
| Bacteria | *Thermoanaerobacter* | *mathranii* subsp mathranii str A3 | 2.31 | 272 | TransportDB |
| Bacteria | *Thermosipho* | *melanesiensis* BI429 | 1.92 | 207 | TransportDB |
| Bacteria | *Thioflavicoccus* | *mobilis* 8321 | 4.14 | 296 | TransportDB |
| Bacteria | *Tistrella* | *mobilis* KA081020065 | 6.51 | 973 | TransportDB |
| Bacteria | *Thermotoga* | *naphthophila* RKU10 | 1.81 | 256 | TransportDB |
| Bacteria | *Thermodesulfobium* | *narugense* DSM14796 | 1.9 | 165 | TransportDB |
| Bacteria | *Thermotoga* | *neapolitana* DSM4359 | 1.88 | 308 | TransportDB |
| Bacteria | *Thioalkalivibrio* | *nitratireducens* DSM14787 | 4.0 | 267 | TransportDB |
| Bacteria | *Thermosediminibacter* | *oceani* DSM16646 | 2.28 | 264 | TransportDB |
| Bacteria | *Thalassolituus* | *oleivorans* MIL1 | 3.92 | 271 | TransportDB |
| Bacteria | *Thermus* | *oshimai* JL2 | 2.4 | 279 | TransportDB |
| Bacteria | *Treponema* | *pallidum* str FribourgBlanc | 1.14 | 106 | TransportDB |
| Bacteria | *Treponema* | *pallidum* subsp pallidum DAL1 | 1.14 | 107 | TransportDB |
| Bacteria | *Treponema* | *pallidum* subsp pallidumSS14 | 2.28 | 212 | TransportDB |
| Bacteria | *Treponema* | *pallidum* subsp pallidum str Chicago | 1.14 | 100 | TransportDB |
| Bacteria | *Treponema* | *pallidum* subsp pallidum str MexicoA | 1.14 | 107 | TransportDB |
| Bacteria | *Treponema* | *pallidum* subsp pallidum str Nichols | 1.14 | 105 | TransportDB |
| Bacteria | *Treponema* | *pallidum* subsp pertenue str CDC2 | 1.14 | 106 | TransportDB |
| Bacteria | *Treponema* | *pallidum* subsp pertenue str Gauthier | 1.14 | 106 | TransportDB |
| Bacteria | *Treponema* | *pallidum* subsp pertenue str SamoaD | 1.14 | 106 | TransportDB |
| Bacteria | *Treponema* | *paraluiscuniculi cuniculi* A | 1.13 | 109 | TransportDB |
| Bacteria | *Turneriella* | *parva* DSM21527 | 4.41 | 225 | TransportDB |
| Bacteria | *Tsukamurella* | *paurometabola* DSM20162 | 4.48 | 440 | TransportDB |
| Bacteria | *Treponema* | *pedis* str TA4 | 2.89 | 352 | TransportDB |
| Bacteria | *Thermotoga* | *petrophila* RKU1 | 1.82 | 252 | TransportDB |
| Bacteria | *Thermacetogenium* | *phaeum* DSM12270 | 2.94 | 247 | TransportDB |
| Bacteria | *Thermincola* | *potens* JR | 3.16 | 273 | TransportDB |
| Bacteria | *Treponema* | *primitia* ZAS2 | 4.06 | 468 | TransportDB |
| Bacteria | *Thermoanaerobacter* | *pseudethanolicus* ATCC33223 | 2.36 | 262 | TransportDB |
| Bacteria | *Truepera* | *radiovictrix* DSM17093 | 3.26 | 416 | TransportDB |
| Bacteria | *Thermomicrobium* | *roseum* DSM5159 | 2.92 | 284 | TransportDB |
| Bacteria | *Terriglobus* | *roseus* DSM18391 | 5.23 | 294 | TransportDB |
| Bacteria | *Thermus* | *scotoductus* SA01 | 2.36 | 282 | TransportDB |
| Bacteria | *Thiomonas* | *s3As* | 3.79 | 371 | TransportDB |
| Bacteria | *Thermus* | *sCCB_US3_UF1* | 2.26 | 279 | TransportDB |
| Bacteria | *Thioalkalivibrio* | *sK90mix* | 2.99 | 222 | TransportDB |
| Bacteria | *Thauera* | *sMZ1T* | 4.57 | 458 | TransportDB |
| Bacteria | *Thermosynechococcus* | *sNK55a* | 2.52 | 199 | TransportDB |
| Bacteria | *Thermotoga* | *sRQ2* | 1.88 | 280 | TransportDB |
| Bacteria | *Thermoanaerobacter* | *sX513* | 2.46 | 291 | TransportDB |
| Bacteria | *Thermoanaerobacter* | *sX514* | 2.46 | 289 | TransportDB |
| Bacteria | *Treponema* | *succinifaciens* DSM2489 | 2.9 | 208 | TransportDB |
| Bacteria | *Thioalkalivibrio* | *sulfidophilus* HLEbGr7 | 3.46 | 267 | TransportDB |
| Bacteria | *Terriglobus* | *saanensis* SP1PR4 | 5.1 | 290 | TransportDB |
| Bacteria | *Thermoanaerobacter* | *tengcongensis* MB4 | 2.69 | 298 | TransportDB |
| Bacteria | *Thermobaculum* | *terrenum* ATCCBAA798 | 3.1 | 375 | TransportDB |
| Bacteria | *Thermotoga* | *thermarum* DSM5069 | 2.04 | 288 | TransportDB |
| Bacteria | *Thermus* | *thermophilus* HB27 | 2.13 | 236 | TransportDB |
| Bacteria | *Thermus* | *thermophilus* HB8 | 2.12 | 229 | TransportDB |
| Bacteria | *Thermus* | *thermophilus* JL18 | 2.31 | 278 | TransportDB |
| Bacteria | *Thermus* | *thermophilus* SG05JP1716 | 2.3 | 264 | TransportDB |
| Bacteria | *Thermoanaerobacterium* | *thermosaccharolyticum* DSM571 | 2.79 | 300 | TransportDB |
| Bacteria | *Thermoanaerobacterium* | *thermosaccharolyticum* M0795 | 2.89 | 325 | TransportDB |
| Bacteria | *Teredinibacter* | *turnerae* T7901 | 5.19 | 286 | TransportDB |
| Bacteria | *Thiocystis* | *violascens* DSM198 | 5.02 | 313 | TransportDB |
| Bacteria | *Tropheryma* | *whipplei* str Twist | 0.93 | 78 | TransportDB |
| Bacteria | *Tropheryma* | *whipplei* TW0827 | 0.93 | 78 | TransportDB |
| Bacteria | *Thermoanaerobacter* | *wiegelii* Rt8B1 | 2.79 | 308 | TransportDB |
| Bacteria | *Thermoanaerobacterium* | *xylanolyticum* LX11 | 2.53 | 272 | TransportDB |
| Bacteria | *Thermodesulfovibrio* | *yellowstonii* DSM11347 | 2.0 | 166 | TransportDB |
| Bacteria | *Ureaplasma* | *parvum* serovar3 str ATCC27815 | 0.75 | 68 | TransportDB |
| Bacteria | *Ureaplasma* | *parvum* serovar3 str ATCC700970 | 0.75 | 68 | TransportDB |
| Bacteria | *Ureaplasma* | *urealyticum* (parvum) sv 3 ATCC 700970 | 0.75 | 84 | TransportDB |
| Bacteria | *Ureaplasma* | *urealyticum* serovar10 str ATCC33699 | 0.87 | 66 | TransportDB |
| Bacteria | *Vibrio* | *alginolyticus* NBRC15630 ATCC17749 | 5.15 | 590 | TransportDB |
| Bacteria | *Vibrio* | *anguillarum* 775 | 4.05 | 434 | TransportDB |
| Bacteria | *Vibrio* | *cholerae* IEC224 | 4.08 | 458 | TransportDB |
| Bacteria | *Vibrio* | *cholerae* LMA39844 | 3.74 | 431 | TransportDB |
| Bacteria | *Vibrio* | *cholerae* M662 | 3.94 | 460 | TransportDB |
| Bacteria | *Vibrio* | *cholerae* MJ1236 | 4.24 | 465 | TransportDB |
| Bacteria | *Vibrio* | *cholerae* O1 biovar El Tor str N16961 | 4.03 | 456 | TransportDB |
| Bacteria | *Vibrio* | *cholerae O1* str 2010EL1786 | 4.08 | 467 | TransportDB |
| Bacteria | *Vibrio* | *cholerae* O395 | 4.13 | 466 | TransportDB |
| Bacteria | *Verminephrobacter* | *eiseniae* EF012 | 5.6 | 1015 | TransportDB |
| Bacteria | *Vibrio* | *fischeri* ES114 | 4.27 | 482 | TransportDB |
| Bacteria | *Vibrio* | *fischeri* MJ11 | 4.5 | 482 | TransportDB |
| Bacteria | *Vibrio* | *furnissii* NCTC11218 | 4.92 | 629 | TransportDB |
| Bacteria | *Vibrio* | *harveyi* ATCCBAA1116 | 6.06 | 574 | TransportDB |
| Bacteria | *Verrucosispora* | *maris* AB18032 | 6.73 | 573 | TransportDB |
| Bacteria | *Vibrio* | *nigripulchritudo* | 6.32 | 859 | TransportDB |
| Bacteria | *Variovorax* | *paradoxus* B4 | 7.15 | 1057 | TransportDB |
| Bacteria | *Variovorax* | *paradoxus* EPS | 6.55 | 868 | TransportDB |
| Bacteria | *Variovorax* | *paradoxus* S110 | 6.76 | 949 | TransportDB |
| Bacteria | *Vibrio* | *parahaemolyticus* BB22OP | 5.1 | 545 | TransportDB |
| Bacteria | *Vibrio* | *parahaemolyticus* RIMD2210633 | 5.17 | 566 | TransportDB |
| Bacteria | *Veillonella* | *parvula* DSM2008 | 2.13 | 246 | TransportDB |
| Bacteria | *Vibrio* | *sEJY3* | 5.45 | 667 | TransportDB |
| Bacteria | *Vibrio* | *sEx25* | 5.09 | 579 | TransportDB |
| Bacteria | *Vibrio* | *splendidus* LGP32 | 4.97 | 560 | TransportDB |
| Bacteria | *Vibrio* | *vulnificus* CMCP6 | 5.13 | 548 | TransportDB |
| Bacteria | *Vibrio* | *vulnificus* MO624O | 5.01 | 538 | TransportDB |
| Bacteria | *Vibrio* | *vulnificus* YJ016 | 5.26 | 560 | TransportDB |
| Bacteria | *Waddlia* | *chondrophila* WSU861044 | 2.13 | 159 | TransportDB |
| Bacteria | *Wolbachia* | *endosymbiont* of *Culex quinquefasciatus* Pel | 1.48 | 79 | TransportDB |
| Bacteria | *Wolbachia* | *endosymbiont* of *Drosophila melanogaster* | 1.27 | 83 | TransportDB |
| Bacteria | *Wolbachia* | *endosymbiont* of *Drosophila simulansw* Ha | 1.3 | 88 | TransportDB |
| Bacteria | *Wolbachia* | *endosymbiont* of *Drosophila simulansw* No | 1.3 | 80 | TransportDB |
| Bacteria | *Wolbachia* | *endosymbiont* of *Onchocerca ochengi* | 0.96 | 62 | TransportDB |
| Bacteria | *Wolbachia* | *endosymbiont* strain TRS of Brugiamalayi | 1.08 | 67 | TransportDB |
| Bacteria | *Wigglesworthia* | *glossinidia endosymbiont* of *Glossina brevipalpis* | 0.7 | 58 | TransportDB |
| Bacteria | *Wigglesworthia* | *glossinidia endosymbiont* of *Glossina morsitansmorsitans* Yalecolony | 0.72 | 57 | TransportDB |
| Bacteria | *Weissella* | *koreensis* KACC15510 | 1.44 | 166 | TransportDB |
| Bacteria | *Wolbachia* | *pipientis* wMel | 1.27 | 84 | TransportDB |
| Bacteria | *Wolbachia* | *pipientis* wPip | 1.48 | 86 | TransportDB |
| Bacteria | *Wolbachia* | *sTRS* | 1.08 | 71 | TransportDB |
| Bacteria | *Wolbachia* | *swRi* | 1.45 | 92 | TransportDB |
| Bacteria | *Wolinella* | *succinogenes* DSM1740 | 2.11 | 236 | TransportDB |
| Bacteria | *Weeksella* | *virosa* DSM16922 | 2.27 | 140 | TransportDB |
| Bacteria | *Xanthomonas* | *albilineans* GPEPC73 | 3.85 | 268 | TransportDB |
| Bacteria | *Xanthobacter* | *autotrophicus* Py2 | 5.63 | 647 | TransportDB |
| Bacteria | *Xanthomonas* | *axonopodis* pv citri str 306 | 5.27 | 341 | TransportDB |
| Bacteria | *Xanthomonas* | *axonopodis* pv citrumelo F1 | 4.97 | 326 | TransportDB |
| Bacteria | *Xanthomonas* | *axonopodis* Xac291 | 5.3 | 339 | TransportDB |
| Bacteria | *Xenorhabdus* | *bovienii* SS2004 | 4.23 | 363 | TransportDB |
| Bacteria | *Xanthomonas* | *campestris pv campestris* str 8004 | 5.15 | 326 | TransportDB |
| Bacteria | *Xanthomonas* | *campestris* pv campestris str ATCC33913 | 5.08 | 319 | TransportDB |
| Bacteria | *Xanthomonas* | *campestris* pv campestris str B100 | 5.08 | 321 | TransportDB |
| Bacteria | *Xanthomonas* | *campestris* pv raphani756C | 4.94 | 318 | TransportDB |
| Bacteria | *Xanthomonas* | *campestris* pv vesicatoria str 8510 | 5.42 | 339 | TransportDB |
| Bacteria | *Xylanimonas* | *cellulosilytica* DSM15894 | 3.83 | 457 | TransportDB |
| Bacteria | *Xanthomonas* | *citri* subsp citri Aw12879 | 5.4 | 335 | TransportDB |
| Bacteria | *Xylella* | *fastidiosa* 9a5c | 2.73 | 160 | TransportDB |
| Bacteria | *Xylella* | *fastidiosa* M12 | 2.48 | 138 | TransportDB |
| Bacteria | *Xylella* | *fastidiosa* M23 | 2.57 | 147 | TransportDB |
| Bacteria | *Xylella* | *fastidiosa* subsp fastidiosa GB514 | 2.52 | 133 | TransportDB |
| Bacteria | *Xylella* | *fastidiosa* Temecula1 | 2.52 | 137 | TransportDB |
| Bacteria | *Xanthomonas* | *fuscans* subsp fuscans | 5.09 | 334 | TransportDB |
| Bacteria | *Xenorhabdus* | *nematophila* ATCC19061 | 4.59 | 361 | TransportDB |
| Bacteria | *Xanthomonas* | *oryzae* pv oryzae KACC10331 | 4.94 | 269 | TransportDB |
| Bacteria | *Xanthomonas* | *oryzae* pv oryzae MAFF311018 | 4.94 | 293 | TransportDB |
| Bacteria | *Xanthomonas* | *oryzae* pv oryzae PXO99A | 5.24 | 296 | TransportDB |
| Bacteria | *Xanthomonas* | *oryzae* pv oryzicola BLS256 | 4.83 | 301 | TransportDB |
| Bacteria | *Yersinia* | *enterocolitica* subsp enterocolitica8081 | 4.68 | 635 | TransportDB |
| Bacteria | *Yersinia* | *enterocolitica* subsp palearctica1055Rr | 4.62 | 634 | TransportDB |
| Bacteria | *Yersinia* | *enterocolitica* subsp palearcticaY11 | 4.63 | 637 | TransportDB |
| Bacteria | *Yersinia* | *pestis* A1122 | 4.66 | 598 | TransportDB |
| Bacteria | *Yersinia* | *pestis* Angola | 4.69 | 542 | TransportDB |
| Bacteria | *Yersinia* | *pestis* Antiqua | 4.88 | 630 | TransportDB |
| Bacteria | *Yersinia* | *pestis* biovar Medievalis str Harbin35 | 4.71 | 577 | TransportDB |
| Bacteria | *Yersinia* | *pestis* biovar Microtus str 91001 | 4.80 | 603 | TransportDB |
| Bacteria | *Yersinia* | *pestis* CO92 | 4.83 | 592 | TransportDB |
| Bacteria | *Yersinia* | *pestis* D106004 | 4.81 | 623 | TransportDB |
| Bacteria | Yersinia | pestis D182038 | 4.8 | 612 | TransportDB |
| Bacteria | Yersinia | pestis KIM10 | 4.7 | 591 | TransportDB |
| Bacteria | Yersinia | pestis Nepal516 | 4.65 | 607 | TransportDB |
| Bacteria | Yersinia | pestis PestoidesF | 4.73 | 595 | TransportDB |
| Bacteria | Yersinia | pestis Z176003 | 4.73 | 587 | TransportDB |
| Bacteria | Yersinia | pseudotuberculosis IP31758 | 4.94 | 619 | TransportDB |
| Bacteria | Yersinia | pseudotuberculosis IP32953 | 4.84 | 622 | TransportDB |
| Bacteria | Yersinia | pseudotuberculosis PB1 | 4.77 | 614 | TransportDB |
| Bacteria | Yersinia | pseudotuberculosis YPIII | 4.69 | 610 | TransportDB |
| Bacteria | Zobellia | galactanivorans | 5.52 | 273 | TransportDB |
| Bacteria | Zymomonas | mobilis subsp mobilis ATCC10988 | 2.14 | 165 | TransportDB |
| Bacteria | Zymomonas | mobilis subsp mobilis ATCC29191 | 2.01 | 155 | TransportDB |
| Bacteria | Zymomonas | mobilis subsp mobilis NCIMB11163 | 2.22 | 177 | TransportDB |
| Bacteria | Zymomonas | mobilis subsp mobilis str CP4NRRLB14023 | 2.16 | 175 | TransportDB |
| Bacteria | Zymomonas | mobilis subsp mobilis ZM4ATCC31821 | 2.06 | 171 | TransportDB |
| Bacteria | Zymomonas | mobilis subsp pomaceae ATCC29192 | 2.06 | 163 | TransportDB |
| Bacteria | Zunongwangia | profunda SMA87 | 5.13 | 280 | TransportDB |
|  |  |  |  |  |  |
|  |  |  |  |  |  |

|  |
| --- |
